# Population Genomic Analysis Reveals Differential Evolutionary Histories and Patterns of Diversity across Subgenomes and Subpopulations of *Brassica napus* L.

**DOI:** 10.3389/fpls.2016.00525

**Published:** 2016-04-21

**Authors:** Elodie Gazave, Erica E. Tassone, Daniel C. Ilut, Megan Wingerson, Erwin Datema, Hanneke M. A. Witsenboer, James B. Davis, David Grant, John M. Dyer, Matthew A. Jenks, Jack Brown, Michael A. Gore

**Affiliations:** ^1^Plant Breeding and Genetics Section, School of Integrative Plant Science, Cornell University, IthacaNY, USA; ^2^Plant Physiology and Genetics Research Unit, U.S. Arid Land Agricultural Research Center, United States Department of Agriculture – Agricultural Research Service, MaricopaAZ, USA; ^3^Department of Plant, Soil and Entomological Sciences, University of Idaho, MoscowID, USA; ^4^Keygene N.V.Wageningen, Netherlands; ^5^Corn Insects and Crop Genetics Research Unit, United States Department of Agriculture – Agricultural Research Service, AmesIA, USA; ^6^Division of Plant and Soil Sciences, West Virginia University, MorgantownWV, USA

**Keywords:** *Brassica napus*, sequence-based genotyping, diversity panel, population differentiation, nucleotide diversity, site frequency spectrum, phylogenetic tree, inversion polymorphism

## Abstract

The allotetraploid species *Brassica napus* L. is a global crop of major economic importance, providing canola oil (seed) and vegetables for human consumption and fodder and meal for livestock feed. Characterizing the genetic diversity present in the extant germplasm pool of *B. napus* is fundamental to better conserve, manage and utilize the genetic resources of this species. We used sequence-based genotyping to identify and genotype 30,881 SNPs in a diversity panel of 782 *B. napus* accessions, representing samples of winter and spring growth habits originating from 33 countries across Europe, Asia, and America. We detected strong population structure broadly concordant with growth habit and geography, and identified three major genetic groups: spring (SP), winter Europe (WE), and winter Asia (WA). Subpopulation-specific polymorphism patterns suggest enriched genetic diversity within the WA group and a smaller effective breeding population for the SP group compared to WE. Interestingly, the two subgenomes of *B. napus* appear to have different geographic origins, with phylogenetic analysis placing WE and WA as basal clades for the other subpopulations in the C and A subgenomes, respectively. Finally, we identified 16 genomic regions where the patterns of diversity differed markedly from the genome-wide average, several of which are suggestive of genomic inversions. The results obtained in this study constitute a valuable resource for worldwide breeding efforts and the genetic dissection and prediction of complex *B. napus* traits.

## Introduction

*Brassica napus* L. is a global crop of considerable economic importance cultivated in many temperate regions around the world. Canola cultivars (devoid of erucic acid and with low glucosinolate levels) represent a major source of food for human and animal consumption, ranking second after soybean with 71.96 million metric tons produced worldwide in 2014–2015^1^. In addition, *B*. *napus* is cultivated for its leaves (fodder, kale) and its roots (rutabaga, swede). Complementary to food consumption, high erucic acid oil cultivars are highly suitable for a wide range of industrial applications, including production of lubricants and surfactants. *B. napus* is also considered a promising crop to produce feedstock for biofuels. When used as a rotation crop in the non-irrigated wheat belt of the US, *B. napus* could contribute to renewable fuel production and significantly reduce greenhouse gas emissions.

In contrast to most major crop species, little is known about the wild ancestors of the domesticated varieties of *B*. *napus* used today, where the species originated, and when it was first cultivated. *B*. *napus* is an allotetraploid (genome AACC, 2*n* = 4*x* = 38) and the extant species most closely related to its diploid progenitors are the cultivated *B*. *rapa* L. (Chinese cabbage, oil and turnip; AA, 2*n* = 20) and *B*. *oleracea* L. (broccoli, cauliflower, cabbage; CC, 2*n* = 18). The frequency distribution of chloroplast haplotypes among *B*. *napus* and sister taxa in its lineage strongly suggest that *B*. *napus* has a polyphyletic origin, with the initial hybridization event leading to the synthesis of *B*. *napus* occurring multiple times and possibly involving different maternal ancestors (Song and Osborn, 1992; Allender and King, 2010). The timing of the hybridization event based on divergence between orthologous gene pairs of *B*. *napus* and their respective diploid progenitors (*B*. *rapa* or *B*. *oleracea*) is estimated to have occurred between 7,500 and 12,500 years ago, a period of time that coincides with the spread of agricultural practices in the Neolithic period (Chalhoub et al., 2014). The absence of an extant wild *B. napus* tetraploid species raises the question of whether the hybridization and allopolyploid speciation occurred naturally or were the direct result of deliberate human-induced hybridization and artificial selection.

Historical dating for the initial cultivation of *B. napus* is challenging because, as with many other *Brassica* sp., it is characterized by extreme morphological variability (Prakash et al., 2011). Although mentioned in ancient Greek and Roman as well as medieval European texts, it is difficult to know whether these descriptions actually refer to *B*. *napus* or closely related species with similar morphology and usage. Only in medieval Europe do unambiguous records describe the use of *B*. *napus* as a crop (Song and Osborn, 1992; Snowdon et al., 2007; Prakash et al., 2011). Beyond Europe, the timing of *B*. *napus* introduction to Asia and North America is imprecise. Extant Chinese cultivars seem to have arrived from Europe and Canada in the 1930s (Qian et al., 2006; Chen et al., 2008). Prior to WWII, *B*. *napus* production acreage was low in the US and Canada. It became intensively cultivated as the demand for industrial oil sharply increased in 1942, but dropped again after the war (Downey, 1990; Busch et al., 1994).

The history of *B*. *napus* took a dramatic turn in the 1950s, when intense breeding efforts were undertaken in Canada to develop food-grade oil (canola) cultivars with low levels of glucosinolates, compounds unfit for livestock consumption, and no erucic acid, a 22-carbon monounsaturated fatty acid that is undesirable in high amounts in human diets (Snowdon et al., 2007). The first “double-low” cultivar, Tower, was released in 1974 (for a review, see Snowdon et al., 2007; Fu and Gugel, 2010). Since then, global canola production has grown dramatically: canola oil was the third-most produced vegetable oil globally in 2015^2^, and canola meal is currently the second most produced feed meal after soybean^3^.

Despite a recent evolutionary history, *B*. *napus* exhibits strong population structure that is partly explained by morphotype, phenology, and breeding program (Rahman, 2013). Kale and rutabagas form a group that is distinct from oilseed morphotypes (Diers and Osborn, 1994; Bus et al., 2011) and has more diversity than both fodder and oilseed types (Hasan et al., 2005). The lower diversity in oilseed types is explained by the extremely limited number of founders used to develop canola quality oilseed cultivars (Cowling, 2007; Snowdon et al., 2007; Fu and Gugel, 2010). *B*. *napus* cultivars are also structured according to their growth habit. Winter types are sown in fall and require exposure to low temperatures (vernalization) to produce flowers and seeds. In contrast, spring types are sown in spring and flower earlier, a phenotype that is probably linked to local adaptation in environments with extreme winters (Schiessl et al., 2014). Growth habit is the major determinant explaining genetic structure among *B*. *napus* accessions (Diers and Osborn, 1994; Becker et al., 1995; Bus et al., 2011; Delourme et al., 2013).

Deepening our understanding of the genome-wide levels and patterns of genetic diversity in the *B*. *napus* germplasm pool is fundamental to its effective preservation, management, and utilization. To that end, we conducted a genome-wide analysis of a panel of 782 samples highly representative of the worldwide geographic distribution of *B*. *napus*. To survey the genetic diversity present in our *B. napus* diversity panel, we sequenced each sample individually using sequence-based genotyping (SBG) and aligned the sequencing reads of each sample against the reference genome of *B*. *napus* (Chalhoub et al., 2014) to identify genetic variants. We applied stringent quality filters to the detected variants, producing 30,881 high-confidence single-nucleotide polymorphism (SNP) markers distributed across the genome. The population genetic analysis of these SNPs allowed us to infer historical relationships and characterize the distribution of genetic variation among samples. The results of this analysis also revealed distinct evolutionary histories for the A and C subgenomes. Additionally, we identified several highly differentiated regions that contain loci likely related to geographic adaptation and breeding history, as well as putative genomic inversions. Taken together, this genomic information will serve as a resource for uniting global breeding efforts and facilitating the development of locally adapted *B*. *napus* varieties.

## Results

### SBG of the *B. napus* Diversity Panel

**FIGURE 1:**
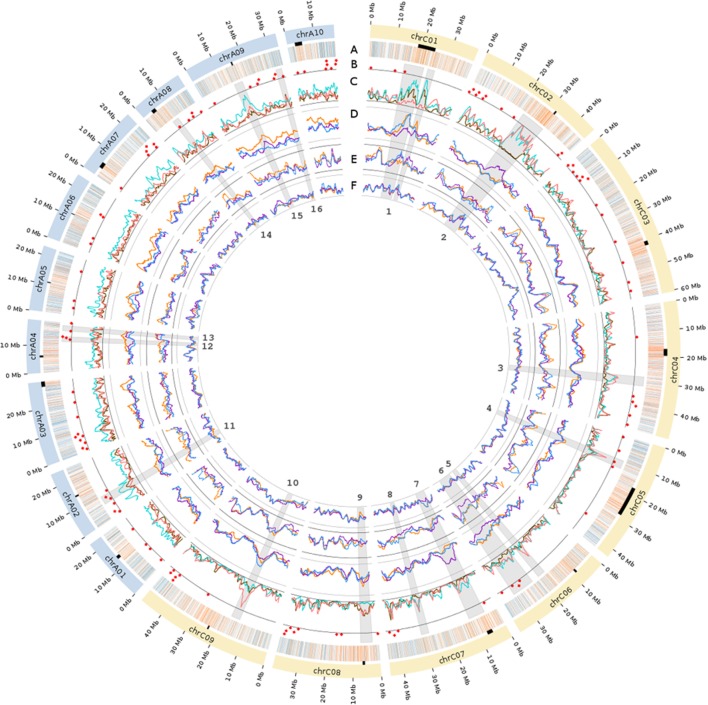
**Genome-wide SNP distribution, functional annotations and summary statistics of the genetic diversity of the *Brassica napus* diversity panel.** The colored arcs on the periphery of the circle represent the 19 *B. napus* chromosomes. The black rectangles below the chromosomes show the putative location of centromeres based on coordinates provided in Mason et al. (2015). Track A: Distribution of the genic (blue) and intergenic (orange) SNPs used in this study. Track B: The red dots indicate the genomic positions of flowering time and vernalization genes. Track C: Pairwise Fst of SNPs are represented in pink for winter Asia (WA) vs. spring (SP), in brown for WA vs. winter Europe (WE), and in turquoise for WE vs. SP. Tracks D: SNP-based average number of pairwise mismatches (Π). Track E: Proportion of heterozygous samples (heterozygosity). Track F: SNP call rate for WA (orange), SP (purple), and WE (blue). The scale for each track is marked by two concentric lines representing the minimum and maximum value for each track (track C: 0.0 to 50.1%; track D: 0.7 to 24.3%; track E: 0.2 to 4.8%; track F: 16.2 to 65.4%). The gray wedges in radial orientation each represent a region of interest (ROI) numbered from 1 to 16 in the inner circle. Exact coordinates of the ROIs are provided in **Supplementary Table S2**. An expanded view of tracks C–F is available in **Supplementary Figures S6**, **S7**, and **S11**.

After filters were applied to SNPs and samples, the final *B*. *napus* data set included a total of 30,881 high-confidence SNP markers scored on 782 samples. These 30,881 SNPs were evenly distributed along the 19 *B*. *napus* chromosomes, with an average of one SNP every 27,534 bp (**Figure 1**, track A). The A subgenome contained 38.8% of the SNPs, and the C subgenome the remaining 61.2% (**Supplementary Table S1**). These values conform to the expected distribution based on subgenome size, as the A subgenome is substantially smaller (∼315 Mb) than the C subgenome (∼527 Mb), and represents only ∼37.5% of the *B. napus* reference genome assembly. However, while 23.2% of the current *B*. *napus* reference genome (v4.1) is annotated as a “gene,” 44.0% (13,573) of the SNPs in our data set were located in these regions, thus representing an almost twofold enrichment of SNPs in genic regions. The SNP call rate (i.e., the percentage of samples successfully genotyped per SNP) distribution had a mean value of 35.2% (corresponding to 275 samples genotyped) and a median of 23.3% (**Supplementary Figure S1A**). The mean and median sample call rate (i.e., percentage of SNPs successfully genotyped for each sample) was 35.2% (10,859 SNPs genotyped) and 30.3%, respectively (**Supplementary Figure S1B**).

### Population Structure and Differentiation

**FIGURE 2:**
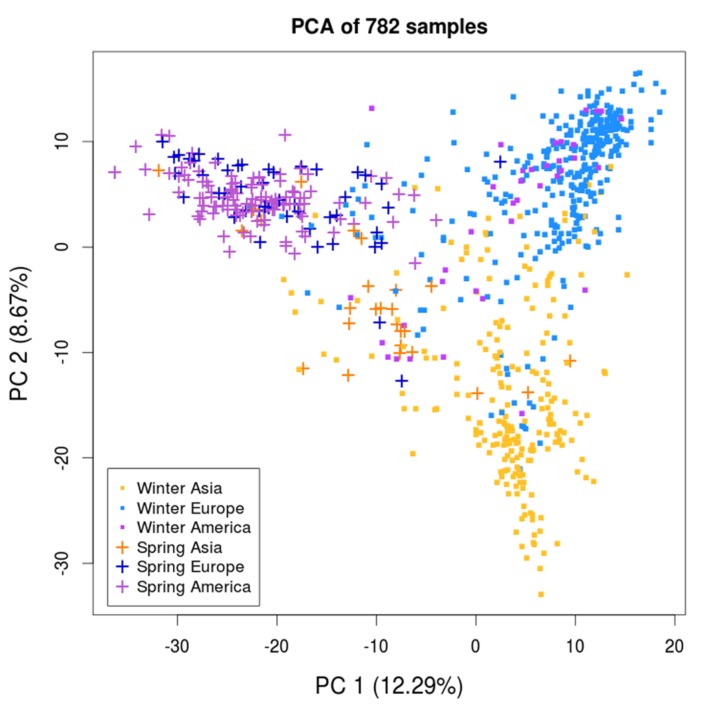
**Principal component analysis of the *B. napus* diversity panel of 782 samples.** The principal component analysis (PCA) reveals three clusters that are each defined as a major subpopulation: winter Europe (WE), winter Asia (WA), and spring (SP). The first two principal components (PCs) are represented. Samples are labeled according to their growth habit and geographic origin.

To examine the presence of population structure among the 782 samples, we performed a principal component analysis (PCA) using the 30,881 SNP markers. The samples were labeled according to their growth habit and geographic origin, and distributed as follows: 347 winter Europe (WE), 212 winter Asia (WA), 38 winter America (WAm), 47 spring Europe (SE), 26 spring Asia (SA), and 112 spring America (SAm). The PCA revealed three major clusters (**Figure 2**), with the highest variance axis (PC1) mainly separating spring and winter samples, and the second highest variance axis (PC2) splitting WA samples from other samples. In contrast to the winter types that could be separated into European and Asian clusters, all of the spring types (SE, SA, SAm) formed a single cluster independent of their geographic origin (Europe, Asia, or America). Therefore, we considered all of the spring types (*n* = 185) as a single subpopulation (spring, SP). The structure observed on the PCA enabled us to define three major subpopulations: WE, WA, and SP. We noticed that the WAm samples did not assemble into a discrete cluster and intermixed with the WE and WA clusters. Therefore, unless specified otherwise, the 38 WAm samples were not included in the analyses involving the three major subpopulations.

After using the PCA to define the three major subpopulations, we examined the 300 SNPs (∼1% of all the SNPs) with the highest principal component (PC) loadings to establish whether specific genomic regions were disproportionately responsible for splitting samples along the PCs. On PC1, we uncovered two major genomic regions with blocks of consecutive SNPs resulting in elevated PC loadings (**Supplementary Figure S2**). One ∼8 Mb region was located on chromosome 1 of the C subgenome (**Figure 1**, ROI 1) and another ∼10 Mb region on chromosome C02 (**Figure 1**, ROI 2). While PC2 also showed some SNPs with elevated loadings, unlike PC1, these SNPs were not found to be clustered within any particular genomic region. Removing these two regions did not changed the overall distribution of the samples on the PCA (data not shown), and thus did not affect the definition of the three subpopulations.

To quantify the genetic differentiation between the three subpopulations detected in the PCA, we estimated average pairwise Fst between WA, WE, and SP. Plotting Fst estimates for each SNP along the genome, we observed several genomic regions with elevated Fst (**Figure 1**, track C). Of the 1005 SNPs in regions with extreme Fst values (i.e., greater than 3 standard deviations above the genome-wide average), 96.6% (971 SNPs) were located in the C subgenome. For this reason, instead of calculating a genome-wide average Fst, we estimated the average Fst for each subgenome separately. In both subgenomes, the average Fst was the highest when SP was compared to WE and WA (**Table 1**). This result is in agreement with the PCA performed for each subgenome separately (**Supplementary Figure S3**), where the variance explained by PC1 (splitting SP vs. the winter subpopulations) was higher in the C subgenome. Despite different amounts of differentiation between subgenomes, the overall shape of the PCA plot for each subgenome separately was the same as that of a PCA plot for both subgenomes together (**Figure 2**). Because the SP differentiation was slightly higher in the C subgenome, we also estimated Fst after excluding SNPs located on chromosome C02 in the ∼10 Mb region with extreme differentiation between SP and winter subpopulations (**Figure 1**, ROI 2). The resulting Fst estimates were only modestly decreased (**Table 1**), suggesting that the high differentiation of SP did not seem to be driven by outlier regions of the genome.

**Table 1:** Pairwise Fst values between the three major subpopulations: winter Europe (WE), winter Asia (WA), and spring (SP).

Subpopulation pair	SNP ascertainment subpopulation	Fst A subgenome	Fst C subgenome	Fst C subgenome (filtered)^∗^
WA-SP	SP	0.223	0.278	0.263
WA-SP	WA	0.214	0.266	0.251
WE-SP	SP	0.214	0.260	0.243
WE-SP	WE	0.216	0.258	0.241
WA-WE	WA	0.181	0.181	0.183
WA-WE	WE	0.178	0.181	0.184


*^∗^Fst for the C subgenome was estimated after removing a ∼10 Mb region on chromosome C02 that was extremely differentiated between spring (SP) and the winter subpopulations.*

### Diversity Sharing and Subpopulation-Specific Alleles

In addition to estimating population differentiation, we estimated how much of the total genetic diversity observed in our data set was shared among the three subpopulations (WA, WE, and SP). For each SNP, we determined whether the minor allele was observed exclusively in a single subpopulation, or more than one subpopulation. We focused on the minor allele (the allele with the lowest frequency when considering the three major subpopulation together) because, if favorable, this is the allele most likely to be an underutilized source of genetic variation in *B*. *napus* breeding programs. Given that there were 1.58 times more SNPs called in the C subgenome (**Supplementary Table S1**) and the possibility that these SNPs could dominate the genome-wide signal, we analyzed each subgenome separately.

**FIGURE 3:**
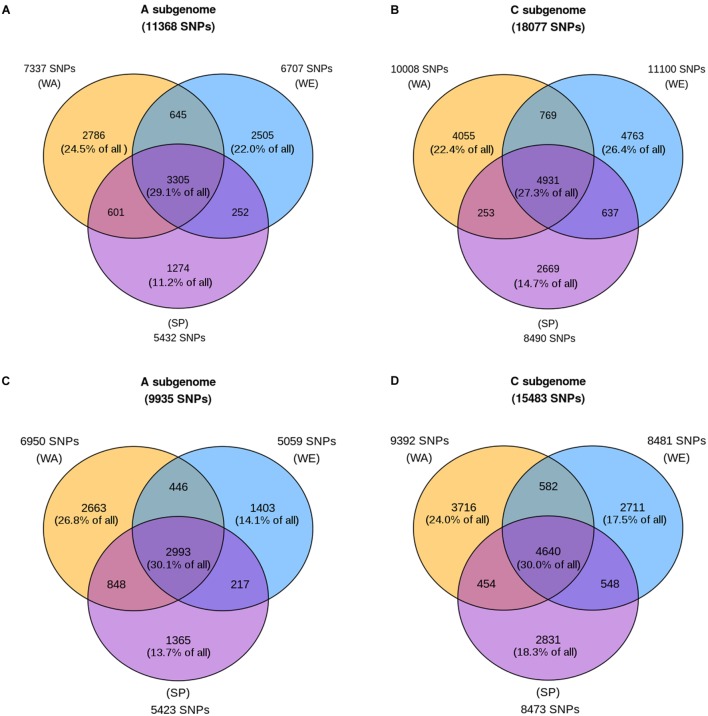
**Shared and subpopulation-specific polymorphism in the three major subpopulations.** Each circle of the Venn diagram represents a subpopulation: winter Europe (WE), winter Asia (WA), and spring (SP). Counts adjacent to each circle are the number of SNPs with at least one copy of the minor allele in the subpopulation. For each subpopulation, these counts are further decomposed into three categories, depending on whether the minor allele is also present in one or two additional subpopulations (counts inside the Venn diagram). These counts are also expressed as percentages of the total SNP count across the three subpopulations (value given at the top of each panel). The top two panels **(A,B)** show values for the entire sample size for each subpopulation, while the bottom two panels **(C,D)** show the average counts after 100 random downsamplings of WE and WA to 185 samples each (matching the sample size of SP). The winter America subpopulation is not included in this analysis due to its small sample size.

The results show that less than a third of the SNPs (29.1 and 27.3% in the A and C subgenome, respectively) were polymorphic in all three subpopulations (**Figures 3A,B**, central intersection). In contrast, a large proportion of SNPs were polymorphic in only one subpopulation (subpopulation-specific SNPs). Our results also show that SP was the subpopulation with the lowest number of SNPs overall (*n* = 5432 and *n* = 8490 in the A and C subgenome, respectively). The two winter subpopulations have similar numbers of polymorphic SNPs. Specifically, of all SNPs present in the A subgenome, 22.0% were found only in WE, 24.5% were WA-specific, and 11.2% were SP-specific, for a total of 57.7% subpopulation-specific SNPs (**Figure 3A**). In the C subgenome, 26.4% were found exclusively in WE, 22.4% were found only in WA, and 14.7% were SP-specific, representing a total of 63.5% subpopulation-specific SNPs (**Figure 3B**). Given that the reference genome is of winter type, we considered the possibility that the high number of SNPs observed in the winter subpopulations could be explained by ascertainment bias. The analysis of sample call rate for each subpopulation shows the lowest average sample call rate in WA compared to SP and WE (**Supplementary Figure S4**), ruling out ascertainment bias as the reason for the high number of SNPs observed in winter subpopulations.

To examine the effect of sample size on these results, we repeated the analysis after downsampling the 347-sample WE and 212-sample WA subpopulations to 185 samples each to equal the SP sample size. With this analysis, the number of SNPs polymorphic in WE decreased to about the same level as SP, and much below that of WA (**Figures 3C,D**). In the A subgenome, 14.1% of the SNPs were WE-specific, 13.7% were SP-specific, and 26.8% were exclusive to WA. In the C subgenome, only 17.5% of all SNPs were WE-specific, compared to 18.3% for SP-specific and 24.0% for WA-specific SNPs. This indicates that the initial SNP count in WE (**Figures 3A,B**) was driven by the higher sample size of this subpopulation. The dramatic decrease in genetic variation present in WE when its sample size was reduced by almost half also indicates that minor alleles in WE tend to be present in only a few samples in the subpopulation.

### Phylogenetic Relationships between Subpopulations

**FIGURE 4:**
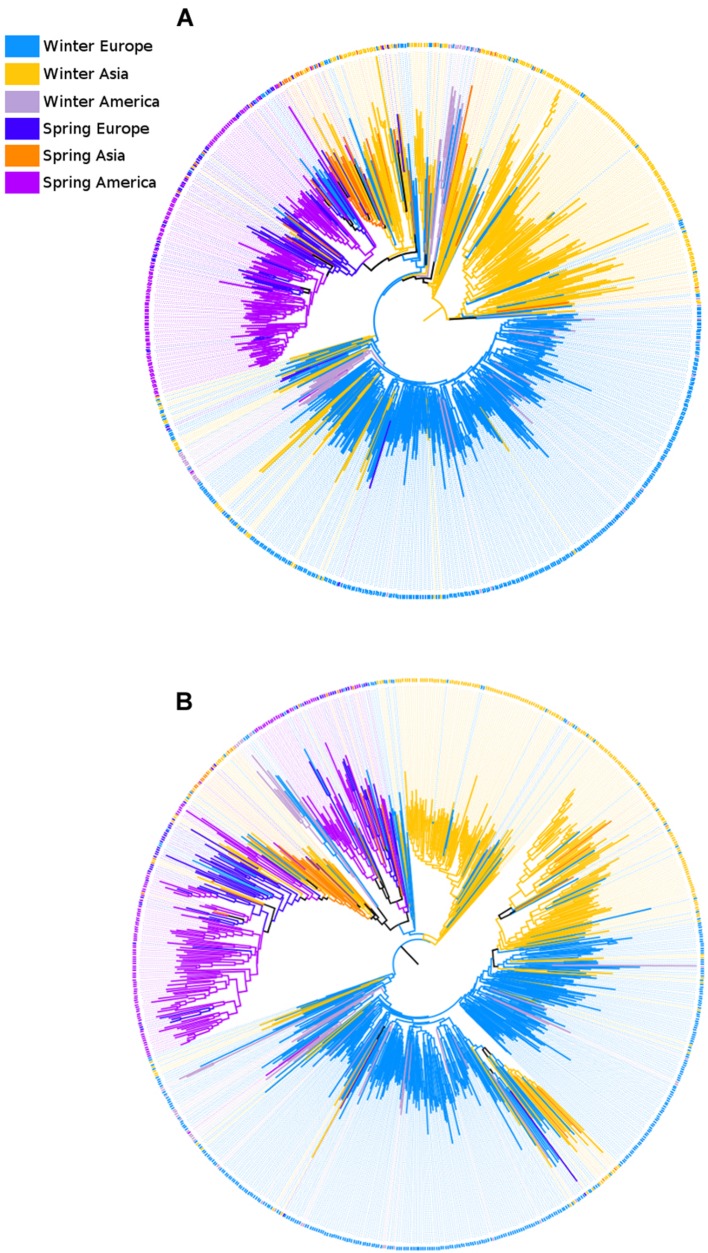
**Neighbor-joining trees illustrating phylogenetic relationships among the 782 *B. napus* samples.** Both cladograms were mid-point rooted. The A subgenome **(A)** and the C subgenome **(B)** show different topologies. In the A subgenome, most winter Asia samples are located outside the clades containing winter Europe and spring samples. This indicates that the A subgenome has a larger proportion of SNPs that are WA-specific (i.e., not found in other subpopulations). In contrast, WE samples tend to have a more basal location in the neighbor-joining tree for the C subgenome.

To examine the phylogenetic relationship between the *B. napus* samples we used a neighbor-joining (NJ) approach (**Figure 4**). Because this analysis considered all samples independently rather than grouping them by subpopulation, sample size heterogeneity among subpopulations did not matter. Therefore, we used all 782 samples (including samples of WAm) and full geographic information for the spring samples (splitting SP into three groups based on geographic origin: Asia, Europe, and America). Using a mid-point rooting (MPR) method (Hess and De Moraes Russo, 2007) to place the root on the tree for each subgenome, two striking observations emerged. First, the NJ trees for the A and the C subgenome showed different topologies, with the root being located among WA samples in the A subgenome (**Figure 4A**), and among WE samples in the C subgenome (**Figure 4B**). Second, despite observing large groups dominated by samples of the same growth habit, none of the major subpopulations was monophyletic. These apparent discrepancies between clustering based on genetic data and subpopulation classification may reflect recent gene flow during breeding history. Alternatively, this may also be the result of uncertainty in the relationship between samples, as illustrated by relatively low bootstrap support (averaging 70.0 and 72.2% across all nodes in the A and C subgenome, respectively). Notably, the low-support nodes were mostly internal nodes near the base of the tree, where bootstrap support dropped to 40.0 and 44.3% for the A subgenome and C subgenome, respectively. This indicates that while the NJ approach clustered individual samples within each subpopulation effectively, it did not confidently resolve the position of the different subpopulations relative to each other.

### Distribution of Polymorphism within Subpopulations

We explored the distribution of allelic diversity within each subpopulation to further investigate the evolutionary history of *B*. *napus* subpopulations. Here, we used only the three subpopulations (WE, WA, and SP) with large sample sizes. First, we estimated the average nucleotide diversity (π) for each subpopulation and subgenome (**Table 2**). In the A subgenome, π was higher in WA (πA = 0.00230) than in the WE (πA = 0.00189) and SP (πA = 0.00208) subpopulations. In the C subgenome, SP had a higher estimate of nucleotide diversity (πC = 0.00227) than WA (πC = 0.00196) and WE (πC = 0.00198). To understand these differences, we calculated the average number of nucleotide mismatches for each of the SNPs (Π) and plotted these statistics along the genome (**Figure 1**, track D). Interestingly, Π along the A subgenome was systematically elevated in WA, explaining the high π value for the A subgenome of WA. In contrast, the C subgenome of SP contained only a few regions, particularly on chromosomes C02 and C07, where SNP-wise Π was elevated. To examine whether the SNPs in these regions may have biased the average subgenome estimate of π, we recalculated π for the C subgenome of SP after filtering out these two regions. The estimate of π thus obtained was lower (π = 0.00218), but still remained higher than in WE and WA.

**Table 2:** Genome-wide average nucleotide diversity estimates (π) and Tajima’s *D*-values for the three major subpopulations: winter Europe (WE), winter Asia (WA), and spring (SP).

Subpopulation	π (A subgenome)	π (C subgenome)	Tajima’s D (A subgenome)	Tajima’s D (C subgenome)
WE	0.00189	0.00198	–4.70	–3.16
WA	0.00230	0.00196	–1.96	–3.36
SP	0.00208	0.00227	–3.42	–1.03


**FIGURE 5:**
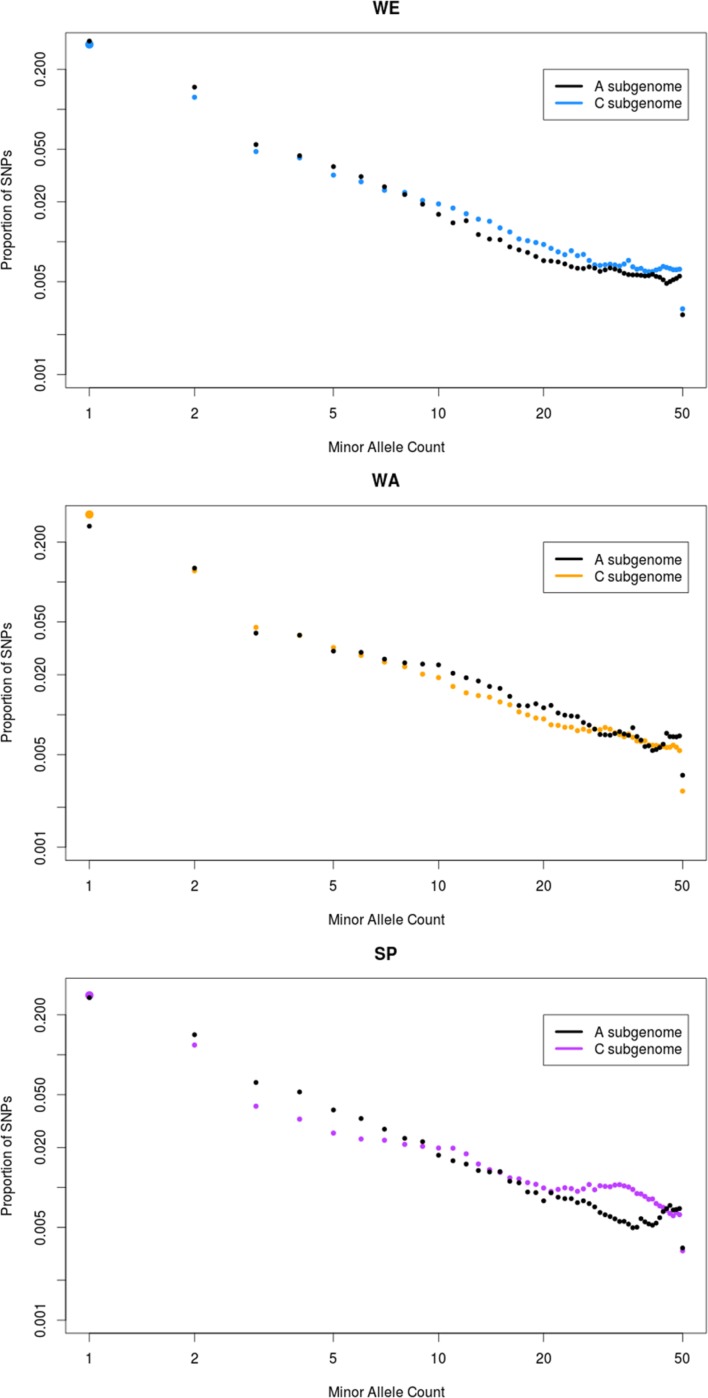
**Folded site-frequency spectrum (SFS) of the three major subpopulations.** The analysis was conducted on each of the three major subpopulations: winter Europe (WE), winter Asia (WA), and spring (SP). The *x*-axis represents in log scale the number of copies of the minor allele observed for a SNP (minor allele count, MAC) in the subpopulation. The *y*-axis represents in log scale the proportion of SNPs in the subpopulation for all MAC. The black dots show the SFS for the A subgenome, while the SFS for the C subgenome is shown in other colors. In each subpopulation, all the SNPs were probabilistically downsampled to 50 genotypes to allow comparison of three major subpopulations despite their different sample size, and to avoid distortions due to an unequal amount of missing data across sites (see Materials and Methods).

Secondly, we generated a folded site frequency spectrum (SFS) for both subgenomes of each subpopulation by counting the proportion of SNPs with different minor allele count (MAC; **Figure 5**). In WE, the SFS of the A subgenome was shifted toward singletons (i.e., alleles with MAC of 1) and rare variants (MAC < 9), compared to the C subgenome that had more intermediate frequency variants (MAC > 9). This was exactly the opposite in WA, where the C genome was shifted toward low frequency variants and the A genome had more frequent variants. The SFS of SP overall resembled that of WE, with two differences. First, singletons were more frequent in the C than in the A subgenome of SP. Second, the depletion of rare variants and the excess of common variants in the C subgenome were more accentuated in SP than in WE. This was especially visible in variants with MAC > 21. To further investigate the possible cause of this pattern, we filtered out the ∼10 Mb region on chromosome C02 and the 6.5 Mb region on chromosome C07 that had strikingly high nucleotide diversity in SP (**Figure 1**, track D). The resultant SFS in SP showed a modest but clear decrease in the number of SNPs with MAC > 21 (**Supplementary Figure S5**). This suggests that these two regions partially, but not entirely, explain the excess of intermediate frequency variants in the C subgenome of SP.

To further interpret the global shape of the SFS, we calculated Tajima’s D for the three major subpopulations in the A and C subgenomes (**Table 2**). All subpopulations and subgenomes had negative values, indicating a global deviation from a neutral model of evolution. Both SP and WE had more negative values in the A subgenome than the C subgenome (**Table 2**), reflecting the stronger skew toward low frequency variants in the A subgenome observed in the SFS. The C subgenome of SP had the least negative value of all subgenomes and all subpopulations (**Table 2**), in agreement with the excess of common variants observed on the SFS. In contrast, Tajima’s D was more negative for the C subgenome than for the A subgenome in the WA subpopulation, which confirmed that the C subgenome had more rare variants than the A subgenome in WA. To examine whether the shape of the various SFSs was influenced by strong selective pressures or demographic events, we ran the same analysis on intergenic SNPs alone, which are expected to be evolving under lower selective pressure. For all three major subpopulations, the SFSs based on intergenic SNPs were very similar to the SFSs with all SNPs (results not shown), suggesting that allele frequencies in coding regions were not the main contributors to the overall SFS patterns.

### Genetic Diversity and Differentiation in Flowering Time and Vernalization Genes

Because population structure in *B. napus* is strongly associated with growth habit, we tested whether the set of 117 major flowering time and vernalization genes (as defined in Delourme et al., 2013 and Chalhoub et al., 2014) were associated with systematic changes in nucleotide diversity and Fst compared to the subgenome-wide averages. In SP and WE, permutation tests revealed no significant difference (*P* > 0.01) in Π for the SNPs located in or immediately flanking the set of 117 genes compared to the genome-wide average. In WA, however, Π was significantly higher in the gene set (*P* = 0.0089, in 10,000 permutations). Fst between all pairs of subpopulations was significantly higher for the set of 117 genes compared to the genome-wide average (*P* ≤ 0.0047 in 10,000 permutations). This is despite the fact that only one gene out of 117 was located within the two large regions with extreme Fst on chromosomes C01 and C02 (**Figure 1**, track B).

### Genomic Regions with Major Differences in Allele Frequency among the Three Subpopulations

We also used per-SNP estimates of Fst and Π to identify genomic regions that markedly differed from the subgenome average (**Figure 1**, tracks C,D and **Supplementary Figures S6** and **S7**). Of these regions, we selected a subset of 16 of the most biologically interesting ones for further exploration (see **Supplementary Table S2** for the coordinates of these regions). Among these 16 regions of interest (ROI), ROI 1 and ROI 2 correspond to the two genomic regions with the highest loadings on PC1 (**Supplementary Figure S2**), which prompted us to re-examine the PCA results for the 16 ROIs.

**FIGURE 6:**
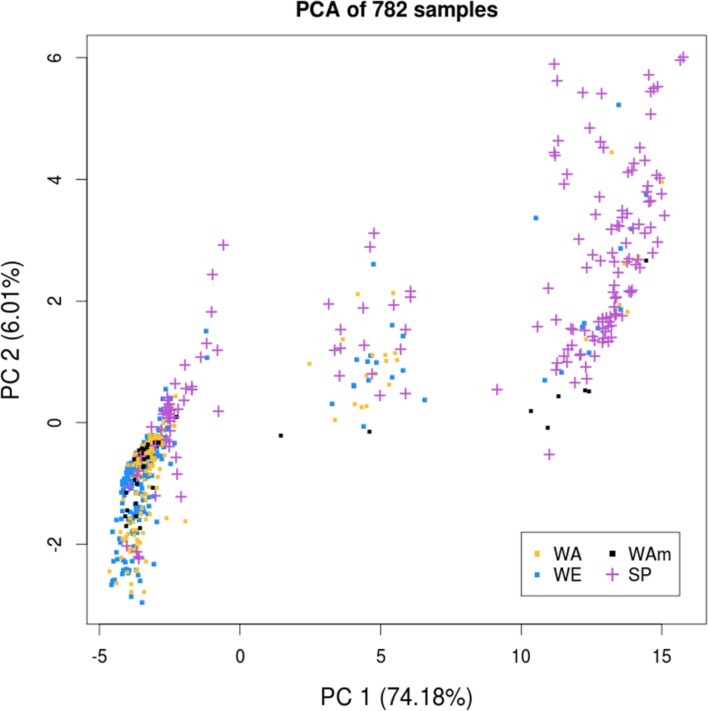
**Principal component analysis of all 782 samples for the SNPs located within the putative genomic inversion on chromosome C02.** The principal component analysis (PCA) was conducted using only the SNPs located between 23,325,687 and 32,393,406 bp on chromosome C02, corresponding to region of interest ROI 2. The first two PCs are represented. Samples are labeled according to membership in each of the three major subpopulations: winter Europe (WE), winter Asia (WA), spring (SP). Samples of winter America (WAm) which do not belong to any of the three major subpopulations are also shown.

Starting with the region that had the highest loadings (ROI 2), we first performed a PCA with all the samples, but using only the SNPs from ROI 2 (on chromosome C02 between 23,325,687 and 32,393,406 bp; **Figure 6**). In this PCA, we observed that samples clustered in a three-band pattern. Such a pattern is consistent with the presence of two extremely long, non-recombining haplotypes in the subpopulations, as typically observed in genomic regions where a chromosomal inversion is polymorphic in the subpopulation (Ma and Amos, 2012; Kemppainen et al., 2015). In this scenario, the two clusters on each side of PC1 typically contain samples with genomic regions of opposite orientation, with the middle cluster denoting carriers of both haplotypes (heterozygotes for the inversion). While SP appeared to carry both haplotypes, one of the haplotypes had a much lower frequency in the two winter subpopulations.

Following the same approach, we examined the other 15 ROIs, performing a PCA on the subset of SNPs located within each ROI alone (**Supplementary Figure S8**). We observed a discrete three-band pattern on the PCA for six additional regions (ROIs 1, 6, 11–14). The interpretation of this pattern is ambiguous for the four smaller regions (ROIs 11–14 of 3.4 Mb or less) because of the small number of SNPs in these regions. The two largest regions however, ROI 1 on C01 (10.4 Mb) and ROI 6 on C06 (8.8 Mb), showed a more reliable signal and as such are stronger candidates for chromosomal inversions. Notably, ROI 1 corresponds to the second genomic region with the highest PC loadings on the genome-wide PCA (**Supplementary Figure S2**).

The nine other ROIs did not show a pattern characteristic of genomic inversions, but the diversity and differentiation of the SNPs in these regions seem to indicate that they have experienced a different evolutionary history compared to the genome average. For example, while SP was the most differentiated subpopulation genome-wide (**Table 1**), in ROI 4 (C05), ROI 5 (C06), and ROI 10 (C09), WA showed a stronger differentiation from the other two subpopulations (WE and SP). In ROI 7 (C07), SP did not show a strong differentiation from the two winter subpopulations (**Figure 1**, track C and **Supplementary Figure S6**), but instead had a much higher nucleotide diversity than WE and WA (**Figure 1**, track D and **Supplementary Figure S7**). Such regions may harbor loci of adaptive importance that have driven changes in their nucleotide diversity and allele frequency.

## Discussion

We used SBG to characterize the genomic diversity in 782 *B. napus* samples encompassing both spring and winter growth habits and including a comprehensive representation of the global diversity for this species. Due to the high identity between homeologous chromosomes in the two subgenomes, we applied stringent criteria to align the SBG reads against the *B. napus* genome, followed by strict quality control filters on variant and genotype calls. This allowed us to score 30,881 high-quality SNPs distributed across the entire genome, with an average density of 1 SNP per 27.5 kb. This value is comparable or superior to the post-processing marker density achieved with existing commercial arrays for *B. napus* (e.g., Hatzig et al., 2015). In addition, SBG also allows *de novo* discovery of variants and significantly reduces ascertainment bias typically associated with commercial SNP genotyping arrays (Poland and Rife, 2012). By using PstI in library preparation, we preferential genotyped undermethylated regions of the genome likely to contain transcription-enabled genes (see Liou et al., 1996 and references within), accounting for the enrichment of SNPs in genic regions. At the same time, more than half of the SNPs in the present study are intergenic, allowing queries of often underexplored genomic regions. However, due to the recent divergence of the two parental genomes (A, from *B. rapa* ancestor and C from *B. oleracea* ancestor), about 30% of the reads did not uniquely align to the reference genome and were not used for variant and genotype calling. As a result, although the levels of diversity observed in our study were high, the true levels may be underestimated.

### Population Structure and Differentiation

A PCA of 782 samples revealed the existence of strong population structure, with the presence of three major genetic groups. Similar observations had been previously reported by several authors (Diers and Osborn, 1994; Cruz et al., 2006; Qian et al., 2006, 2014; Bus et al., 2011; Delourme et al., 2013). These groups were primarily separated by growth habit (PC1, separating spring and winter types) and secondarily by geographical origin (PC2, separating Asian and European winter types). We therefore considered the three distinct major genetic groups to be primarily associated with their respective descriptors (SP, WE, and WA) and we classified the samples into three subpopulations based on these descriptors. Despite the strong association between genetic groups and the combination of growth habit and geographical origin, this association is not complete, nor would we expect it to be. First, the geographic designation for the origin of samples was assigned based on breeding records and accession databases. Given the extensive exchange of cultivars between breeding programs during modern *B. napus* breeding, we occasionally expect genotypes originating from one geographic region to be labeled as a different geographic origin. Second, given that the growth habit is likely determined by a limited number of loci, we expect some samples of a given growth habit to have a genetic background similar to those typical of the other growth habit. Since our data surveyed the genome-wide genetic makeup of the samples, clusters on the PCA are expected to primarily reflect genetic similarities and differences among samples. Together, this explains why there is not a complete genetic differentiation observed among the three major subpopulations we describe.

In order to quantify the genetic differentiation among the groups, we estimated pairwise Fst for each pair of subpopulations. Overall, our Fst estimates were comparable to previously published values (Bus et al., 2011; Delourme et al., 2013). In agreement with the PCA results, Fst was higher when spring was compared to either of the two winter subpopulations, indicating a spring-specific differentiation. The SP differentiation was markedly higher in the C than in the A subgenome, whereas Fst between WE and WA was the same for both subgenomes. The Fst in SP remained high even after removing a ∼10 Mb region with extreme differentiation on chromosome C02, suggesting that the high differentiation of SP was not driven by an extreme outlier genomic region. Altogether, these results could indicate that SP is an ancestral group to winter types, with a subsequent split of WE and WA, explaining the lower differentiation of the two winter subpopulations. Alternatively, they may reflect a stronger change in allele frequency during the evolutionary history of SP due to severe bottlenecks or breeding practices. To distinguish between these two scenarios, we examined the relationship between samples and subpopulations in a phylogenetic analysis.

### Phylogenetic Relationship between Samples and Subpopulations

We used a NJ tree to examine the overall relationship between samples and refine the evolutionary history of *B. napus.* Despite the clear population structure observed in the PCA, none of the major subpopulations were completely monophyletic in the NJ trees of the A and C subgenomes. This result is in agreement with previous studies showing at least three distinct groups within Asian oilseed cultivars (Qian et al., 2014), and polyphyletic groups in clustering analyses (Hasan et al., 2005). In addition, chloroplast and mitochondrial data support multiple origins of *B. napus* (Song and Osborn, 1992; Allender and King, 2010). Another interesting observation is that, based on a MPR method to root the NJ trees, we observed different topologies for the A and C subgenome. In the A subgenome, the topology placed most WA samples at the root of the tree. This result most likely reflects the intense use of inter-specific crosses between *B. rapa* and *B. napus* in the Chinese breeding programs (Diers and Osborn, 1994; Qian et al., 2006; Chen et al., 2008; Rahman, 2013) that has introduced new alleles into the A subgenome of WA accessions. In the C subgenome, the majority of the WE samples were located at the base of the tree, suggesting that WE is the most likely outgroup for the other subpopulations. In both subgenomes, the best model never placed SP at the root of the tree, rejecting the hypothesis of SP as an ancestral group and supporting the existence of one or more genetic bottlenecks in the evolutionary history of SP.

We should note that, although previous studies have shown that MPR methods generally place the root consistently with the most likely evolutionary history (Hess and De Moraes Russo, 2007), MPR makes assumptions that may be violated in our diversity panel. In addition, the basal nodes for the NJ tree had low bootstrap support and, as such, results should be interpreted with caution. The difference in confidence between basal and external nodes can be attributed to the fact that missing genotypes are not the same across samples, and pairwise distance is then calculated from a different set of loci. These loci represent contrasting (and at times conflicting) evolutionary histories. This bias is expected to be negligible for a pair of closely related samples, as the vast majority of their genomic loci will have a shared evolutionary history. However, this bias is expected to become more pronounced as the samples compared become more distant (i.e., for more internal nodes in the tree). The effect of conflicts between trees from different loci has been extensively documented in the phylogenetic literature as the gene-tree/species-tree discordance phenomenon (e.g., Nakhleh, 2013; Roch and Warnow, 2015).

### Allele Sharing and Subpopulation-Specific Variation

We also investigated *B. napus* diversity by estimating the extent to which SNPs are shared across the three subpopulations. To that end, we counted the number of SNPs that are polymorphic in a single subpopulation (i.e., that have at least one copy of the minor allele) and SNPs that are polymorphic within multiple subpopulations (i.e., where the minor allele is present in more than one subpopulation). Our results show that only about a third of the SNPs are polymorphic in all three subpopulations. Once corrected for differences in subpopulation size, SP and WE were found to have an equivalent level of diversity. This was true whether we counted the total number of polymorphic SNPs in each subpopulation, or the number of polymorphic SNPs exclusively found in one subpopulation (subpopulation-specific SNPs). Previous work reported a lower genetic diversity in spring types (Hasan et al., 2005). However, this result was contested by Bus et al. (2011), who observed that winter oilseed types had lower numbers of alleles and type-specific loci than spring oilseed rape. All these previous studies used a restricted set of molecular markers. Our estimates, based on genome-wide SBG data, suggest that there is a comparable number of SNPs polymorphic in SP and WE. Importantly, our analysis also revealed that WA was the subpopulation with the highest number of SNPs polymorphic within the subpopulation, especially in the A subgenome. This result is likely to be the hallmark of *B. rapa* introgression into the *B. napus* A subgenome in WA accessions, conferring WA alleles that are not present in any other group. A similar signal is also visible on the PCA published by Delourme et al. (2013), where the proportion of variance explained by PC1 (splitting mainly spring Asian and winter non-Asian cultivars) is almost twice as high in the A subgenome than in the C subgenome.

### Intra-subpopulation Allelic Distribution

In addition to comparing the level of variability present in the three subpopulations, we also examined the distribution of variants among individual samples within each subpopulation. To make data fully comparable across subpopulations, we accounted for both the difference in sample size among subpopulations and the unequal levels of missing data across SNPs by using a probabilistic downsampling method. Differences in sample size among populations affect the power to capture rare variation, and hence the shape of the SFS (Keinan and Clark, 2012). For similar reasons, an uneven distribution of missing data across genomic regions can distort the SFS. Effectively, we produced the SFS in each of the three major subpopulations for a subset of 50 samples with an allelic distribution as in the initial subpopulation. Our results show that all SFSs are skewed toward low frequency variants, as indicated by negative Tajima’s *D*-values for all subpopulations and subgenomes. Negative Tajima’s *D*-values are suggestive of a bottleneck, but their interpretation is complex. Strong or old bottlenecks will tend to produce negative values because alleles arising in the recovery phase are recent and have low frequency (Barton, 1998; Depaulis et al., 2003). In contrast, frequency spectra after more recent and weaker bottlenecks tend to show an excess of intermediate frequency variants and can create positive Tajima’s *D*-values. This is because the reduction in population size does not remove most existing segregating variants but can modify their distribution (Galtier et al., 2000; Depaulis et al., 2003). If the bottleneck is recent though, no new mutations have arisen since the population size shrank and there is no statistical power to detect any departure from a neutral model (Depaulis et al., 2003). Importantly, selective sweeps can affect genealogies in a similar way as bottlenecks and also produce negative Tajima’s *D*-values (Barton, 1998; Galtier et al., 2000; Nielsen et al., 2007). Demographic events have a genome-wide effect while selective events create genomic heterogeneity. Therefore, distinguishing between the two events is in theory possible, but it requires rigorous testing (Galtier et al., 2000). Although some authors could successfully demonstrate the presence of a genome-wide effect of selective pressure linked to domestication in rice (Caicedo et al., 2007), it is generally difficult to differentiate demographic and selective events in crop domestication processes (Hamblin et al., 2006) even when the population is clearly not at equilibrium. In addition, genotyping by sequencing (GBS) data are poorly suited to detect selective events, even for hard-sweeps with a SNP density of 1 SNP per 5 kb and normal levels of recombination (Tiffin and Ross-Ibarra, 2014). The data in the present study were generated with SBG, a technology extremely similar to GBS, and are very likely to suffer from the same weakness.

Nevertheless, the skew of the SFS provides interesting information to develop strategies for collecting, conserving and utilizing germplasm collections. The Tajima’s *D*-values were the most negative for WE, especially in the A subgenome. This implies that WE accessions tended to carry more very rare and unique alleles compared to the other subpopulations. In contrast, the C subgenome of SP had the least negative Tajima’s *D*-value of all subpopulations, and its SFS showed a much higher number of variants with intermediate frequency compared to the A subgenome. This result is in agreement with the genome-wide study of Delourme et al. (2013) who found that spring types had a higher polymorphic information content (PIC) than winter types. Because PIC is maximized when the frequency of the alleles is identical (50% for a SNP in a diploid genome), the lower PIC in winter types agrees with our observation of an average lower allele frequency of minor alleles in WE.

These differences in the distribution of genetic diversity among samples become important when contrasted with the analysis of subpopulation-specific SNPs. Above, we reported that at equal sample size SP and WE had a similar number of subpopulation-specific variants. However, the analysis of the SFS and Tajima’s *D* reveals that the distribution of these minor alleles is different in these two subpopulations. This suggests that the genetic variation present in WE is on average present in fewer accessions, and therefore more prone to be lost from the germplasm gene pool without appropriate management of genetic resources. In support of this claim is the dramatic effect of downsampling on the number of SNPs in WE. By reducing the number of WE samples by almost half, we also decreased the number of SNPs in WE by 25%. This shows that WE represents a rich source of alleles which can be conserved and utilized by selective crossing intended for increasing the frequency of these rare, potentially favorable alleles in breeding populations.

### Genetic Diversity and Differentiation in Flowering Time and Vernalization Genes

In addition to investigating genome-wide statistics, we also estimated genetic differentiation and nucleotide diversity for each SNP. A survey of variation in diversity levels along the genome may identify regions that markedly deviate from the genome average and identify loci showing potential signatures of selection. For example, balancing selection and ongoing selective sweeps locally elevate π, while low values of π occur after a complete sweep or in loci under purifying selection (e.g., housekeeping genes). However, with limited phenotypic and historical information, interpretation of the variation observed in our data would be purely speculative. For this reason, we exclusively focused on growth habit — a trait that was experimentally assessed in this study for all 782 samples — and the major genes involved in flowering time and vernalization pathways, as they are most likely to be associated with natural variation for this particular phenotype. For all three subpopulations, our results show that Fst in the SNPs overlapping or immediately flanking this set of genes was significantly higher than in the rest of the genome. In contrast, the average Π for SNPs in flowering time and vernalization genes was not significantly different from the genome average, except for WA where Π was marginally higher than in the rest of the genome. Significantly higher differences in allele frequencies (measured by Fst) in flowering time and vernalization genes between subpopulations of different growth habits is not surprising. However, because flowering time is fundamental to plant reproduction, we could have expected to find a concomitant signal of purifying selection (low average Π) in these same regions. Similarly, Delourme et al. (2013) did not observe a decrease in diversity around erucic and total glucosinolate QTL, loci known to have undergone strong selection in canola breeding programs. One possible explanation for these perhaps surprising results is that the selection for “double-low” canola cultivars (i.e., devoid of erucic acid and low in glucosinolates) has decreased the overall genome-wide diversity, masking the signal at specific loci such as flowering time genes and oilseed content.

### Putative Chromosomal Inversions

Using per-SNP Fst and Π, we also identified 16 genomic ROI. At least three (and up to seven) of these ROIs showed a distribution of genetic diversity suggestive of a genomic inversion segregating in the subpopulations. Inter-specific inversions seem to be pervasive in the evolution of *Brassica* species (Cai et al., 2014), and evidence suggests that most inversions and other chromosomal rearrangements that differentiate *B. napus* from its parental species occurred immediately after the polyploidization event (Zou et al., 2011). However, to our knowledge, no inversion polymorphism had been described in *B. napus* before.

One characteristic of genomic inversions is that they locally suppress recombination. As a consequence, new mutations are not exchanged between the genomic segments of each orientation. The lack of recombination then increases genetic differentiation and leads to characteristic signatures on PCA plots (e.g., De Jong et al., 2012; Ma and Amos, 2012; Ma et al., 2014; Kemppainen et al., 2015) such as the ones we observed in some ROIs. Natural selection can also create extended haplotypes. However, during a selective sweep, we should observe little or almost no genetic diversity (depending on the speed of the sweep) among the carriers of the selected haplotype, and normal genetic diversity in the rest of the population. The distribution of samples in three discrete bands on the PCA plot is not consistent with this scenario.

Because they suppress recombination, polymorphic inversions can also be detected on high-density genetic maps, as shown on chromosome 1 in maize for example (Rodgers-Melnick et al., 2015). However, this is possible only if the mapping families are derived from parents carrying opposite orientations. In *B. napus*, most recent genetic maps based on high-density SNP markers were constructed using families obtained by crosses of two winter lines (e.g., Delourme et al., 2013; Qian et al., 2014). This would explain why no recombination anomaly was detected on chromosome C02, for example, where nearly all winter types carry the same orientation. Some mapping populations based on crosses between winter and spring types exist, as for example in the study of Hawkins et al. (2002), where the authors detected a possible inversion on linkage group 9 close to the telomere. Similarly, discrepancies in the order of markers shared across different genetic maps also suggest the existence of genomic inversion polymorphisms (e.g., Wang et al., 2011; Raman et al., 2013).

Cytogenetic analyses are needed to confirm or rule out the existence of the putative genomic inversions we identified with SNP markers. Polymorphic inversions are important components of genetic diversity and establishing their precise localization in the genome is highly relevant for plant breeding. For example, alleles located within inverted regions cannot be freely exchanged between cultivars carrying opposite orientations. In addition, SNPs located in large inversions disproportionately contribute to PCA loadings and may bias correction for population structure in genome-wide association studies (Seich al Basatena et al., 2013). More generally, further improving the characterization of the genetic diversity is crucial to achieve an effective management of the gene pool while meeting breeding challenges such as the development of improved cultivars for human consumption, animal feed or feedstocks for biofuel production. The genomic information from our study will support future studies aiming to dissect the genetic basis of complex traits that are important for seed quality (e.g., Wu et al., 2008; Qu et al., 2015) or local adaptation such as leaf cuticular waxes (e.g., Tassone et al., 2016) or drought stress tolerance (Zhang et al., 2015).

## Materials and Methods

### Sample Selection and Growth Habit Assessment

The diversity panel used in this experiment consisted of 843 *B*. *napus* accessions maintained in the USDA-ARS North Central Regional Plant Introduction Station (NCRPIS) in Ames, Iowa (*n* = 564), the Centre for Genetic Resources (CGN) in the Netherlands (*n* = 170), and the University of Idaho (UI) Brassica Breeding Program in Moscow, Idaho (*n* = 109; **Supplementary Table S3**). Using the records maintained by the institutions providing seed for the samples, the accessions, hereafter referred to as samples, were selected to represent the global diversity of *B*. *napus*. As such, the data set included samples from 33 different countries. Growth habit of record for these samples were validated experimentally (see Supplementary Methods).

### Leaf Tissue Collection, DNA Extraction, and Sequence-based-Genotyping

For each of the 843 samples, young leaf tissue was harvested from a single plant for DNA extraction. Sequencing libraries were constructed for Illumina single-end sequencing according to the method described in Truong et al. (2012). Details of DNA extraction and library preparation are provided in Supplementary Methods.

Single-end sequencing (100 nt) was performed using an Illumina HiSeq 2000 (San Diego, CA, USA), where each library of 96 samples was divided over eight lanes. Clusters for each library were generated on a HiSeq flow cell using the TruSeq Single Read Cluster Kit v3, according to manufacturer’s instructions. Following the completion of the run, image analyses, error estimation and base calling were performed using the Illumina Pipeline (HCS 1.5.15.1/RTA v1.13.48 or HCS 2.0.5/RTA v1.17.20) to generate primary data.

### Raw Read Processing and Quality Control

The FASTQ formatted (Cock et al., 2010) primary data were demultiplexed using custom scripts that identified and removed the sample-specific sequencing barcode and the six-nucleotide sequence corresponding to the PstI restriction site (CTGCAG). Each sequence read was evaluated sequentially and retained if it met all of the following criteria: (i) the quality value for the last base of the read was not Illumina code ‘2,’ corresponding to unreliable base-calling values at the end of the read; (ii) the read did not contain any nucleotides encoded as ‘N,’ corresponding to unknown nucleotides; (iii) the read did not contain homopolymers longer than 9 bp; and (iv) the average quality value for the read was greater than or equal to 30, corresponding to an average probability of erroneous base call per nucleotide of less than or equal to 0.001.

### Read Alignment, SNP, and Genotype Calling

The demultiplexed reads were mapped to the reference genome of *B*. *napus* ‘Darmor-*bzh*’ (Chalhoub et al., 2014) using the MEM algorithm (Li, 2013) in BWA version 0.7.8 (Li and Durbin, 2009). Due to the recent divergence of the two diploid progenitor species of *B. napus* (Chalhoub et al., 2014), orthologous regions in the A and C subgenomes of *B*. *napus* have a high sequence identity. As a consequence, sequencing reads tend to have multiple equally probable matches when aligned against the reference genome. To increase alignment sensitivity and reduce potential false positive variants from misalignments, we used custom BWA parameters for seed length (-k 15), SW band width (-w 3), and off-diagonal X-dropoff (-d 5), with all other parameters as default. For each sample, reads with multiple equally probable alignments were eliminated and only reads with a single best match were used for variant detection and genotype calling. A summary of read mapping results are shown in **Supplementary Table S4**. Sequence data for the 782 samples analyzed in this study have been deposited at NCBI under BioProject PRJNA298631 and SRR accessions are listed in **Supplementary Table S3**.

Variant detection and genotype calling were performed using the UnifiedGenotyper (UG) tool in the software GATK version 3.1-1 (McKenna et al., 2010; DePristo et al., 2011). The UG tool was used to generate a variant data set for all the samples (in variant call format, VCF), with default options settings except for the following setting modifications to increase stringency: -mbq 30, -mmq 40, -dcov 200, and –max_alternate_alleles 3.

### SNP and Genotype Filtering

In the following, we refer to a genomic position that is variable in at least one sample as a SNP. We use the term genotype to refer to the alleles at a SNP scored in each sample. The raw vcf file produced by GATK contained 1,081,925 SNPs. Of those SNPs, 112,668 were different from the reference genome but fixed for the alternate allele. Thus, this resulted in 969,257 SNPs that were variable among our samples. Among these 969,257 SNPs, a large proportion (34.9%) of the genotypes had less than 4× coverage (**Supplementary Figure S9A**) and the majority of SNPs had a high proportion of missing genotypes (**Supplementary Figure S9B**). This prompted us to apply a series of stringent quality control filters to the raw SNPs and genotypes to select a subset of high-confidence SNPs suitable for population genetics analysis. Post-processing coverage per sample is given in **Supplementary Table S3**, while the whole filtering pipeline is summarized in **Supplementary Figure S10**. In brief, we filtered out indels and SNPs with more than two alleles. We also removed low confidence genotypes (with less than 4× coverage) and set heterozygous calls with strong allelic imbalance to homozygote for the most frequent allele. Furthermore, on a sample by sample basis, we discarded all genotype calls at a locus when the reads of the sample aligned at that locus produce more than three mismatches compared to the reference genome, as a high density of sample-specific polymorphism per locus is likely the result of alignment errors. Finally, we removed SNPs that produced a genotype call in less than 30 samples. The final data set was composed of 30,881 high-quality SNPs. This final SNP marker data set can be visualized and downloaded from the HRJ project website^4^.

### Sample Filtering and Principal Component Analysis

The filtering pipeline (**Supplementary Figure S10**) involved the elimination of 61 outlier and low quality samples (Step 4, **Supplementary Figure S10**). From the initial 843 samples, 44 with more than 80% missing genotypes across the 42,402 SNPs were removed. An additional 17 samples with a marked discrepancy between their observed location on a PCA plot and their reported growth habit and geographic origin (passport data) were also removed. Further details of the filters applied to samples are provided in Supplementary Methods and **Supplementary Table S5**. Only the remaining 782 samples were used in the analyses, and their geographic origin and growth habit is provided in **Supplementary Table S3**.

To account for missing values in the genotype matrix, the PCA performed in the filtering pipeline, as well as all other PCA in this study, used the probabilistic PCA approach available in the package pcaMethods (Stacklies et al., 2007) in the R software (R Core Team, 2015).

### Neighbor-Joining Analysis

Neighbor-joining trees were constructed using the uncorrected “P” (pairwise) method to compute the distance matrix in SplitsTree4 v4.13.1 (Huson and Bryant, 2006). The distance matrix used to generate the neighbor joining tree was calculated from a weighted genotype matrix that assigned a weight of 1 homozygous genotypes (both for the reference and alternate allele) and 0.5 to heterozygous genotypes. In conducting this analysis, we used 780 of the 782 samples, removing two (1 WAm, 1 SAm) that were extreme outliers in the distribution of the distance matrix values. A total of 1000 bootstrap replicates were run to assess the support of the different nodes.

### Fst

Fst between pairs of subpopulations was estimated using the Hudson estimator for genome-wide data (Hudson et al., 1992) as suggested in Bhatia et al. (2013). Pairwise genetic Fst in the presence of inbreeding was estimated as described in Reich et al. (2009). Calculations were implemented in a custom python script. To ensure Fst estimates were independent of the SNP ascertainment scheme, Fst was calculated twice between each pair of subpopulations, once using SNPs ascertained in one subpopulation, and again using SNPs ascertained in the other subpopulation. Both methods yielded very similar estimates supporting that our Fst estimates were robust to alternative ascertainment schemes. We also calculated Fst for each SNP for the same pairs of subpopulations and plotted these values along the genome.

### Site Frequency Spectrum

We generated a SFS for each subpopulation and each subgenome by probabilistically downsampling each data set to a sample size of 100 chromosomes (50 diploid samples) according to the method described in Marth et al. (2004). Implementation of the downsampling scheme allowed us to compare the SFS of WE, WA, and SP despite their different sample sizes. In addition, it served as a method to analyze the same effective number of genotypes sampled per SNP, avoiding distortions due to an unequal amount of missing data across sites. Because we did not know the ancestral state of each allele, we used the folded SFS, where the counts corresponded to the allele with lowest number of copies (minor allele).

### Nucleotide Diversity and Tajima’s D

We used the downsampled SFS to estimate both the genome-wide average nucleotide diversity, π (Nei and Li, 1979) and the deviation of the allele frequency distribution compared to a neutral model of evolution (where the neutral model serves as a null hypothesis) with Tajima’s *D* statistics (Tajima, 1989). Calculating π using the SFS downsampled to 100 chromosomes effectively sets the sample size to 50 samples across all SNPs considered, independent of the amount of missing data at each site. Hence, the average number of pairwise mismatches per site can be normalized by the same sequence length across all samples. The sequence length for the downsampled SFS was estimated by multiplying the number of sites in the downsampled SFS by the SNP density (i.e., the number of SNPs over the number of base pairs in the reference genome covered by the reads containing these SNPs) estimated on all the SNPs before downsampling. The SNP density was estimated for each subgenome separately. In addition, we calculated the average number of pairwise mismatches at each SNP (i.e., ignoring invariable positions), and called this quantity Π. For one SNP, Π is the number of nucleotide differences between all pairs of genotypes at this SNP divided by the number of comparisons (2*n* choose 2, for n diploid samples). Both quantities, Π and π, are related by π = Π/*L* where *L* is the length of the sequence queried. For one SNP, (*L* = 1), Π equals π.

### Gene Annotation and Localization

To annotate SNPs as genic (within a gene) or intergenic (not within a gene), we used the *B. napus* genome annotation version 5 (Brassica_napus.annotation_v5.onchr.gff) generated by (Chalhoub et al., 2014) and downloaded from CoGE^5^.

To examine whether the evolutionary history of the three major subpopulations carries the hallmark of local adaptation, we studied genes associated with variation in growth habit. We used the genomic coordinates for the nine *FLC* copies provided in Chalhoub et al. (2014). In addition, we used the list of major genes regulating flowering time and vernalization described in Schiessl et al. (2014). For these genes, genomic coordinates were obtained by using the translated *Arabidopsis thaliana* amino acid sequence to find orthologs in the *B. napus* nucleotide genome sequence with the TBLASTN function of the BLAST^+^ program (Camacho et al., 2009). The functional description of the *A. thaliana* best match (protein sequences with the highest similarity between *B. napus* and *A. thaliana*) was used as a proxy for the *B. napus* gene function. The genomic coordinates of all the flowering time and vernalization gene copies used in this analysis are provided in **Supplementary Table S6**, and further details of the BLAST procedure are provided in Supplementary Methods.

### Permutation Tests

We tested whether the 117 flowering time and vernalization genes identified above were associated with elevated Fst or low average number of pairwise differences (Π) more often than expected by chance. To estimate the values of Π and Fst for each of the 117 genes, we used 40 SNPs representative of each gene. This allowed estimates to be less sensitive to local sample call rate and SNP density. For each gene, these representative SNPs included all the SNPs within the gene (if any) plus as many closest flanking SNPs on each side of the gene as needed for a total of 40 SNPs. For each gene, we calculated an average of Π and Fst over the 40 representative SNPs, and averaged these values over the 117 genes (4680 SNPs total). We compared these statistics against a subset of 4680 SNPs randomly chosen across the genome. To ensure an unbiased comparison, we selected these random SNPs to have similar characteristics as the set of SNPs representative of flowering time and vernalization genes. The SNP set of the A genome was composed of 821 intergenic and 1499 genic SNPs and the set of the C genome was composed of 1087 intergenic and 1273 genic SNPs. We repeated the random selection of SNPs 1,000 times, and for each iteration, we calculated an average of Π and Fst across sampled SNPs. The *P*-value is the number of times out of 1000 the summary statistics (Π or Fst) for flowering time and vernalization genes was greater than the one observed for the subset of randomly selected SNPs. This test was implemented with a custom python script.

## Author Contributions

EG, ET, DI, and MG prepared the manuscript. EG, ET, and DI processed the sequencing data; performed the bioinformatics and population genetic analyses. MW, JBD, and JB provided germplasm management, collection and analyzed growth habit data. ED performed the preliminary bioinformatic analysis of SBG data. HW managed the experimental design and SBG data production. DG developed a genome browser to visualize the genomic data. DG, JMD, MJ, JB, and MG provided experimental design and coordination.

## Disclaimer

Mention of trade names or commercial products in this publication is solely for the purpose of providing specific information and does not imply recommendation or endorsement by the U.S. Department of Agriculture. USDA is an equal opportunity provider and employer. The SBG technology is protected by patents and patent applications owned by Keygene N.V.

## Conflict of Interest Statement

The authors declare that the research was conducted in the absence of any commercial or financial relationships that could be construed as a potential conflict of interest.

## Footnotes

1 http://apps.fas.usda.gov/psdonline/

2 http://apps.fas.usda.gov/psdonline/psdHome.aspx; Table 03: Major Vegetable Oils: World Supply and Distribution (Commodity View)

3 http://apps.fas.usda.gov/psdonline/psdHome.aspx; Table 02: Major Protein Meals: World Supply and Distribution (Commodity View)

4 https://hrj.usda.iastate.edu/gb2/gbrowse/b_napus/

5 https://genomevolution.org/coge/
