# Supplementary material for: Population Genomic Analysis Reveals Differential Evolutionary Histories and Patterns of Diversity across Subgenomes and Subpopulations of Brassica napus L
Source: Front Plant Sci. 2016 Apr 21;7:525. doi: 10.3389/fpls.2016.00525 (PMC4838616; doi:10.3389/fpls.2016.00525)
Supplement: Supplementary file 7 [file Data_Sheet_1.DOCX]

Supplementary Material

Population genomic analysis reveals differential evolutionary histories and patterns of diversity across subgenomes and subpopulations of *Brassica napus* L.

**Elodie Gazave^*^, Erica E. Tassone, Daniel C. Ilut, Megan Wingerson, Erwin Datema, Hanneke M. A. Witsenboer, James B. Davis, David Grant, John M. Dyer, Matthew A. Jenks, Jack Brown, and Michael A. Gore^*^**

*** Correspondence:** eeg47@cornell.edu; mag87@cornell.edu

**1 Supplementary Methods**

***Experimental growth habit validation***

To validate the growth habit of record for these samples, two seeds from each sample were planted in 3.8 x 6.2 cm cells in 48-cell trays on greenhouse benches in two independent replicates. Four spring and five winter cultivars from the UI collection whose growth habit had previously been assessed in the UI breeding program were also included as checks. Plants were grown under optimal conditions (sodium halide supplemental lighting to provide a 16-hour day length, daytime temperature maintained between 20 and 23°C with a minimum night temperature of 13°C, watered daily and fertilized once weekly). The samples that flowered in the greenhouse without vernalization were classified as "spring" types. Samples that had not flowered by seven weeks after planting were classified as "winter" types and were placed into a walk-in growth chamber for vernalization (constant day-night temperature of 4°C with a 12-hour day length for 15 weeks). After the vernalization period, these plants were transplanted back into the greenhouse under the aforementioned growing conditions.

***Sequencing libraries preparation***

Leaf discs of approximately 1 cm in diameter were collected from the harvested leaf tissue and frozen at -20°C, followed by lyophilization for 48 h at 50x10^-3^ mBar with a collector temperature of -50°C. The freeze-dried tissue samples were shipped to Keygene N.V. (Wageningen, NL) where total genomic DNA was isolated from ground leaf material using a modified CTAB procedure (Stewart and Via, 1993). To ensure that the isolated total genomic DNA samples were neither contaminated nor degraded, 100 ng of each sample was run on a 1% agarose gel along with 500 ng of two λ HindIII size/mass standards. Sequencing libraries were constructed for Illumina single-end sequencing according to the method described in Truong et al. (2012). Briefly, 100-500 ng of total genomic DNA was digested using 5 units PstI and 5 units MseI for at least 1 h at 37°C. Following digestion, the mixture was heated at 85°C for 10 min. Adapter ligation was then performed using a universal P7 MseI adapter (5′-CAAGCAGAAGACGGCATACGAG-3′; 5′-TACTCGTATGCCGTCTTCTGCTTG-3′-NH2) and a sample-specific tagged PstI P5 adapter (5′-AATGATACGGCGACCACCGAGATCTACACTCTTTCCCTACACGACGCTCTTCCGATCTxxxxxATGCA-3′; 5′-TxxxxxAGATCGGAAGAGCGTCGT-3′-NH2, where xxxxx represents the sample identification tag) for 3 h at 37°C. Sample-specific PstI P5 adapters contained a unique five nucleotide (nt) sample identification tag adjacent to the PstI restriction site overhang for identification of individual samples. They were designed such that each sample identification tag was unique on each plate and differed by at least two bases from all other tags. PCR was performed in a total reaction volume of 20 µl containing 5 µl of 10-fold diluted restriction-ligation mixture, 5 ng Illumina P5 primer (5′-AATGATACGGCGACCACCG-3′), 30 ng Illumina P7 primer (5′-CAAGCAGAAGACGGCATACGA-3′), 0.2 mM dNTPs, 0.4 U AmpliTaq (Applied Biosystems, Foster City, CA, USA) and 16 U Ampli-Taq buffer. PCR was performed with a cycle profile that consisted of 2 min at 72°C, 13 cycles of 30 s at 94°C, 2 min at 67°C and 2 min at 72°C in which the annealing temperature after each cycle was lowered by 0.7°C, followed by 37 cycles of 30 s at 94°C, 2 min at 58°C and 2 min at 72°C. Reactions were held at 4°C until ready for use. Next, sets of 96 PCR amplified samples were pooled (5 µl each) to make 8 libraries. For each library, fragments were separated, sized and quantified using the Agilent High Sensitivity DNA Kit (Agilent Technologies, Santa Clara, CA, USA) and loaded onto the Agilent 2100 Bioanalyzer for evaluation.

***SNP and genotype calling***

In an effort to generate a high-quality set of SNPs, we changed the default parameters of the UnifiedGenotyper in GATK. Options -mbq and -mmq represent the minimum base quality required to consider a base for calling, and the minimum read mapping quality required to consider a read for calling, respectively. The chosen value in the present study (30) is equivalent to a 0.001 probability of being incorrect. Stronger stringency would eliminate too many reads, and lower stringency would allow reads that are either not reliably mapped or reads of lower quality. Option -dcov (target coverage threshold for downsampling to coverage) was set to 200, allowing to keep only 200 reads in regions of excessive coverage. This is because having additional reads is not informative and imposes a major computational cost. The option --max_alternate_alleles indicates how many alleles will be output (no matter how many are found at a given position). This option was set to three in the present study. For a pair of chromosomes, at a single polymorphic positions, there are a most four possible nucleotides (A,C,T,G). Two alleles is the most common scenario, while three or four alleles would occur in the very rare case of recurrent mutations, and more likely indicate alignment errors. Because the present study was not concerned with indels (that may have more than two alleles, depending on the length of the indel), and because we focused on biallelic SNPs to allow population genetics statistics that are designed for dealing with two alleles, setting the option to any value above two did not influence our results.

We also chose not to use other options available in GATK. Specifically, MarkDuplicate works poorly for short single-end reads, and does not apply to restriction-based genome subsampling such as RadTAG or SBG since the algorithm expects distinct start locations for each genuine read. Base recalibration is designed to treats every reference mismatch as indicative of machine error. To perform this recalibration, GATK uses a reference set of known SNPs to skip over most polymorphic sites and not recalibrate them as a potential machine error. Since most SNPs in our study were de novo discovered, we consider this step unnecessary and potentially misleading. IndelRealigner works on the same principle, and a user needs to input a list of known indels to the IndelRealigner algorithm, and such a data set was not available. It is in theory possible to run the UnifiedGenotyper once in a purely discovery phase, extract the indels found in this run to create a list of known indels for the IndelRaligner. Once reads around indels have been realigned, it is then necessary to re-call all the SNP with a new iteration of the UnifiedGenotyper. Given the computational burden associated with such a procedure on our 842 initial sample, we considered it more efficient to apply stringent quality control filters to the non-realigned UnifiedGenotyper output. In particular, we filtered out sites with clustered SNPs (as they typically occur around indels) during the quality control steps applied to the vcf and before data analysis, as described on Suppl. Figure S10 and in the methods of the main manuscript.

***Sample classification and filtering***

We conducted a PCA on the genotype matrix to examine the genetic structure among samples and identify potential outliers (Suppl. Figure S12). This PCA involved all 843 samples and the subset of 42,402 SNPs with less than 90% missing genotypes across samples (*n* = 29,947). We initially labeled the samples according to their geographic origin (as recorded in the germplasm repository they were obtained from; see Materials and Methods) and their growth habit (determined experimentally in this study). Samples that did not cluster as expected on the PCA (i.e. with the majority of samples of the same growth habit and geographic origin) were labeled as putative outliers and treated as follows. First, we examined the sample call rate of the outlier samples (i.e. the number of genotyped SNPs in each sample) and observed that PCA outliers were not among the samples with the highest proportion of missing genotypes (Suppl. Figure S13). However, to be conservative, we further tightened the filtering criteria and removed 44 samples with a sample call rate lower than 20%. Second, we cross-validated geographic origin and growth habit using the cultivar name of 71 outlier samples to query additional germplasm repository databases (the Japanese Tsukuba database, http://xs-tbr.tsukuba-sogotokku.jp/browse?value=Brassica+napus+L.&type=subject and the Czech Evigez database http://genbank.vurv.cz/genetic/resources/asp2/form_1_a.asp ). For 54 of these 71 outlier samples, the genetic information (indicated by the location of the sample on PCA) was supported by one or both of the additional databases prompting for a sample reclassification (Suppl. Table S5). For 17 of the 71 samples, no additional information was found and as such the outlier sample was removed from the data set. In total, the filtering procedure removed 61 (44+17) samples. As a final validation of the reclassification procedure, we conducted a PCA within each subpopulation separately (i.e. within SP, WA, or WE alone) and verified that each was homogeneous.

***Flowering time and vernalization gene mapping***

In their study, Schiessl et al. (2014) mapped flowering time and vernalization genes onto specific *B. napus* chromosomes, but did not provide exact genomic locations within each chromosome. To obtain the genomic coordinates for these genes, we used the translated *A. thaliana* amino acid sequence to find orthologs in the *B. napus* nucleotide genome sequence using the TBLASTN function of the BLAST+ program. Because Schiessl et al. (2014) used an experimental procedure (sequence capture array) to identify copies of these genes in *B. napus*, we considered their list as a gold standard, and filtered the TBLASTN output based on their results. Therefore, among all the local alignments with E-value less than or equal to 5e-11, we kept those with the lowest E-values that were located on the same chromosomes as described in Schiessl et al. (2014). We recovered 102 of the 131 copies described by the authors on chromosomes other than "unknown." Of the 29 copies not recovered, we ignored one copy of *SUF4* reported on chromosome C02 that yielded an E-value of 4e-5. One copy of *FUL* and one copy of *CAL* matched exactly the same genomic coordinates in the TBLASTN output, and we kept only the copy of *FUL* as it had the lowest E-value (3e-30). The other 26 copies either did not yield TBLASTN results on the correct chromosome, or generated a match on one of the chromosomes annotated as "random" in the current genome assembly. In an additional six cases, TBLASN generated an alignment on a different chromosome compared to the one described in Schiessl et al. (2014), but with an even lower E-value than the best match on the correct chromosome. We retained these six copies as putative copies of flowering time genes not captured in the Schiessl et al. (2014) experiment, and annotated them as “new.”

**2 Supplementary Figures and Tables**

**2.1 Supplementary figure legends**

**Suppl. Figure S1: Distribution of non-missing genotypes in the final data set.** (A) For each of the 30,881 SNPs in the final data set (x-axis), the y-axis shows the proportion of samples that were successfully genotyped (SNP call rate). (B) For each of the 782 samples in the final dataset (x-axis), the y-axis represents the proportion of SNPs successfully genotyped (sample call rate).

**Suppl. Figure S2: Loadings of principal component 1 along the genome.** Each dot represents a SNP for which the contribution to principal component 1 (PC1, loading) is represented on the y-axis. SNPs are ordered according to their genomic location (x-axis), with the different chromosomes represented by alternating colors. Overall, SNPs with very positive or negative loadings are present on several chromosomes of the genome. However, there are two particular regions (on chromosome C01 and C02) where these SNPs appear in clusters, indicating genomic regions that have a strong contribution to the genetic variance among samples on PC1.

**Suppl. Figure S3: Principal component analysis of the *B. napus* diversity panel of 782 samples for each subgenome separately.** (A) A subgenome alone, (B) C subgenome alone. The first two principal components (PCs) are represented. For both subgenomes, the principal component analysis (PCA) reveals three clusters as observed on the PCA plot of the whole genome (Figure 2), although the variance explained by PC1 is smaller for the A subgenome compared to the C subgenome. Samples are labeled according to their growth habit and geographic origin.

**Suppl. Figure S4: Distribution of the number of sequencing reads in the 782 samples.** (A) The percentage of genotyped SNPs per sample (sample call rate, x-axis) slightly increases with the number of reads (y-axis, represented on a log scale). However, both the number of reads (B) and sample call rate (c) have largely overlapping distributions between spring (SP), winter Asia (WA), winter America (WAm) and winter Europe (WE) samples. This indicates that the reference genome used for read alignment does not appear to have markedly biased the sample call rate in any one particular subpopulation. Therefore, the subpopulation differences we reported in various analyses of genetic diversity are unlikely to have been caused by ascertainment bias. In panels (A) and (B), the read counts only include the sequencing reads that were used for SNP calling (i.e. reads that passed quality control filters and uniquely aligned to the reference genome). For four samples with technical replicates, read counts are averaged across replicates.

**Suppl. Figure S5: Folded site frequency spectrum of the C subgenome for the three major subpopulations after filtering two regions of high differentiation.** The x-axis represents in log scale the number of copies of the minor allele observed for a SNP (minor allele count, MAC) in the subpopulation. The y-axis represents in log scale the proportion of SNPs in the subpopulation for all MAC. The black dots represent the SFS prior to filtering, while other colors represent the SFS after filtering two regions of extreme differentiation in the SP subpopulation. The two regions filtered are a 9.1 Mb region on chromosome C02 and a 6.5 Mb region on chromosome C07. The coordinates of these regions are given in Suppl. Table S2, and represented on Figure 1 as ROI 2 and ROI 6, respectively. The shape of the SFS is only mildly affected by the filtering. All 3 subpopulations have been probabilistically downsampled to 50 diploid samples to make their sample size fully comparable (see Materials and Methods).

**Suppl. Figure S6: Per-SNP Fst in the three major subpopulations.** The x-axis represents the physical position (in bp) on a chromosome and the y-axis represents the Fst values for each SNP calculated for each pair of subpopulations (winter Asia (WA) vs. spring (SP), WA vs. winter Europe (WE), and WE vs. SP). The gray lines represent the per-SNP Fst values averaged over 40-SNP sliding windows, and the colored lines represent the lowess smoothing of these values. The regions of interest (ROI) presented in Figure 1 are represented by gray boxes (see Suppl. Table S2 for genomic coordinates). Circles at the top of the figures represent the location of flowering time and vernalization genes on each chromosome. The different symbols (open circle="new", crossed circle="Chalhoub et al.", filled circle="Schiessl et al.") correspond to the three different resources used to map these genes (see Materials and Methods and Suppl. Methods). Genomic coordinates of flowering time and vernalization genes are provided in Suppl. Table S6.

**Suppl. Figure S7: Average number of pairwise mismatches in the three major subpopulations.** The x-axis represents the physical position (in bp) on a chromosome and the y-axis represents the average number of pairwise mismatches (Π) for each SNP and in the winter Europe (WE), winter Asia (WA), and spring (SP) subpopulations. The gray lines represent the values of Π averaged over 100-SNP sliding windows, and the color lines represent the lowess smoothing of these values. The regions of interest (ROI) presented in Figure 1 are represented by gray boxes (see Suppl. Table S2 for genomic coordinates). Circles at the top of the figures represent the location of flowering time and vernalization genes on each chromosome. The different symbols (open circle="new", crossed circle="Chalhoub et al.", filled circle="Schiessl et al.") correspond to the three different resources used to map these genes (see Materials and Methods and Suppl. Methods). Genomic coordinates of flowering time and vernalization genes are provided in Suppl. Table S6.

**Suppl. Figure S8: Principal component analysis of the 16 regions of interest.** For each region of interest (ROI), the PCA was performed using all 782 samples, but only with the subset of SNPs located within the genomic coordinates of the ROI. These coordinates are given in Suppl. Table S2. Samples are labeled according to their growth habit and geographic origin.

**Suppl. Figure S9: Distribution of coverage and SNP call rate in the 969,257 SNPs previous to filtering.** (A) The percentage of genotypes (y-axis) for different depth of coverage (x-axis) is shown across all SNPs successfully genotyped. Genotypes with less than 4X coverage (marked with the vertical blue dashed line) represent 34.9% of the genotypes, and were removed in step 1 of the filtering pipeline. (B) The y-axis shows the cumulative distribution of SNPs when the SNP call rate (percentage of samples for which each SNP was successfully) increases. The red line represents the median of the distribution, the orange and purple bars represent the 25th and 75th quantiles, respectively. The SNPs with a call rate inferior or equal to 6.4% represent 25% of all SNPs (yellow dotted line). In 50% (red dotted line) and 75% (purple dotted line) of the SNPs, the SNP call rate is at most 29.3% and 56%, respectively.

**Suppl. Figure S10: Overview of the bioinformatics pipeline used to filter the SNP data set.** From the 969,257 SNPs (i.e. positions with at least one copy of the alternate allele) in the vcf file, we removed indels and SNPs with more than 2 alleles. Genotypes with less than 4X coverage were scored as missing. Heterozygous (htz) genotypes that had a high allelic imbalance (more than 70% of one allele and less than 30% of the other allele) were scored as homozygous (hmz) for the majority allele. These filters removed 608,326 low confidence SNPs, which resulted in a data set of 361,001 SNPs (Step 1). We next eliminated SNPs with less than 30 genotypes across all the samples. This step imposed a very stringent filter due to the high missing rate and resulted in 85,103 SNPs (Step 2). To be conservative, we also removed potential false positive SNPs that may have resulted from alignment errors. Potential alignment errors were defined as SBG loci with more than three mismatches per read and/or alignments shorter than the read length (clipped reads). This procedure (Step 3) generated a list of 42,402 high-confidence SNPs. Step 4 involved a PCA of the 42,402 SNPs that led us to filter out 61 of the 843 samples (see sections "Sample filtering and principal component analysis" in Methods and "Sample classification and filtering" in Suppl. Methods). Of the remaining 39,685 SNPs, we removed those that did not have a genotype call for at least 10 samples in each of the three major subpopulations defined by PCA (Step 5). The high-quality subset of 30,881 SNPs obtained at the end of this pipeline constituted our final data set. Clear and hatched boxes represent SNP and genotype filtering steps, respectively.

**Suppl. Figure S11: Per-SNP proportion of heterozygous samples in the three major subpopulations.** The x-axis represents the physical genomic position (in bp) on a chromosome and the y-axis represents the proportion of heterozygous samples (heterozygosity) for each SNP and in each of the three major subpopulations: winter Europe (WE), winter Asia (WA), and spring (SP). The gray lines represent the per-SNP heterozygosity, and the colored lines represent the lowess smoothing of these values. The regions of interest (ROI) presented in Figure 1 are represented by gray boxes (see Suppl. Table S2 for genomic coordinates). Circles at the top of the figures represent the location of flowering time and vernalization genes on each chromosome. The different symbols (open circle="new", crossed circle="Chalhoub et al.", filled circle="Schiessl et al.") correspond to the three different resources used to map these genes (see Materials and Methods and Suppl. Methods). Genomic coordinates of flowering time and vernalization genes are provided in Suppl. Table S6.

**Suppl. Figure S12: Principal component analysis of all 843 *B*. *napus* samples prior to sample filtering.** The first two principal components (PCs) are represented. Only SNPs with a call rate greater than 10% were used for the principal component analysis (PCA). Samples are labeled according to their growth habit and geographic origin. Although three distinct clusters are evident, some samples appear to be misclassified such that they appear genetically more similar to those of a different cluster. For these samples, growth habit and geographic origin were verified in additional germplasm repository databases.

**Suppl. Figure S13: Principal component analysis of all 843 *B*. *napus* samples classified according to their sample call rate.** The first two principal components (PCs) are represented. Samples with 80% or more missing genotypes (sample call rate less than 20%) do not appear as outliers compared to other samples of their same growth habit and geographic origin. Conversely, these samples cluster toward the center of the principal component analysis (PCA) plot, reflecting the lack of information they carry (rather than erroneous information).

**2.2 Supplementary tables**

**Supplementary Table 1.** Observed and expected distribution of SNPs per subgenome.

**Supplementary Table 2.** Genomic coordinates for regions of interest (ROI).

**Supplementary Table 3.** List of accessions used in this study.

**Supplementary Table 4.** Read alignment summary statistics.

**Supplementary Table 5.** Criteria applied during the manual curation of sample growth habit and geographic origin.

**Supplementary Table 6.** Genomic coordinates of flowering time and vernalization genes.

**3 Supplementary references**

Schiessl, S., Samans, B., Hüttel, B., Reinhard, R., and Snowdon, R. J. (2014). Capturing sequence variation among flowering-time regulatory gene homologs in the allopolyploid crop species *Brassica napus*. Front. Plant Sci. 5, 404. doi:10.3389/fpls.2014.00404.

Stewart, C. N., and Via, L. E. (1993). A rapid CTAB DNA isolation technique useful for RAPD fingerprinting and other PCR applications. BioTechniques 14, 748–750.

Truong, H. T., Ramos, A. M., Yalcin, F., de Ruiter, M., van der Poel, H. J. A., Huvenaars, K. H. J., et al. (2012). Sequence-Based Genotyping for Marker Discovery and Co-Dominant Scoring in Germplasm and Populations. PLoS ONE 7, e37565. doi:10.1371/journal.pone.0037565.
