# Supplementary figures and images for: Population Genomic Analysis Reveals Differential Evolutionary Histories and Patterns of Diversity across Subgenomes and Subpopulations of Brassica napus L
Source: Front Plant Sci. 2016 Apr 21;7:525. doi: 10.3389/fpls.2016.00525 (PMC4838616; doi:10.3389/fpls.2016.00525)

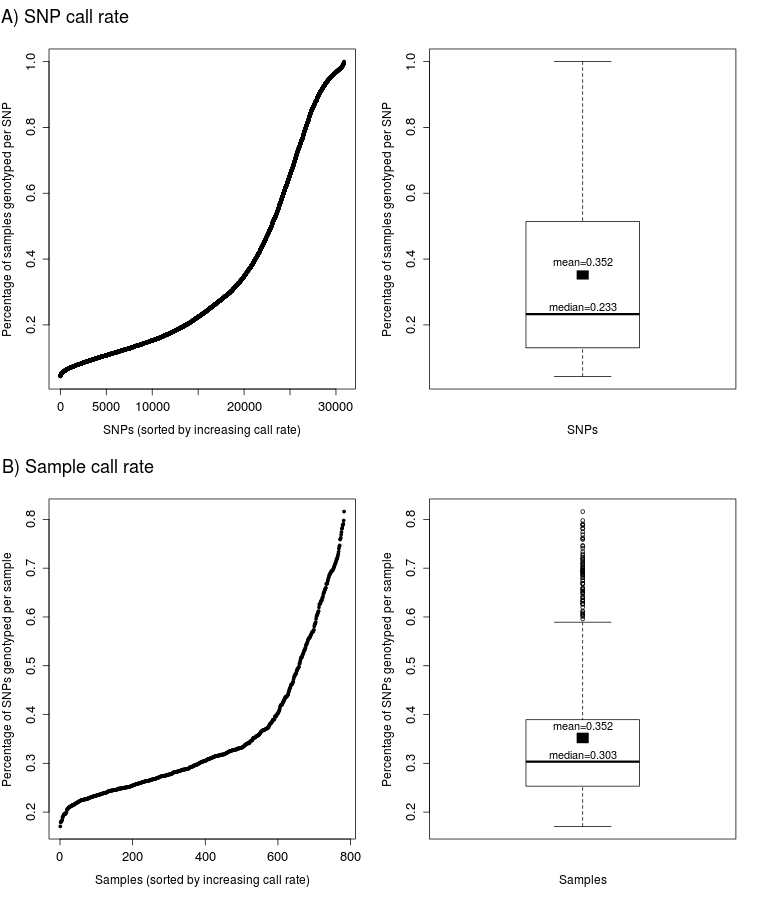

Supplement: Supplementary file 8 [file Image_1.TIFF]

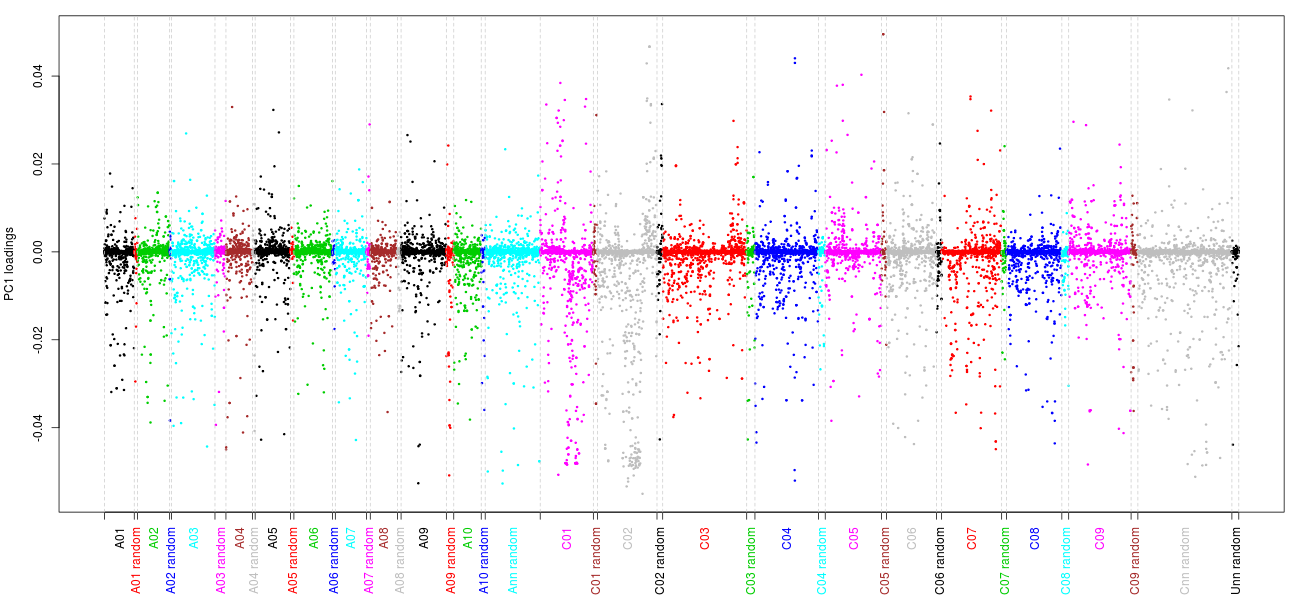

Supplement: Supplementary file 9 [file Image_2.TIFF]

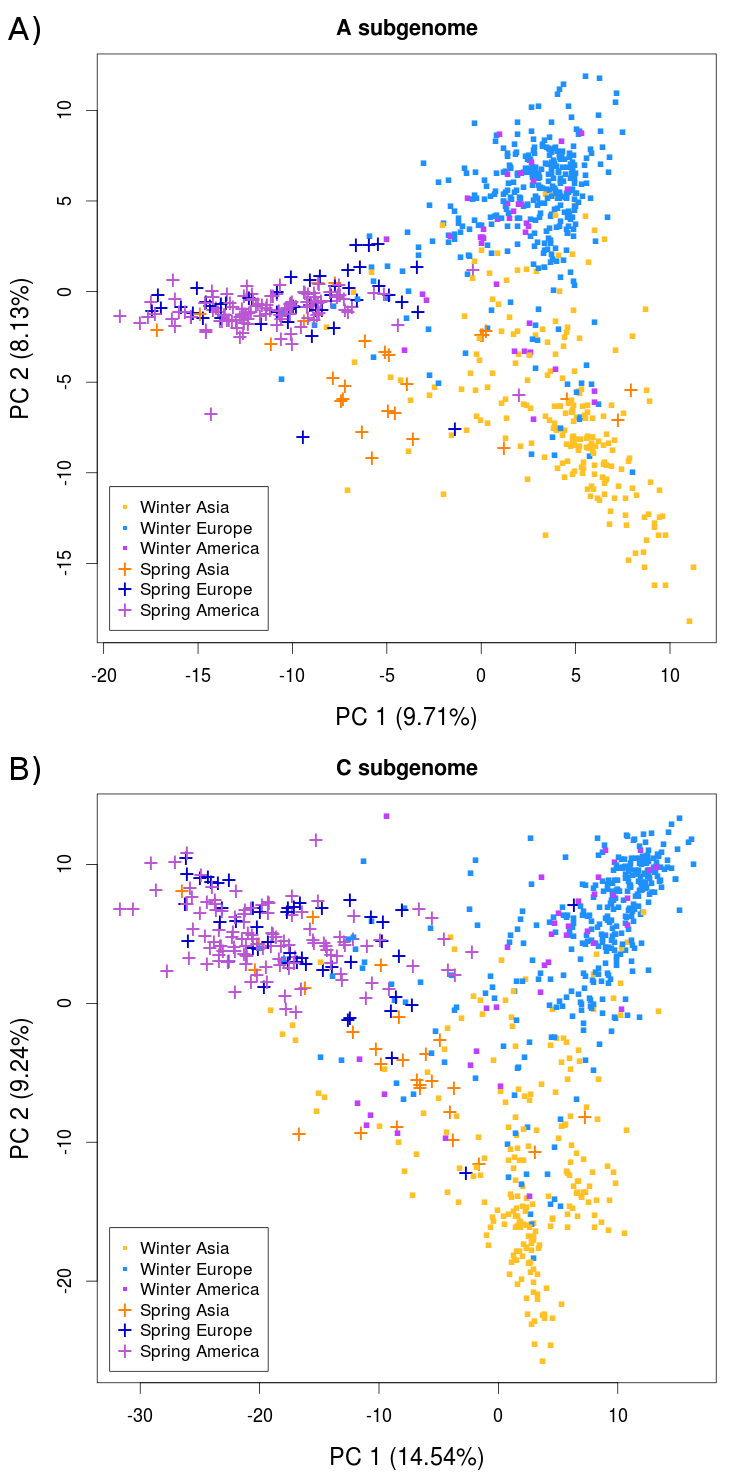

Supplement: Supplementary file 10 [file Image_3.TIFF]

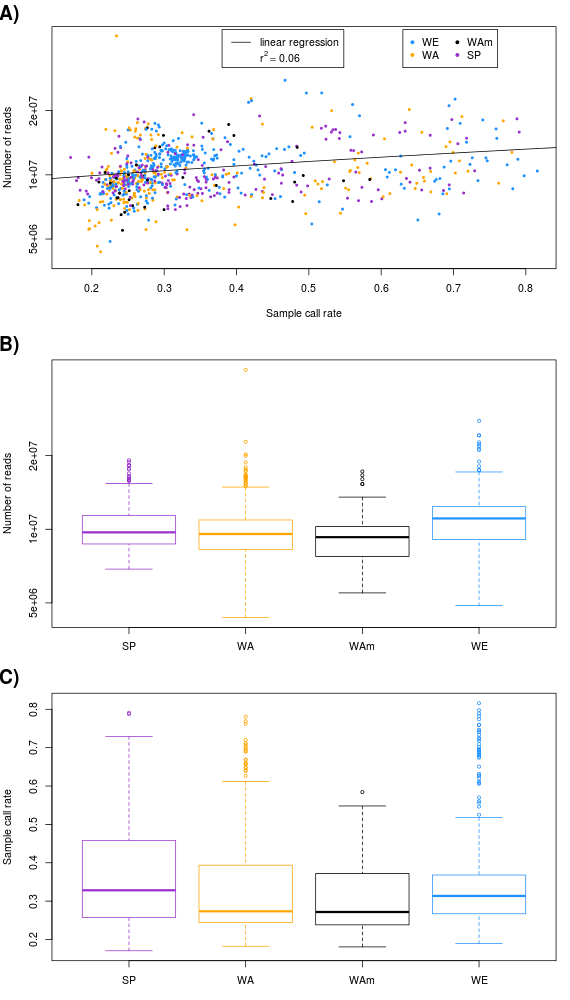

Supplement: Supplementary file 11 [file Image_4.TIFF]

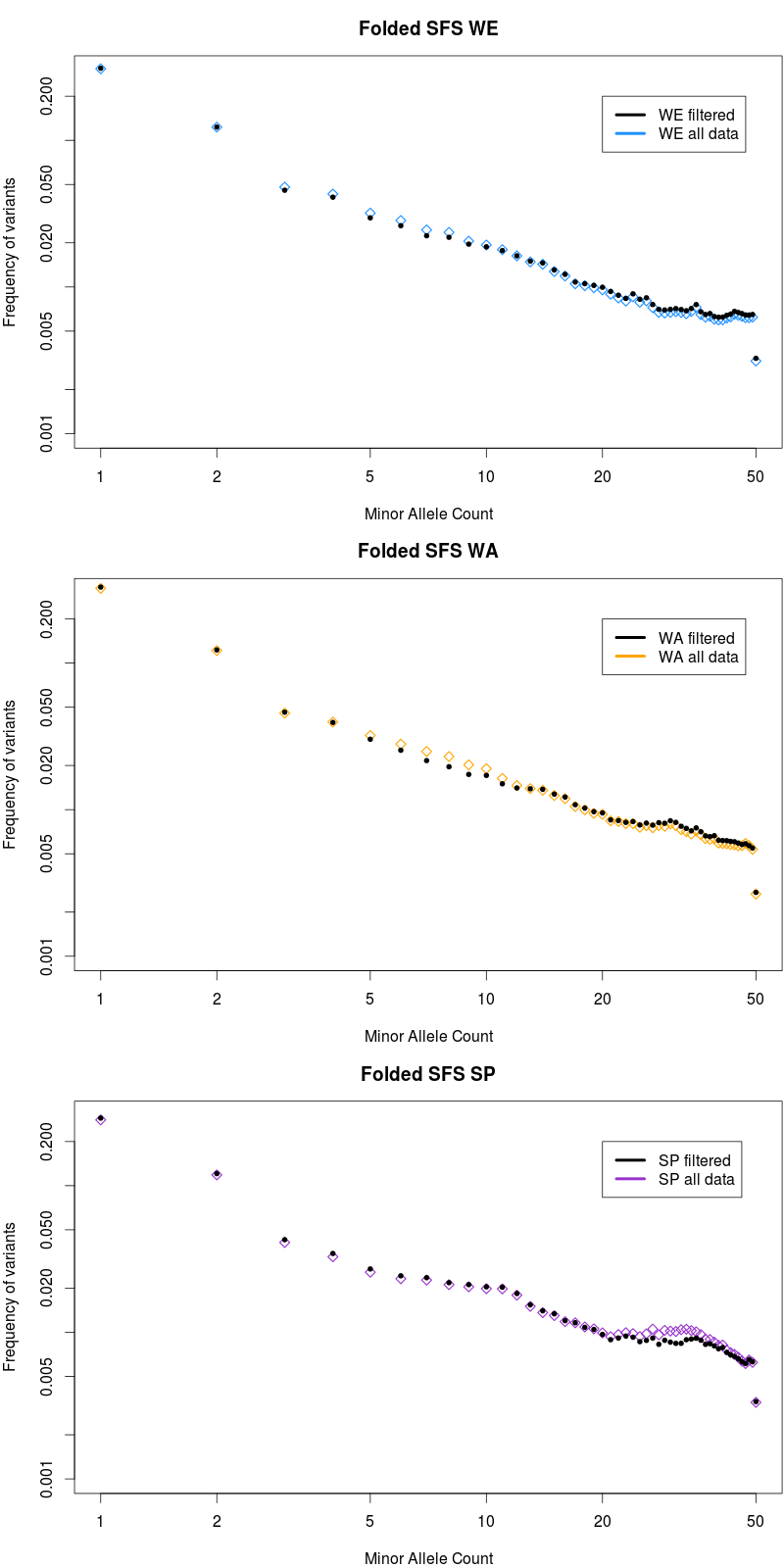

Supplement: Supplementary file 12 [file Image_5.TIFF]

ROI 1

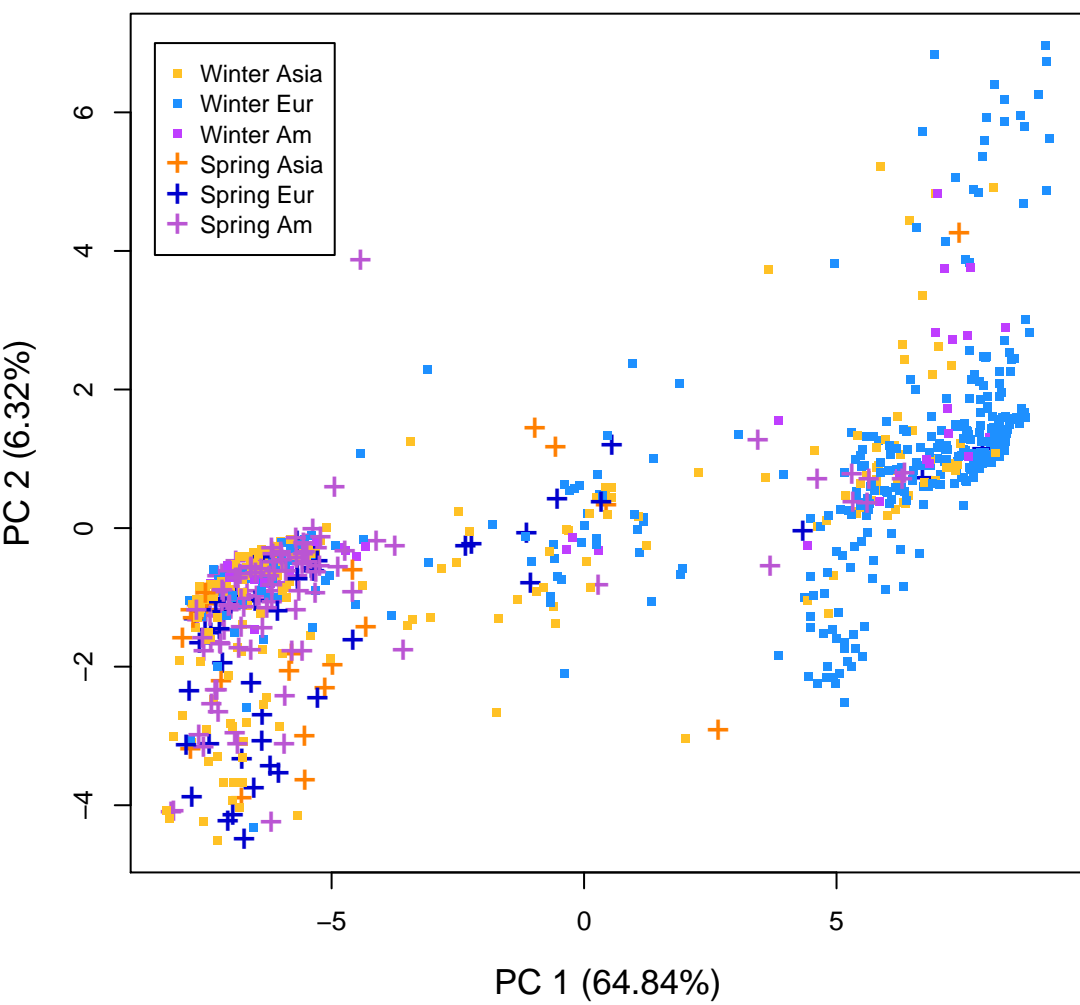

ROI 2

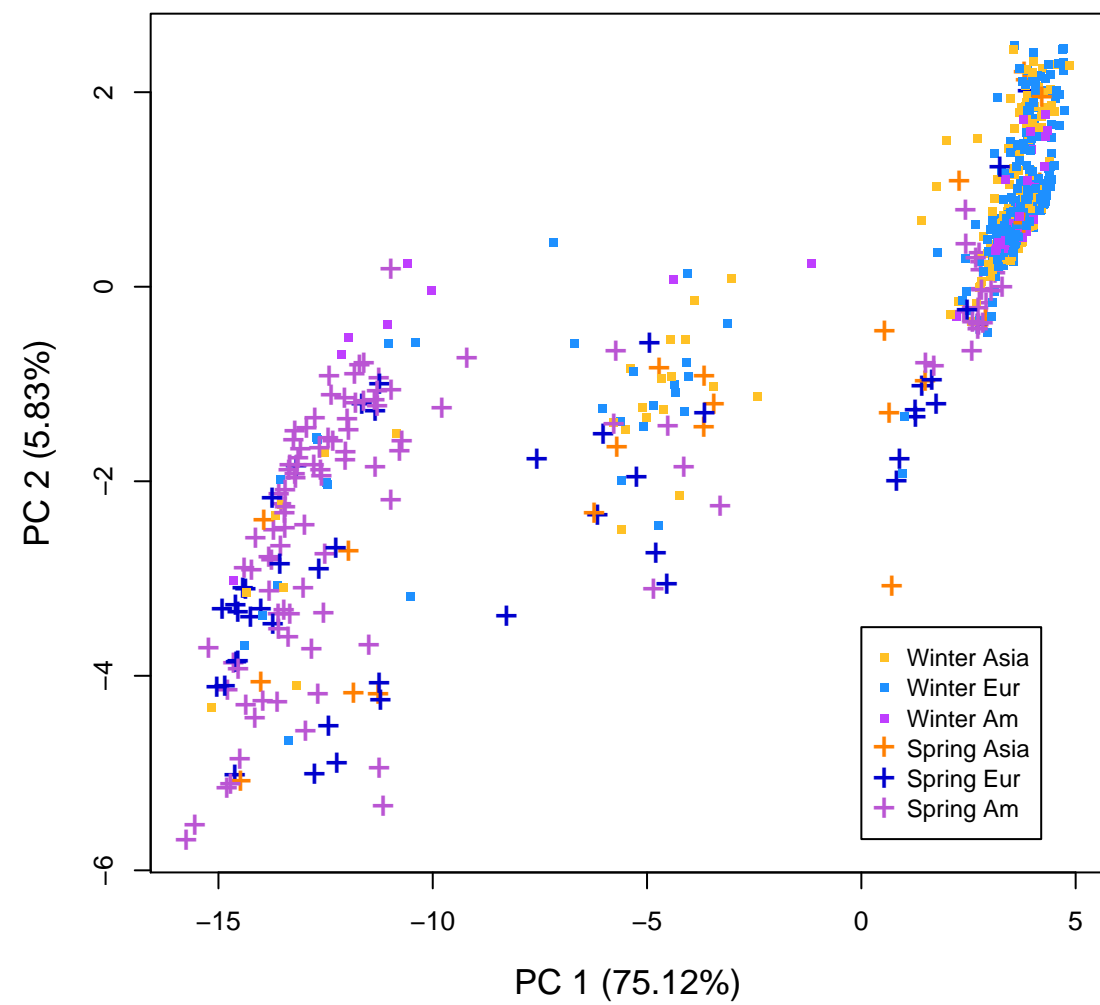

ROI 3

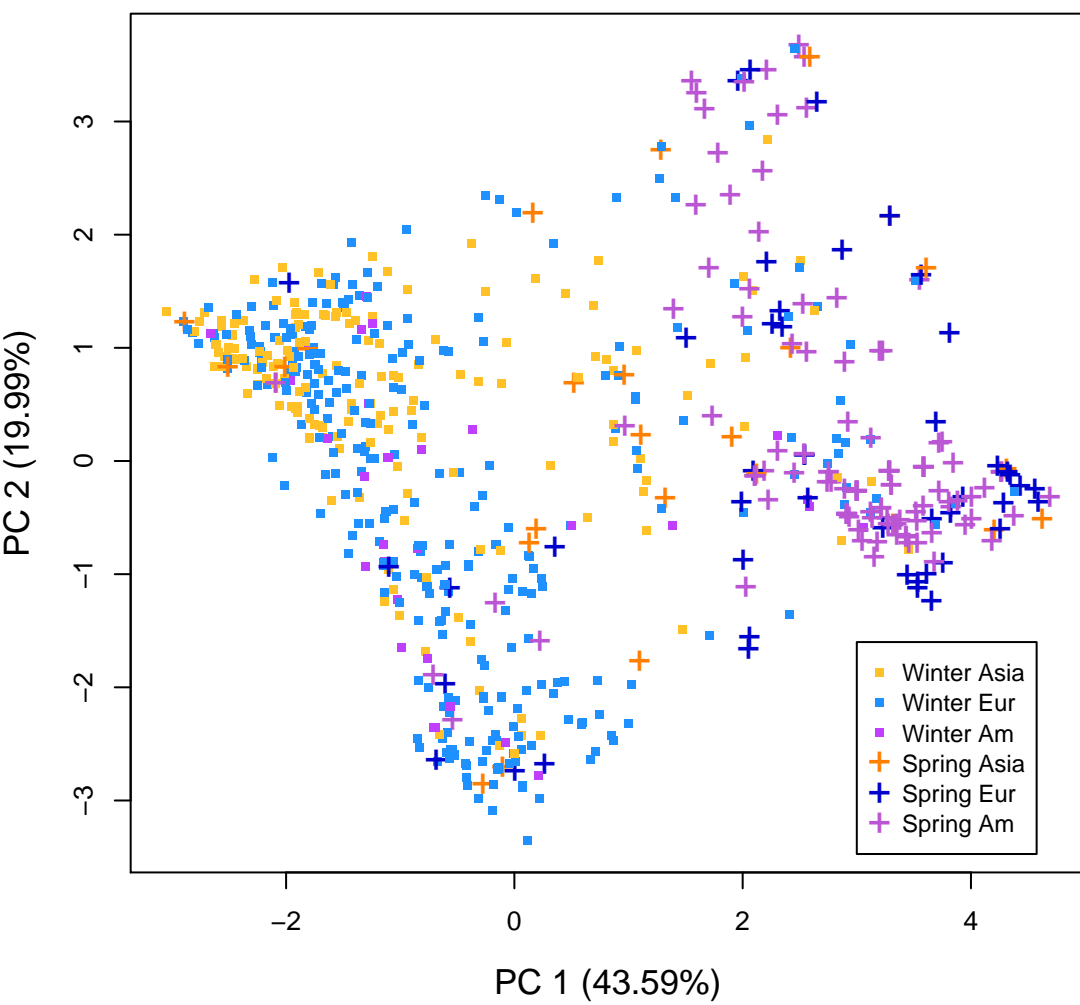

ROI 4

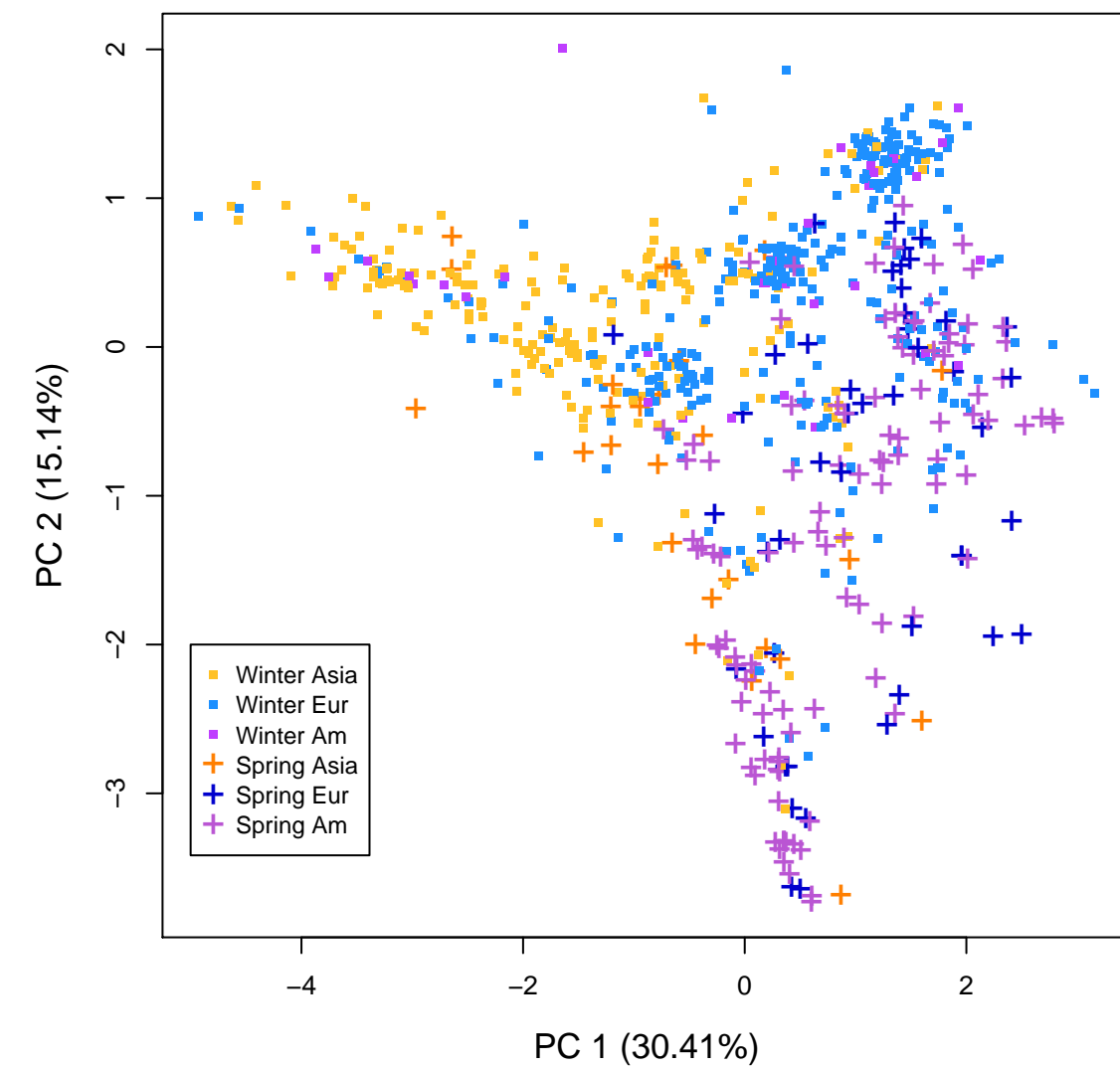

ROI 5

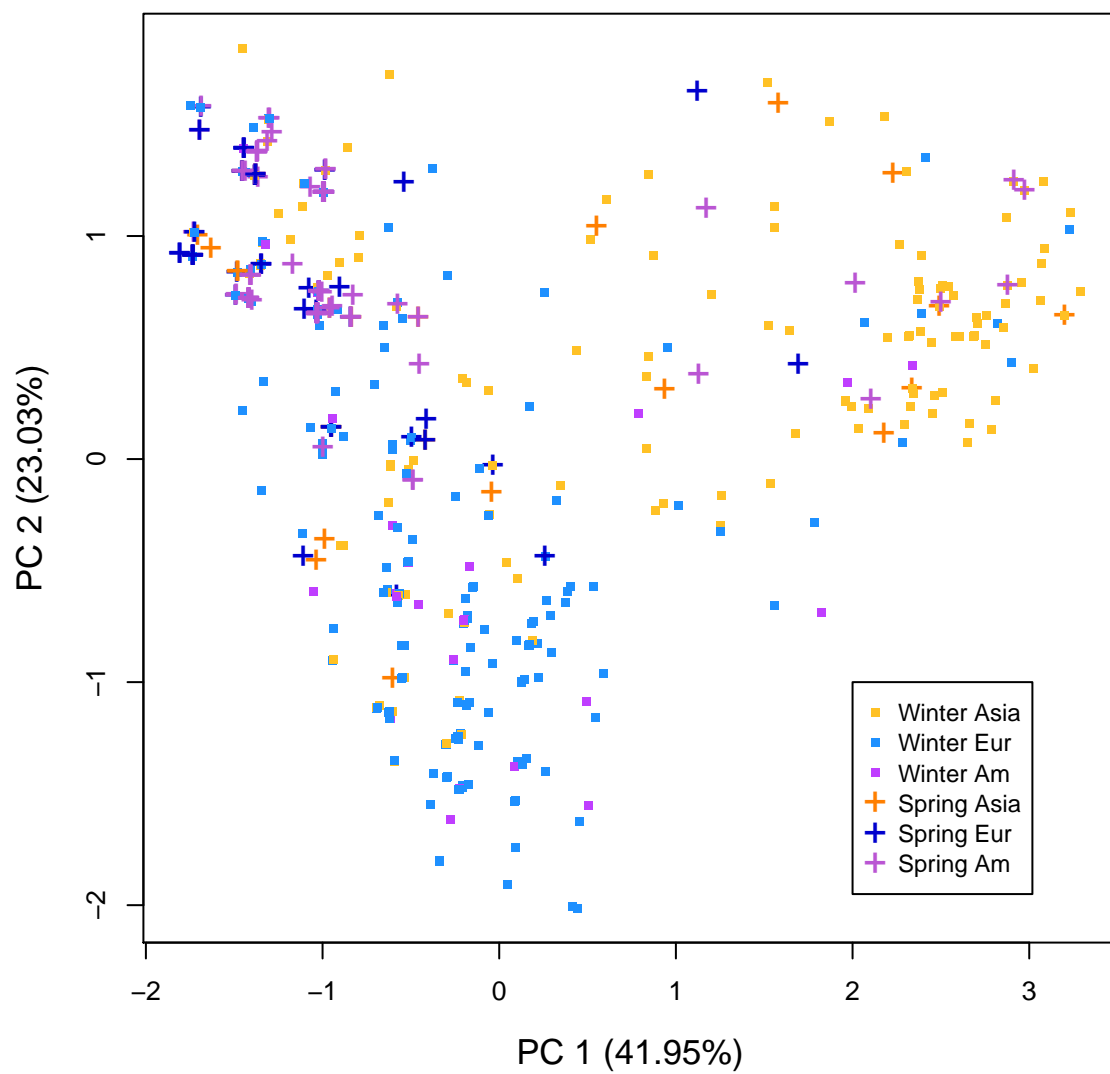

ROI 6

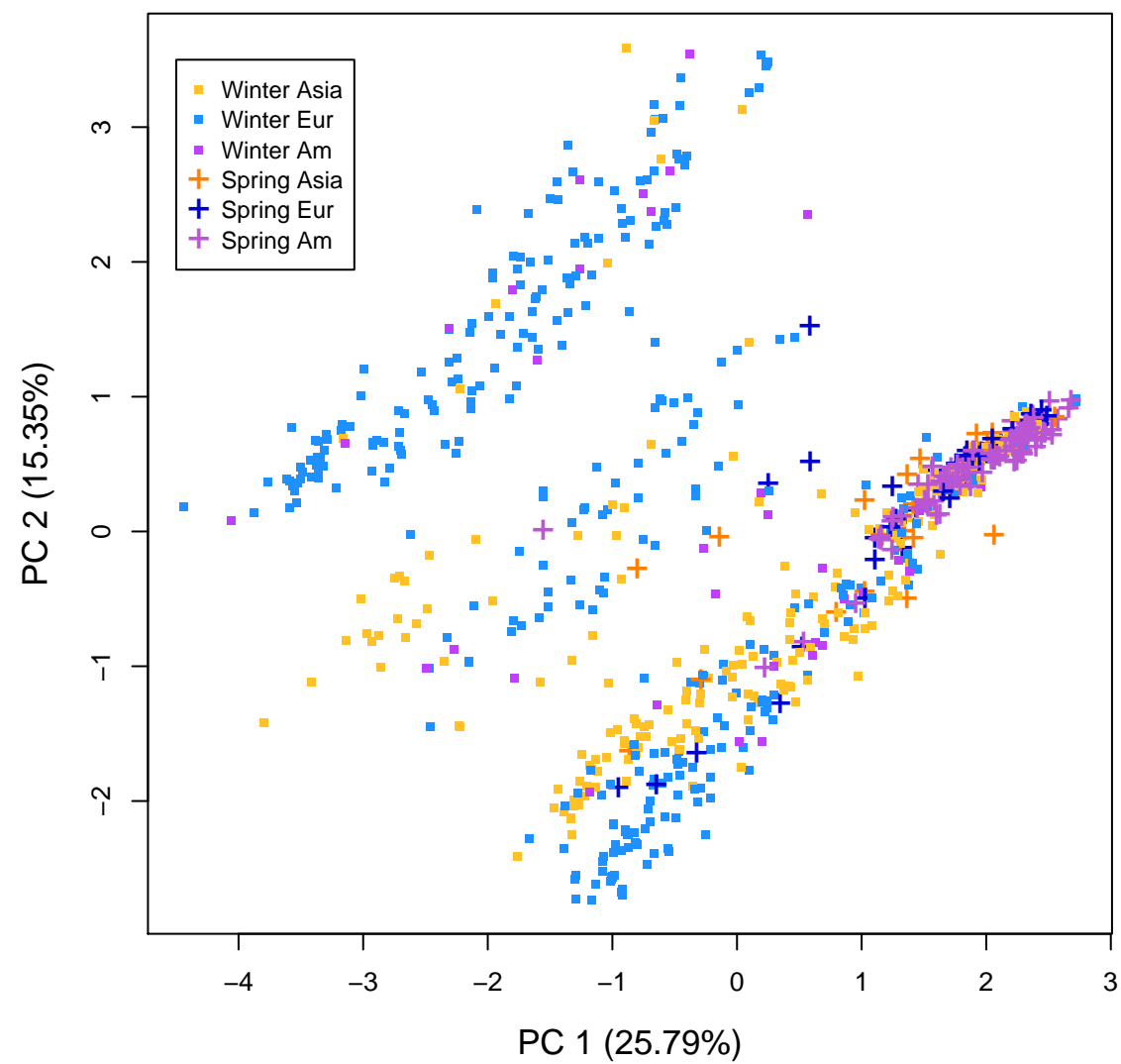

ROI 7

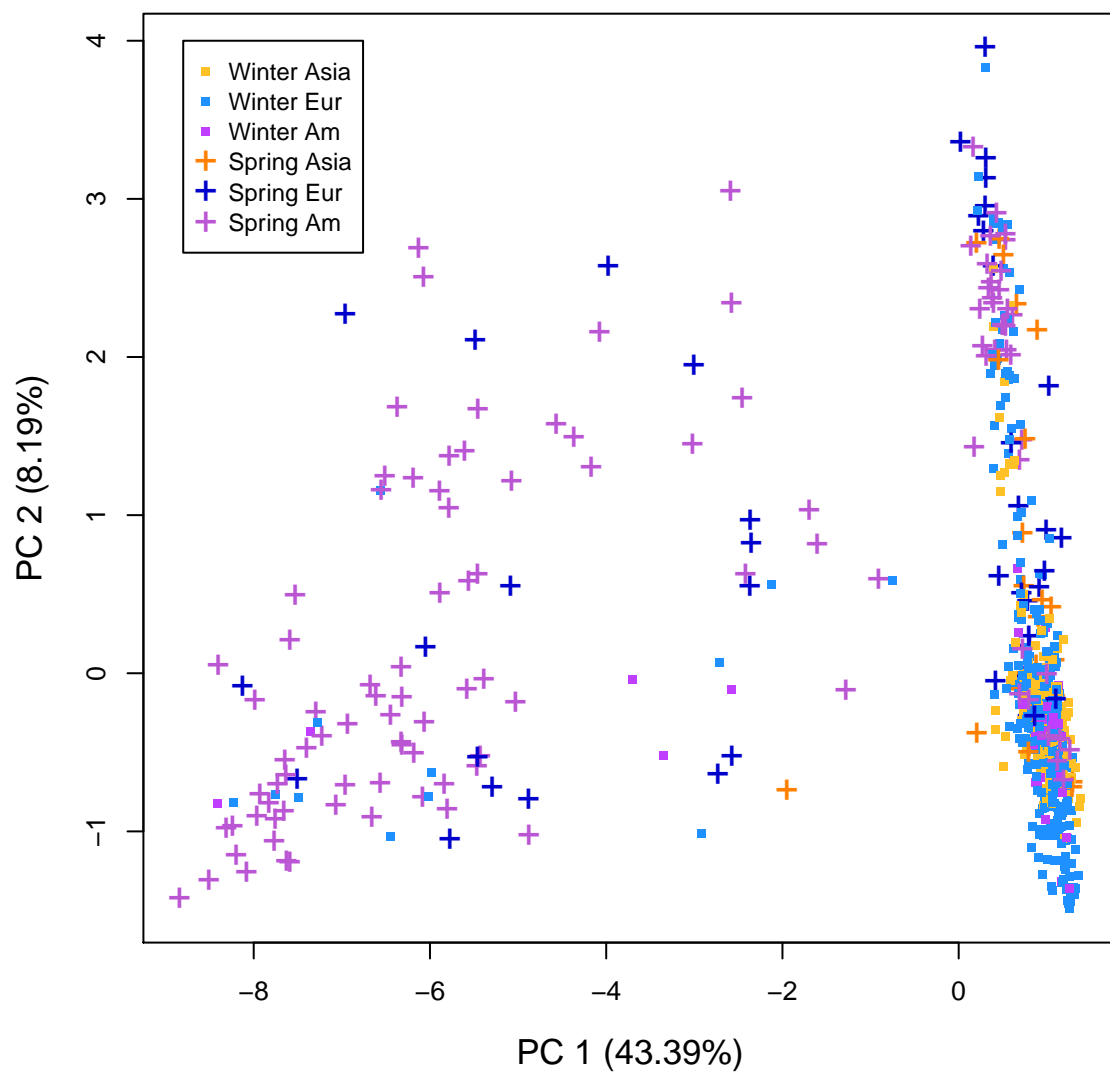

ROI 8

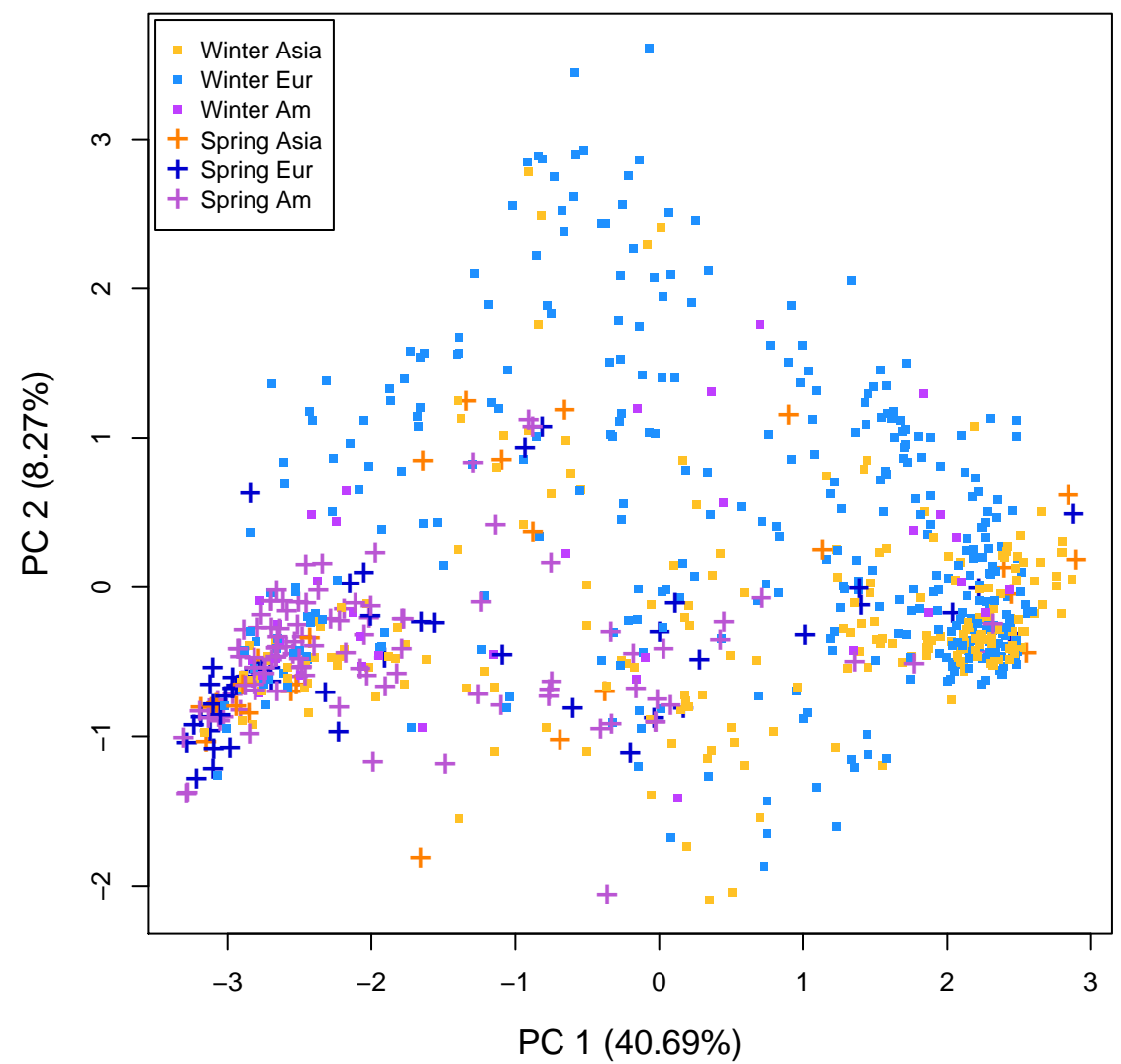

ROI 9

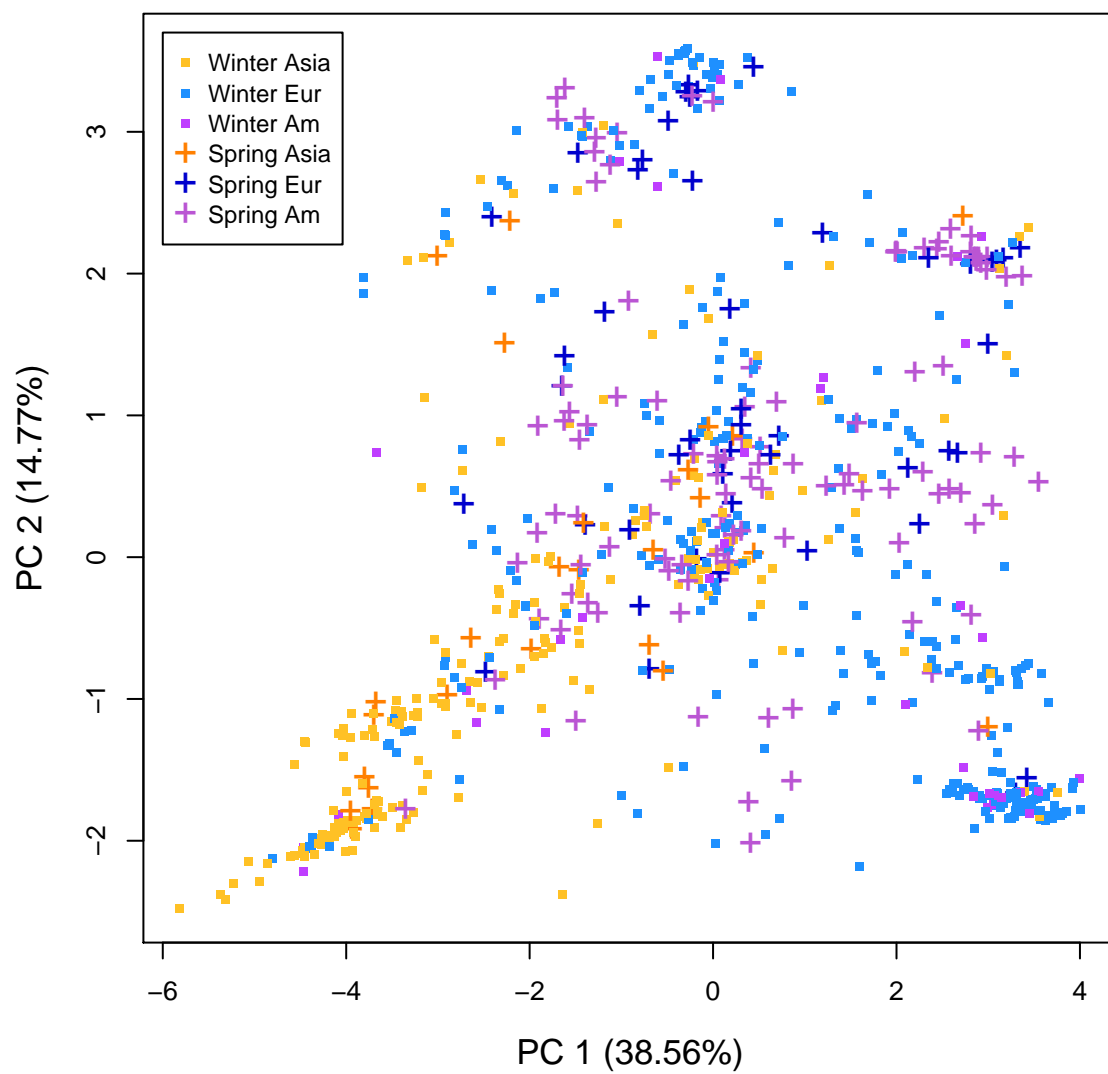

ROI 10

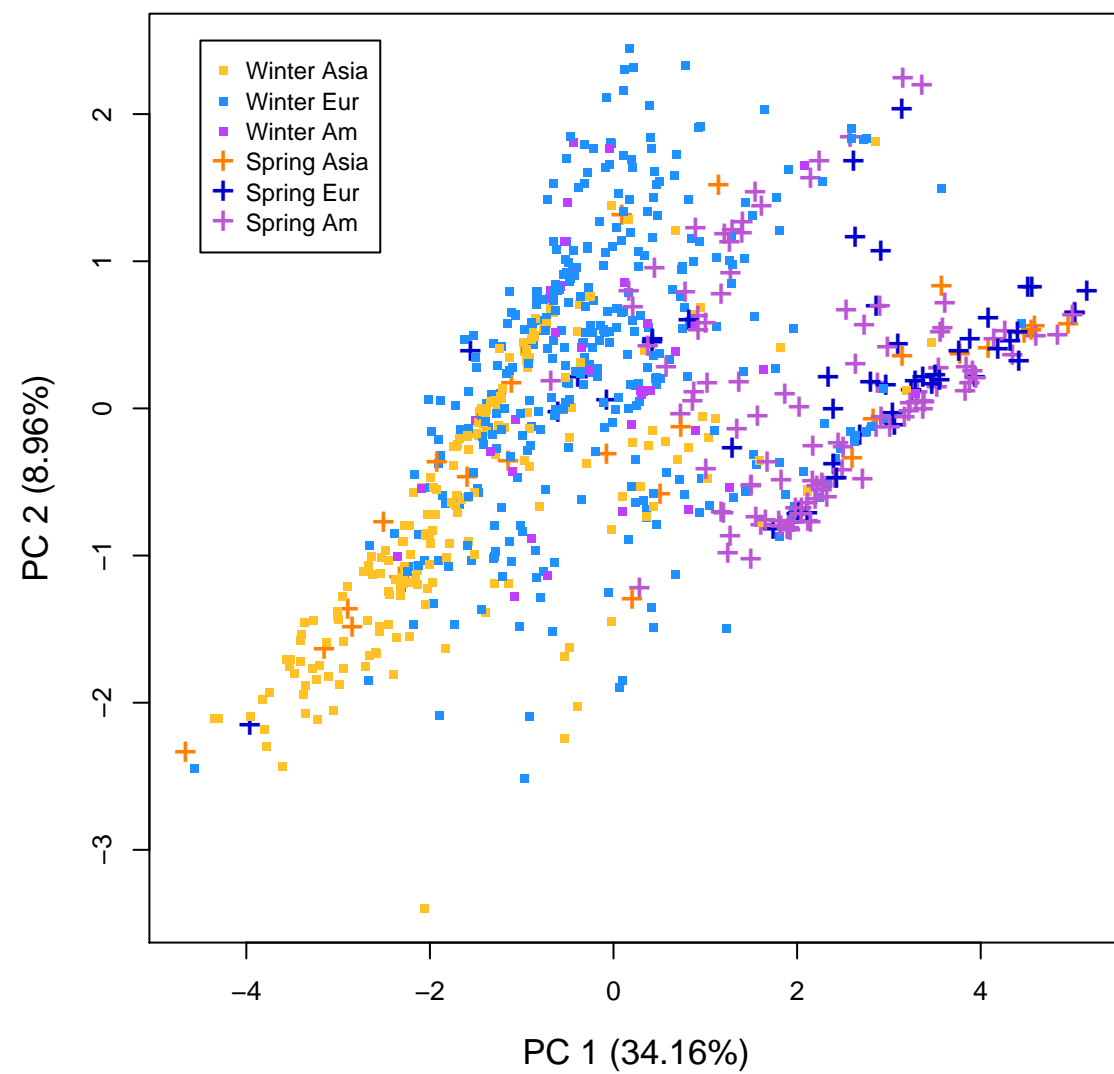

ROI 11

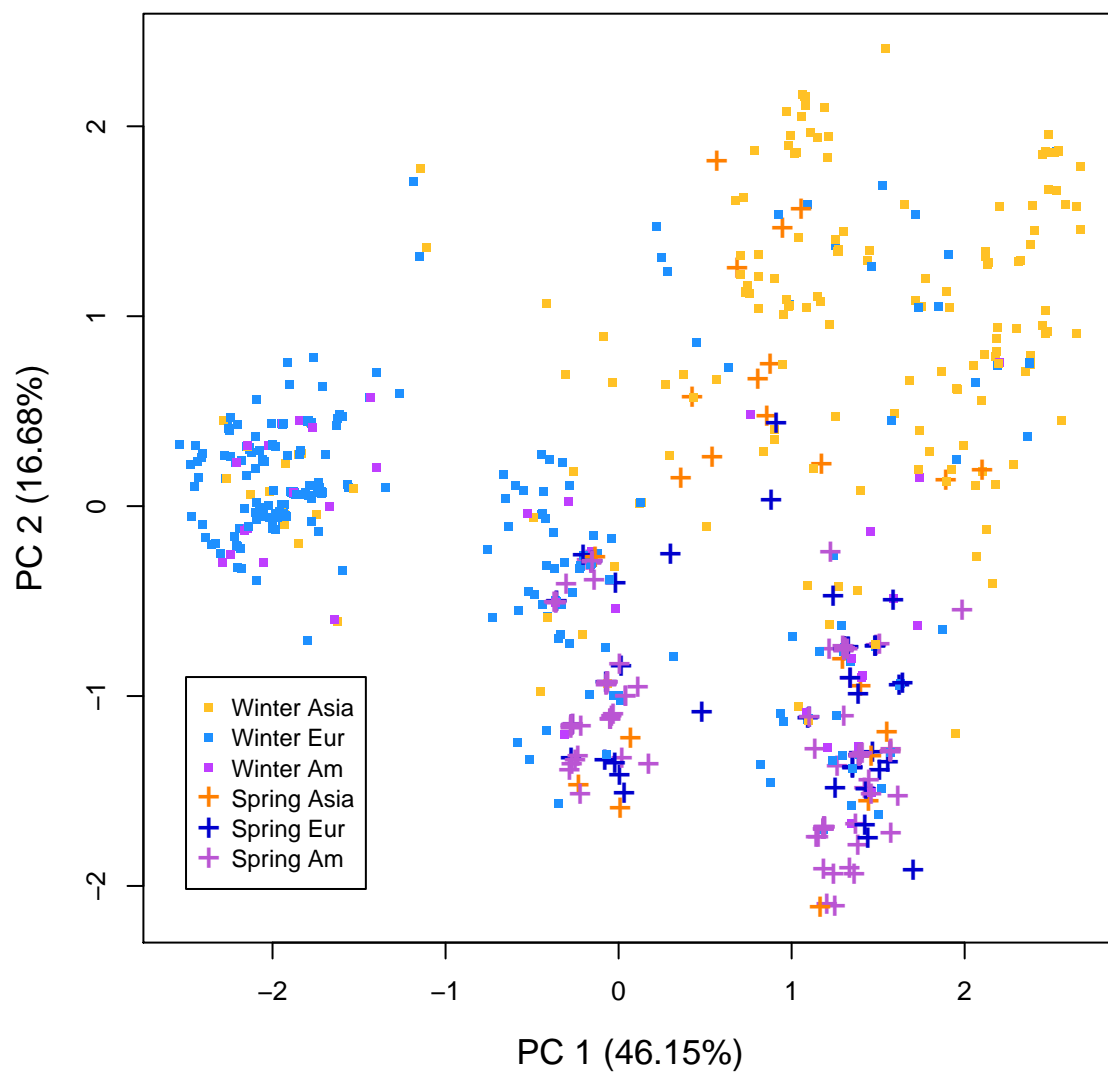

ROI 12

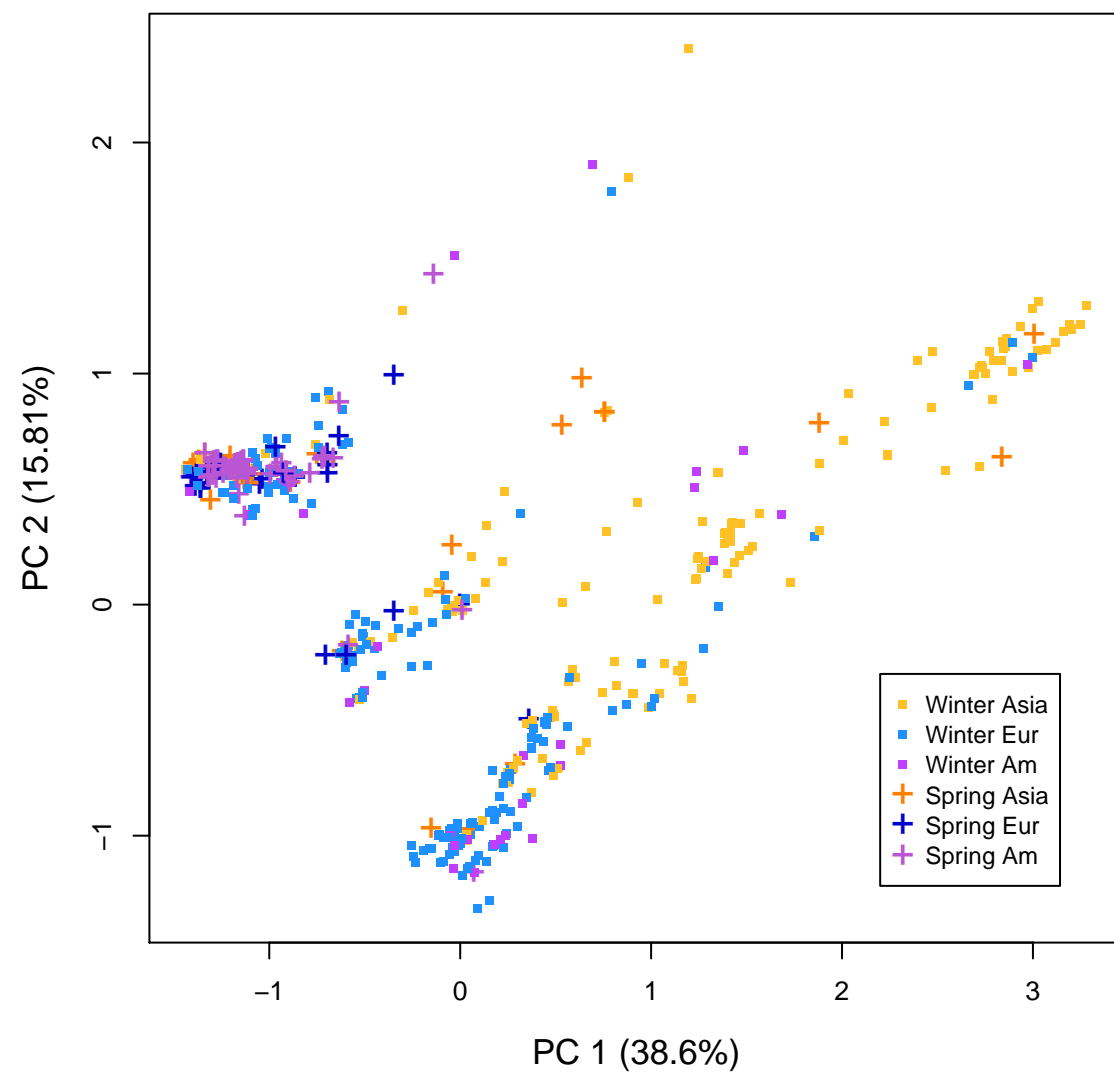

ROI 13

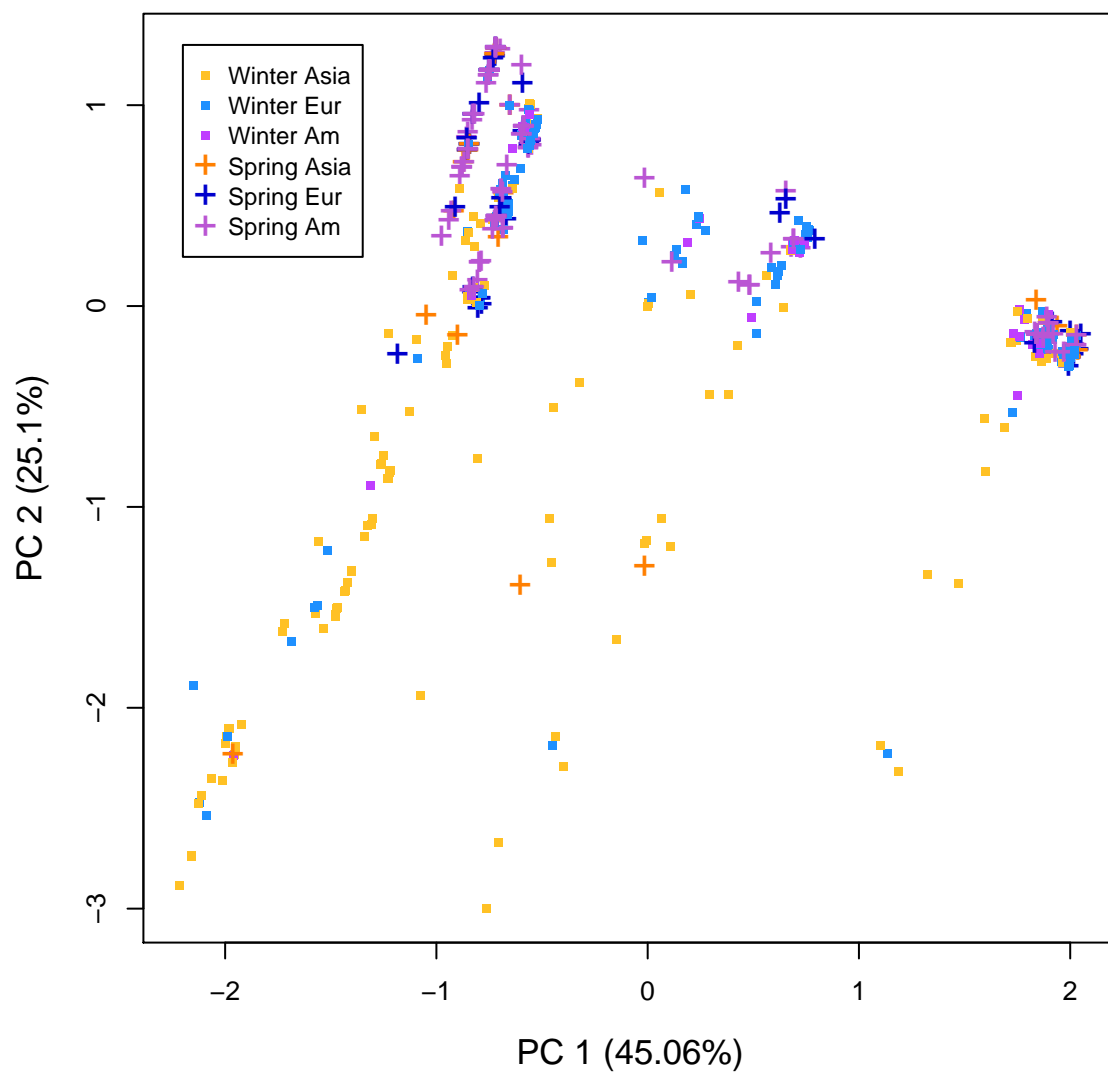

ROI 14

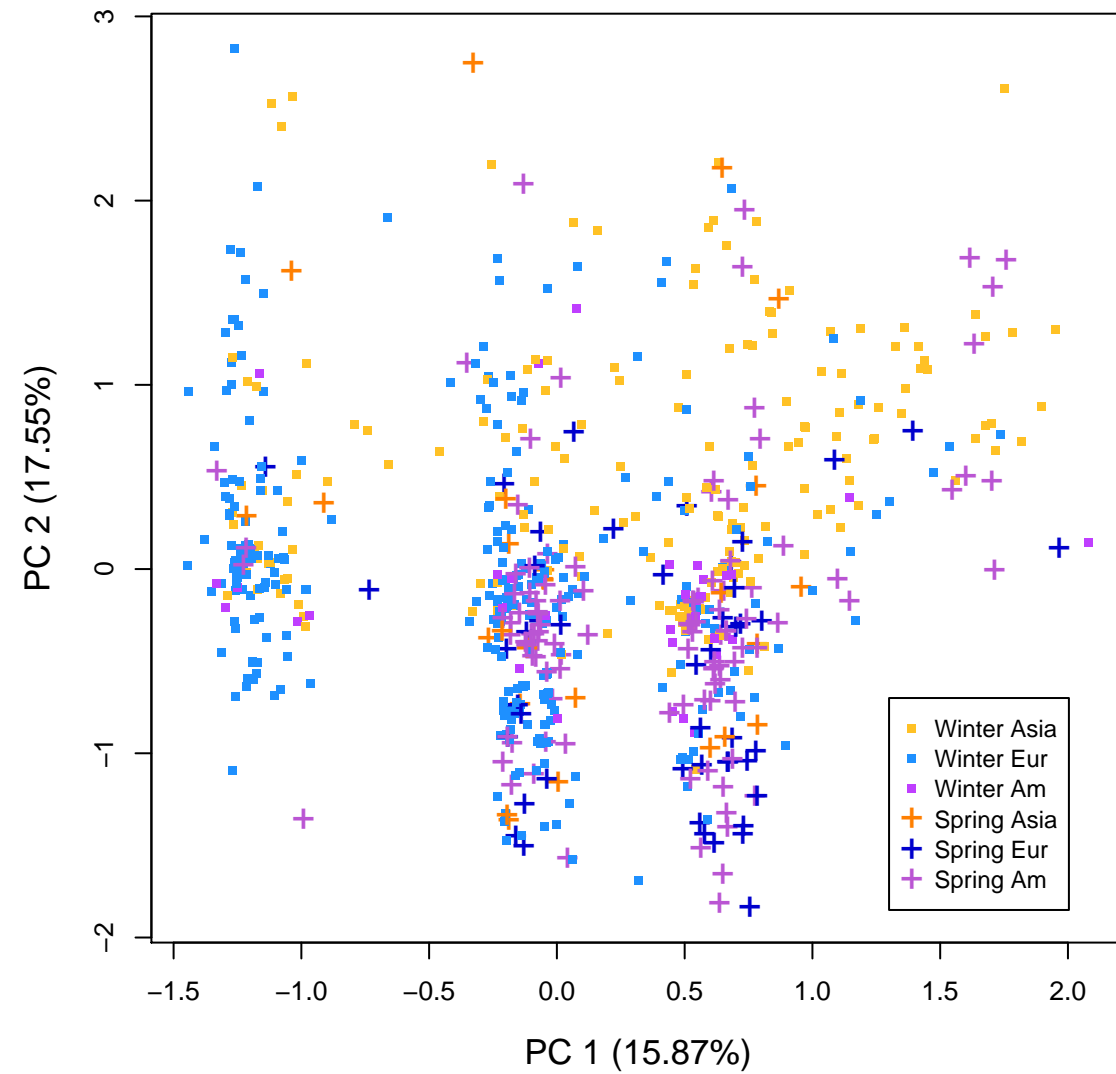

ROI 15

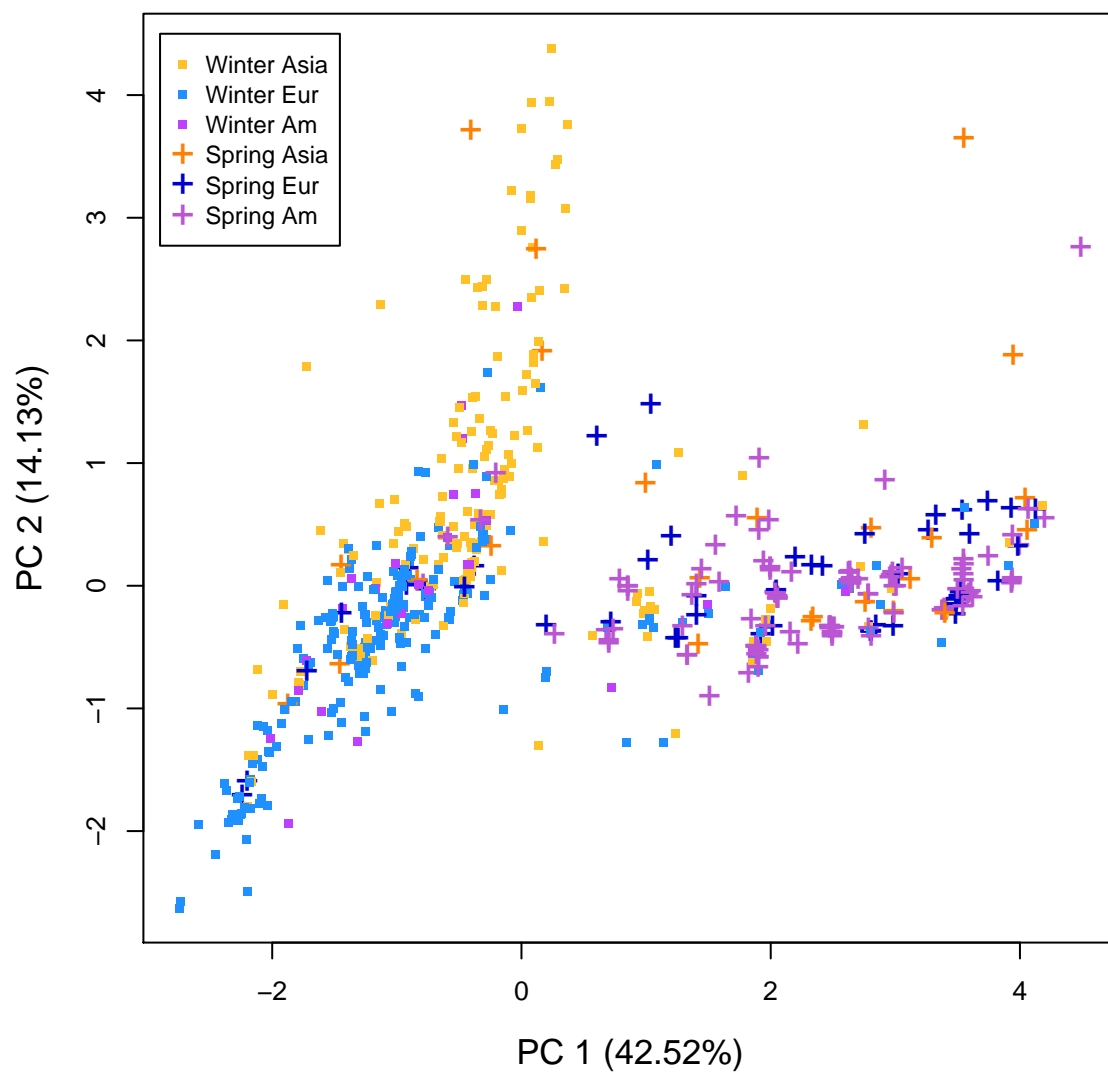

ROI 16

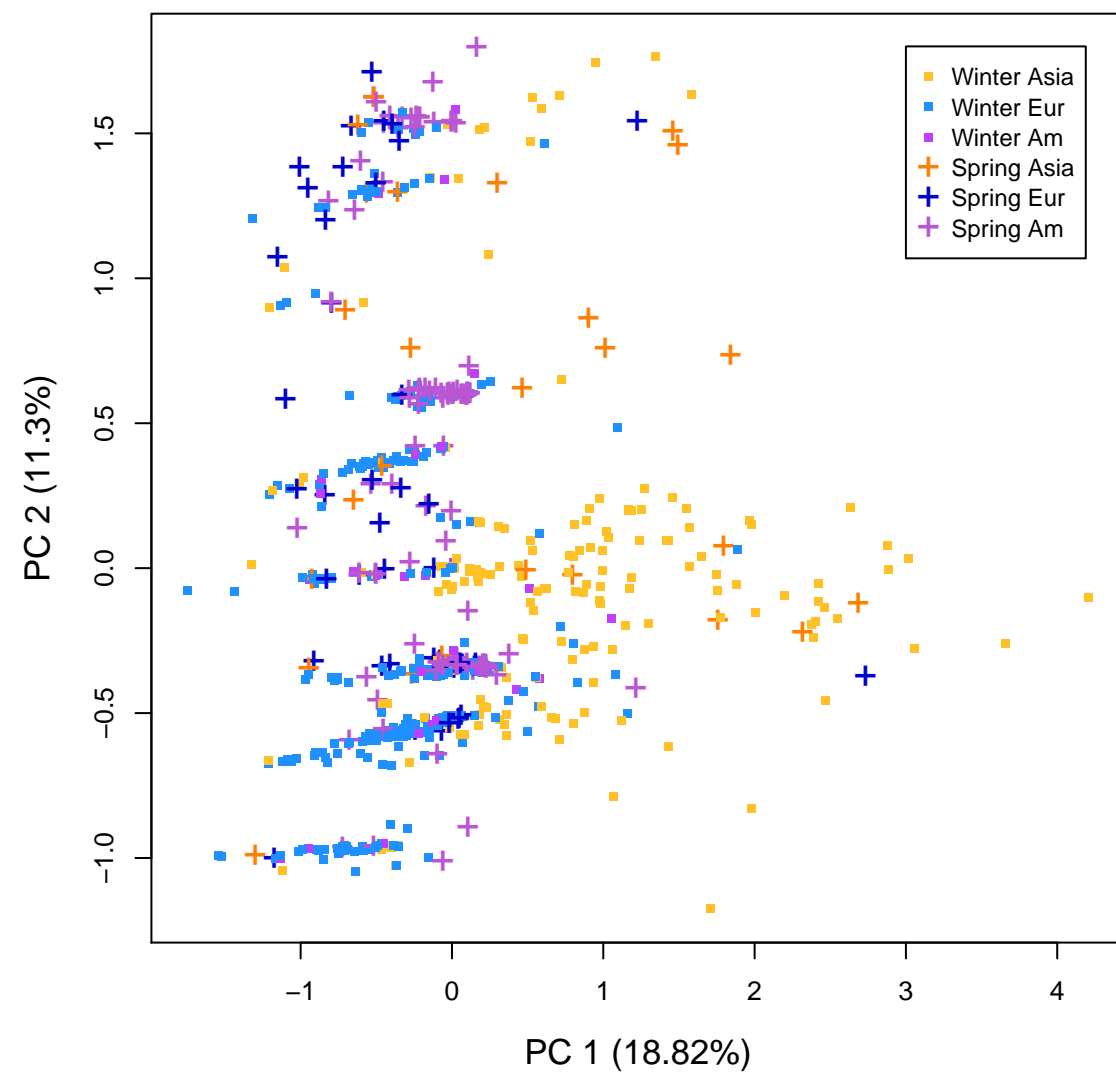

Supplement: Supplementary file 15 [file Image_8.PDF]

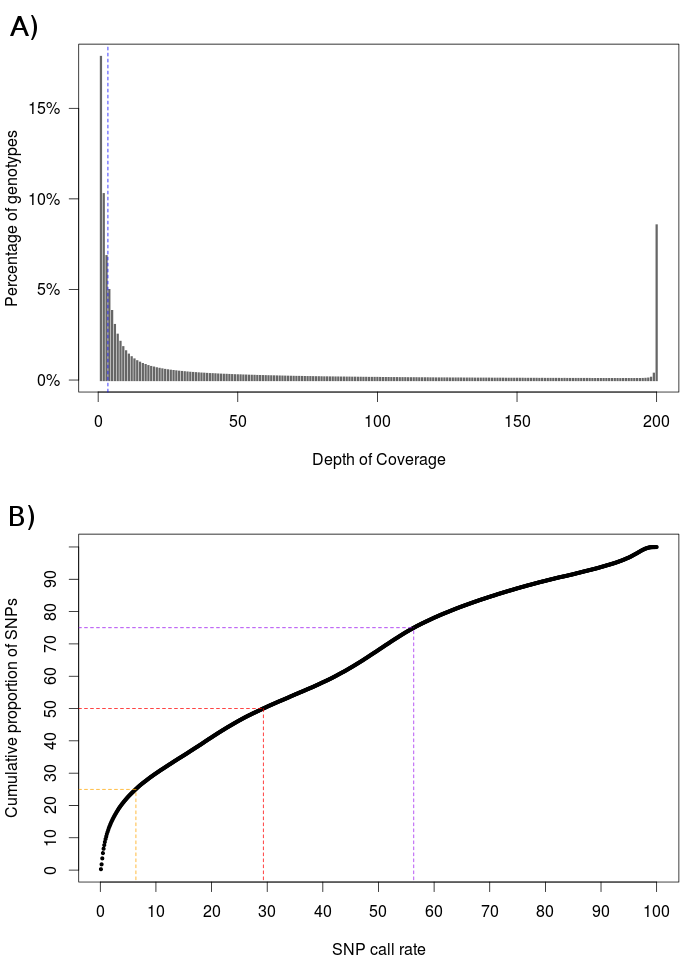

Supplement: Supplementary file 16 [file Image_9.TIFF]

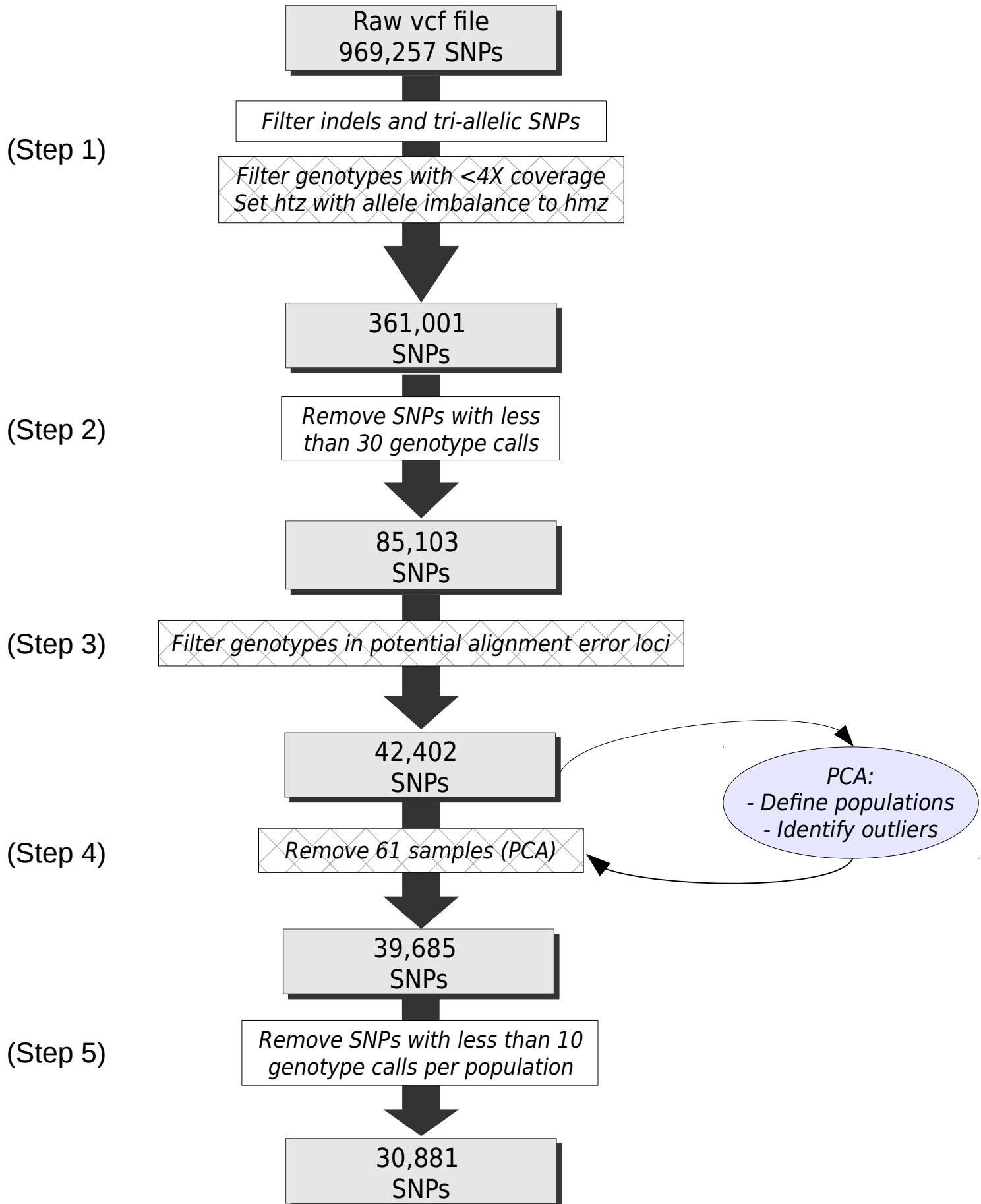

Supplement: Supplementary file 17 [file Image_10.PDF]

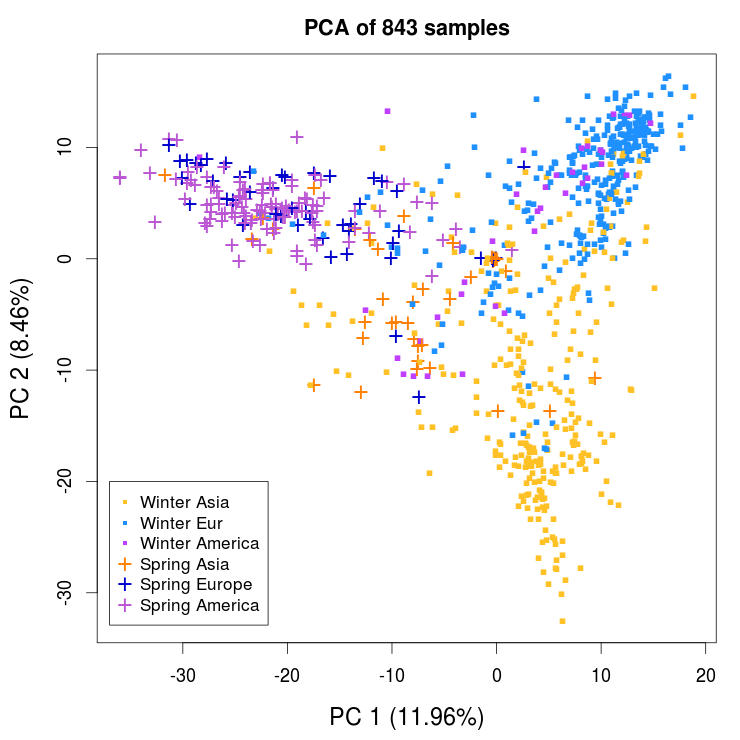

Supplement: Supplementary file 19 [file Image_12.TIFF]

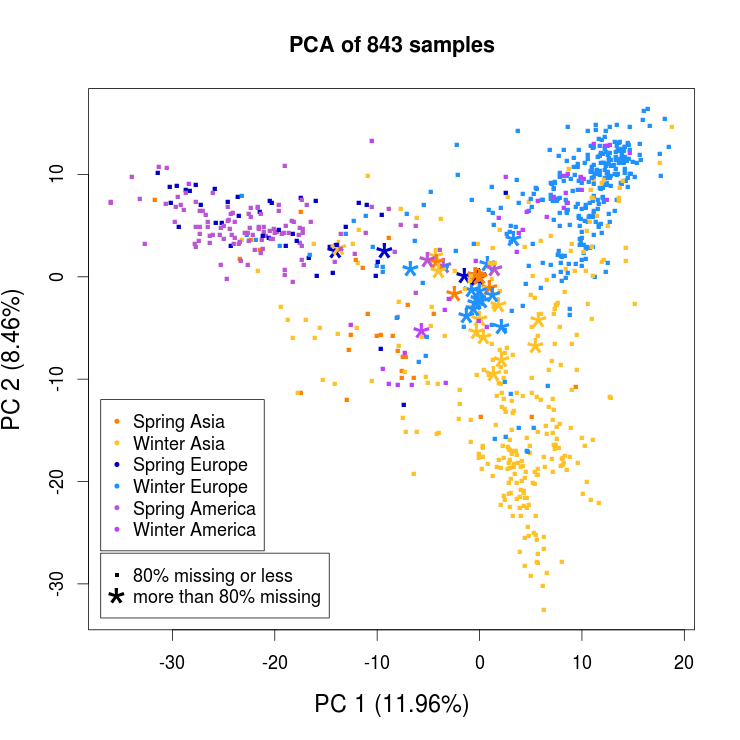

Supplement: Supplementary file 20 [file Image_13.TIFF]
