# Supplementary material for: Population Genomic Analysis Reveals Differential Evolutionary Histories and Patterns of Diversity across Subgenomes and Subpopulations of Brassica napus L
Source: Front Plant Sci. 2016 Apr 21;7:525. doi: 10.3389/fpls.2016.00525 (PMC4838616; doi:10.3389/fpls.2016.00525)
Supplement: Supplementary file 14 [file Image_7.PDF]

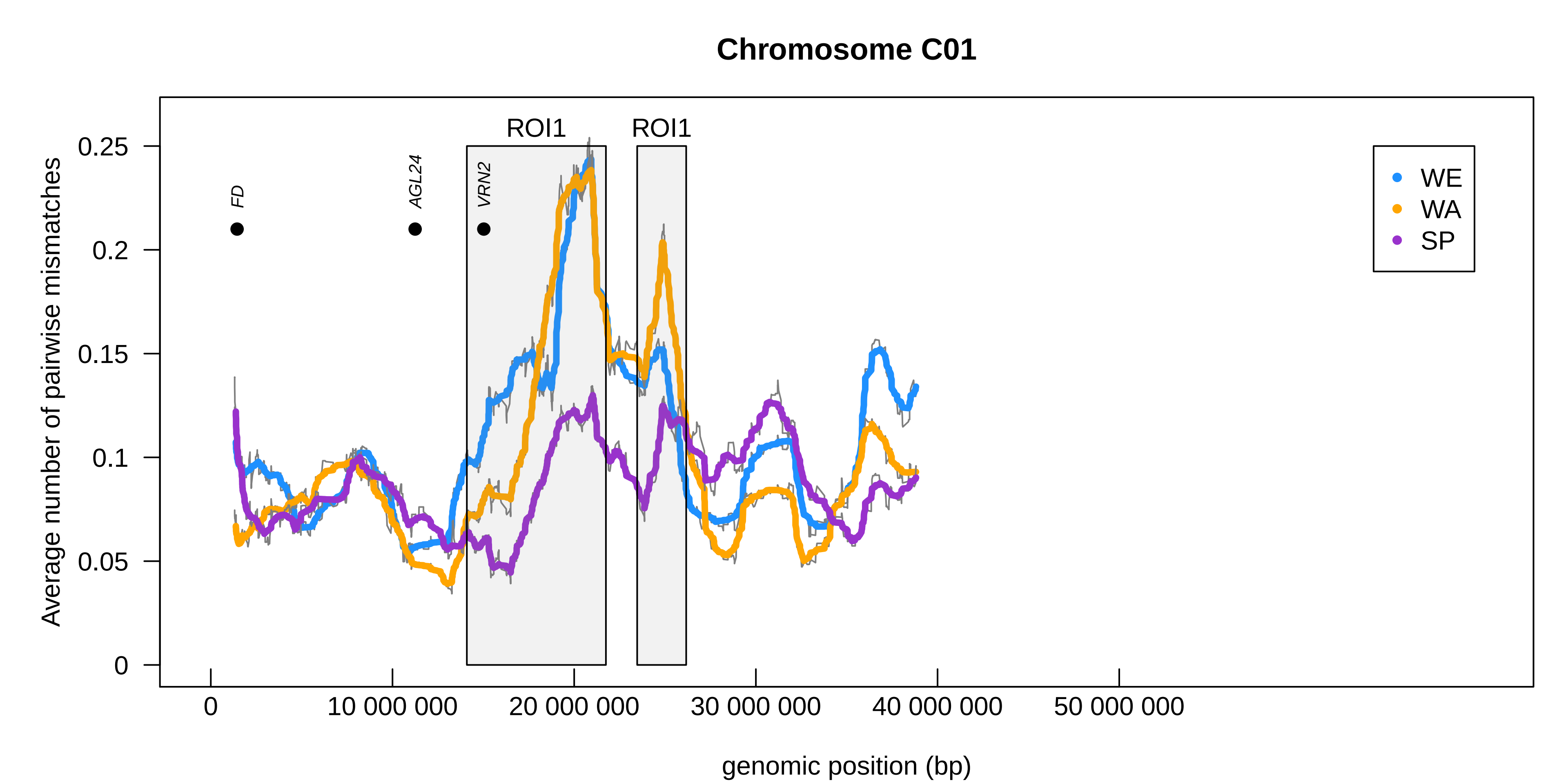

Chromosome C01
ROI1
ROI1
FD
AGL24
VRN2
WE
WA
SP
Average number of pairwise mismatches
0
0.05
0.1
0.15
0.2
0.25
0
10 000 000
20 000 000
30 000 000
40 000 000
50 000 000
genomic position (bp)

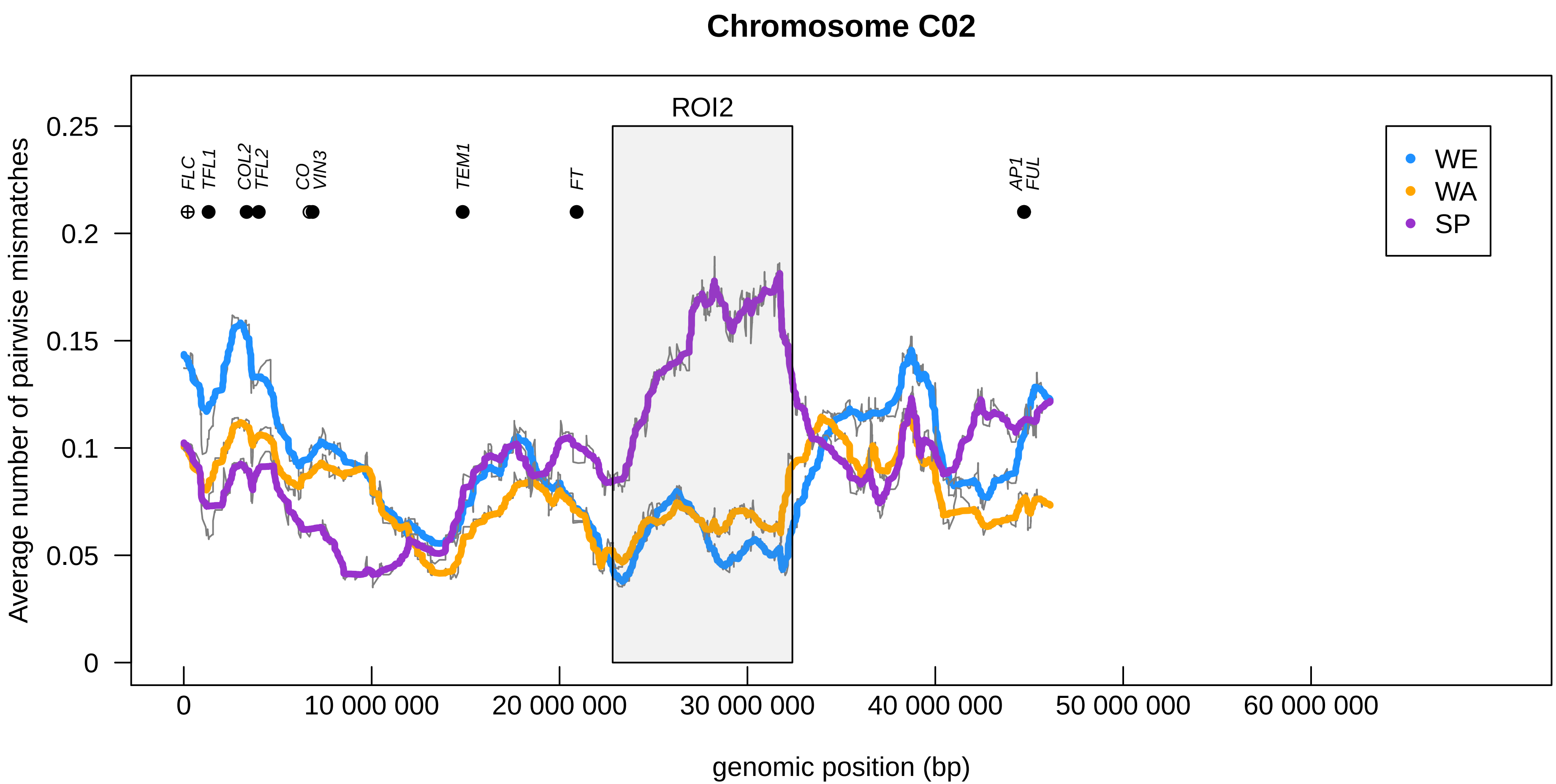

Chromosome C02
ROI2
WE
WA
SP
FLC
TFL1
COL2
TFL2
CO
VIN3
TEM1
FT
AP1
FUL
Average number of pairwise mismatches
0
0.05
0.1
0.15
0.2
0.25
0
10 000 000
20 000 000
30 000 000
40 000 000
50 000 000
60 000 000
genomic position (bp)

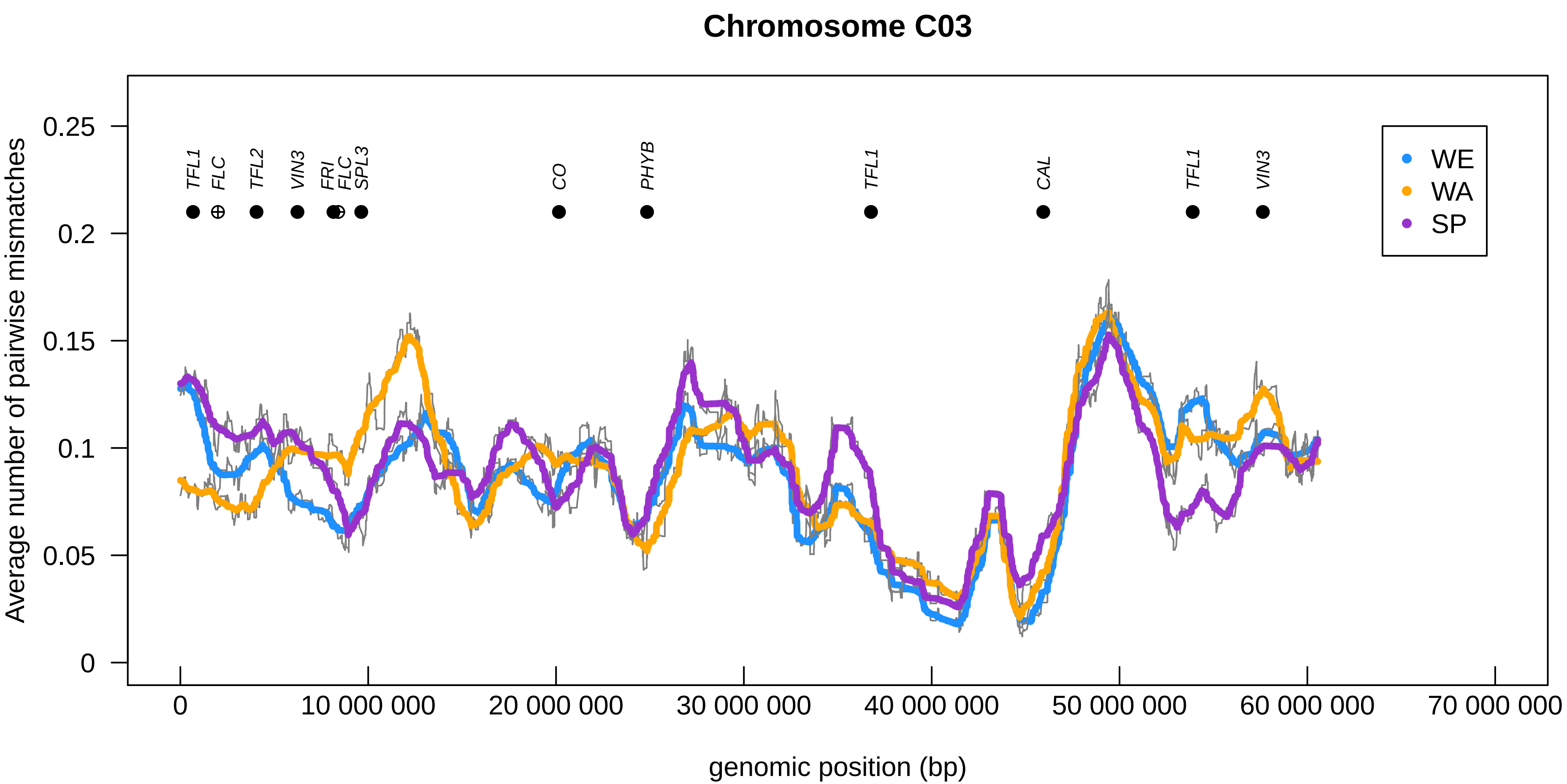

Chromosome C03
Average number of pairwise mismatches
genomic position (bp)
0
0.05
0.1
0.15
0.2
0.25
0
10 000 000
20 000 000
30 000 000
40 000 000
50 000 000
60 000 000
70 000 000
TFL1
FLC
TFL2
VIN3
FRI
FLC
SPL3
CO
PHYB
TFL1
CAL
TFL1
VIN3
WE
WA
SP

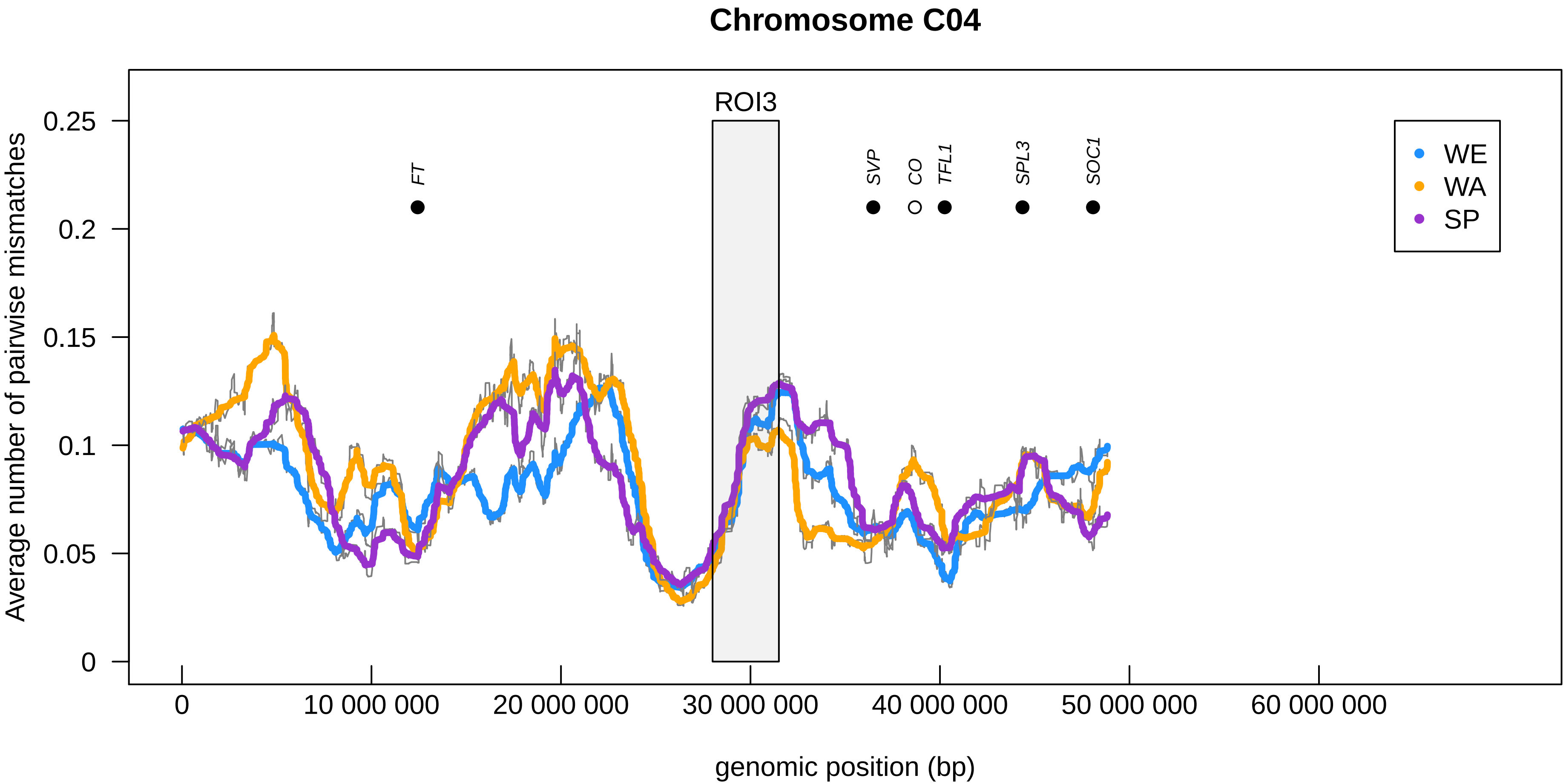

Chromosome C04
ROI3
FT
SVP
CO
TFL1
SPL3
SOC1
WE
WA
SP
Average number of pairwise mismatches
0.25
0.2
0.15
0.1
0.05
0
0
10 000 000
20 000 000
30 000 000
40 000 000
50 000 000
60 000 000
genomic position (bp)

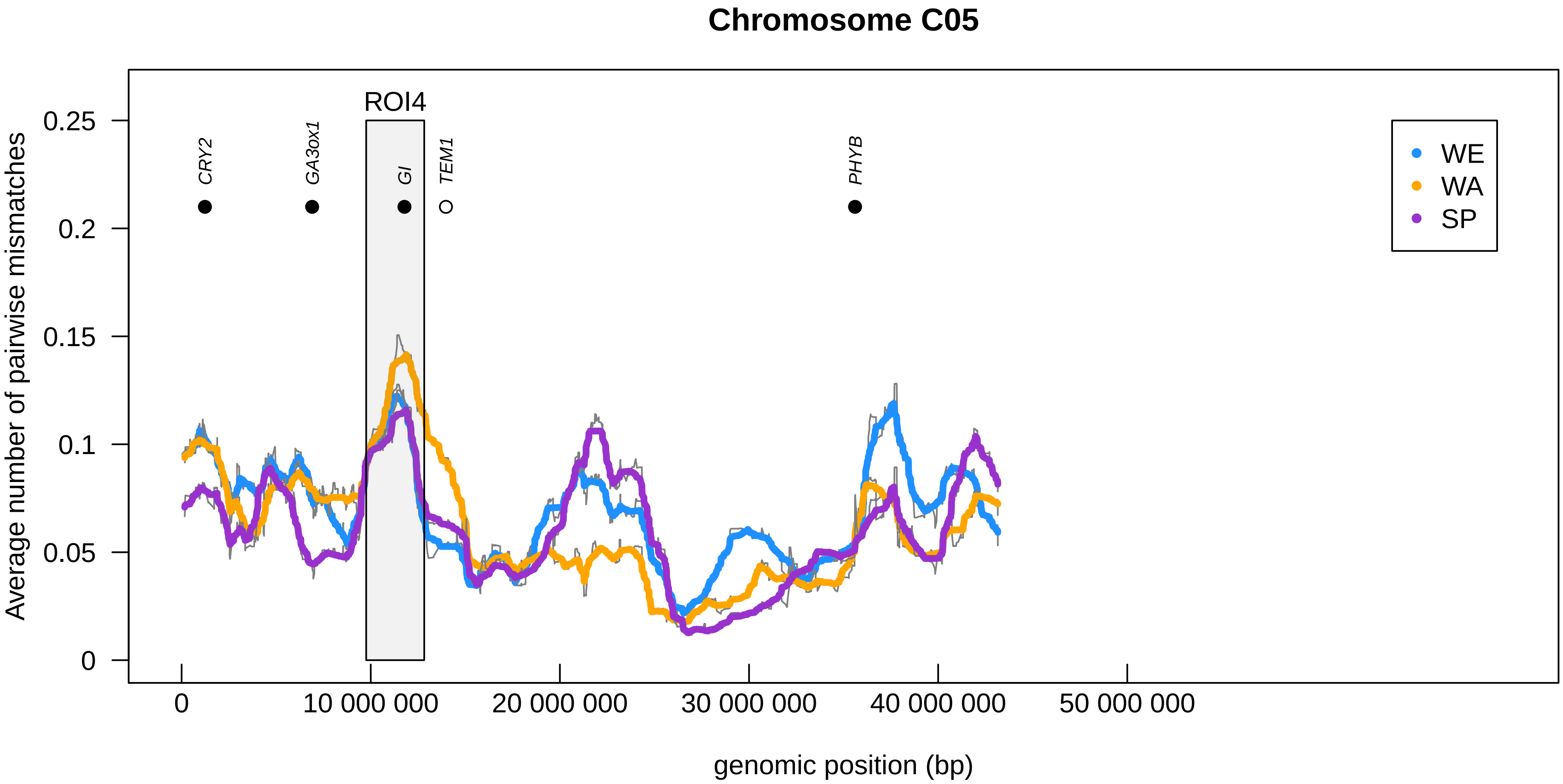

Chromosome C05
ROI4
CRY2
GA3ox1
GI
TEM1
PHYB
WE
WA
SP
Average number of pairwise mismatches
0.25
0.2
0.15
0.1
0.05
0
0
10 000 000
20 000 000
30 000 000
40 000 000
50 000 000
genomic position (bp)

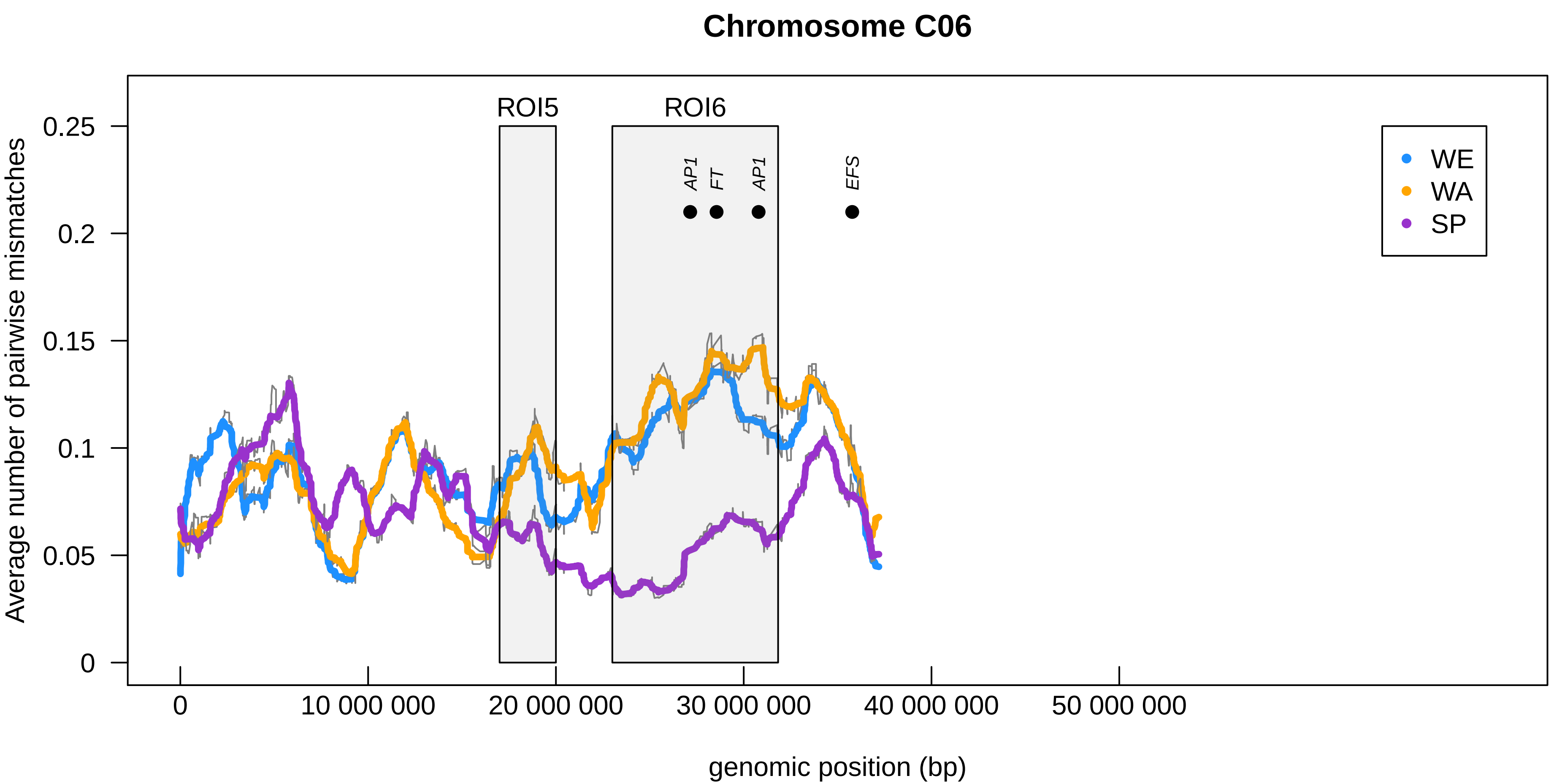

Chromosome C06
Average number of pairwise mismatches
0.25
0.2
0.15
0.1
0.05
0
ROI5
ROI6
AP1
FT
AP1
EFS
WE
WA
SP
0
10 000 000
20 000 000
30 000 000
40 000 000
50 000 000
genomic position (bp)

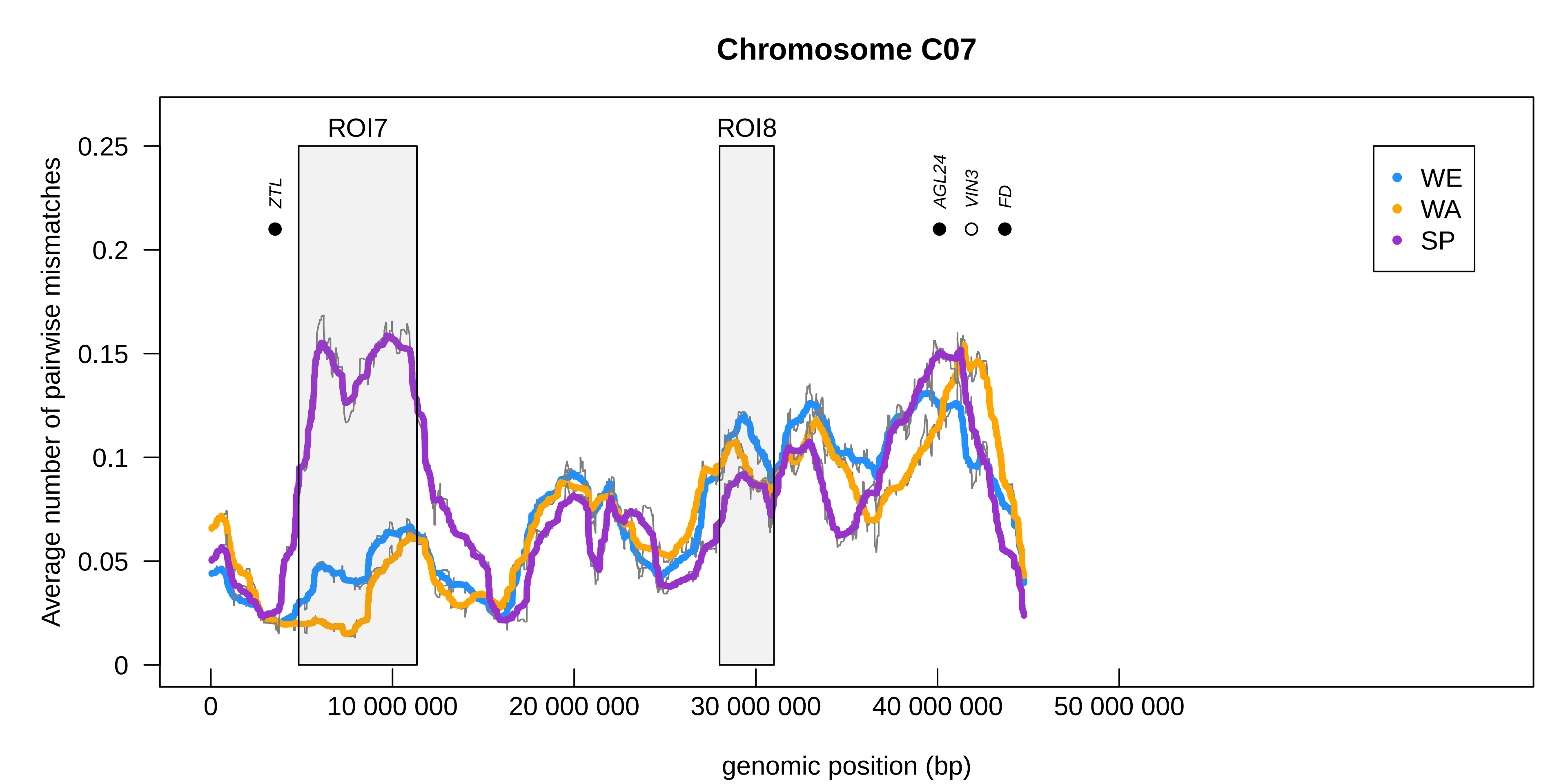
Chromosome C07
ROI7
ROI8
ZTL
AGL24
VIN3
FD
WE
WA
SP
0.25
0.2
0.15
0.1
0.05
0
Average number of pairwise mismatches
0
10 000 000
20 000 000
30 000 000
40 000 000
50 000 000
genomic position (bp)

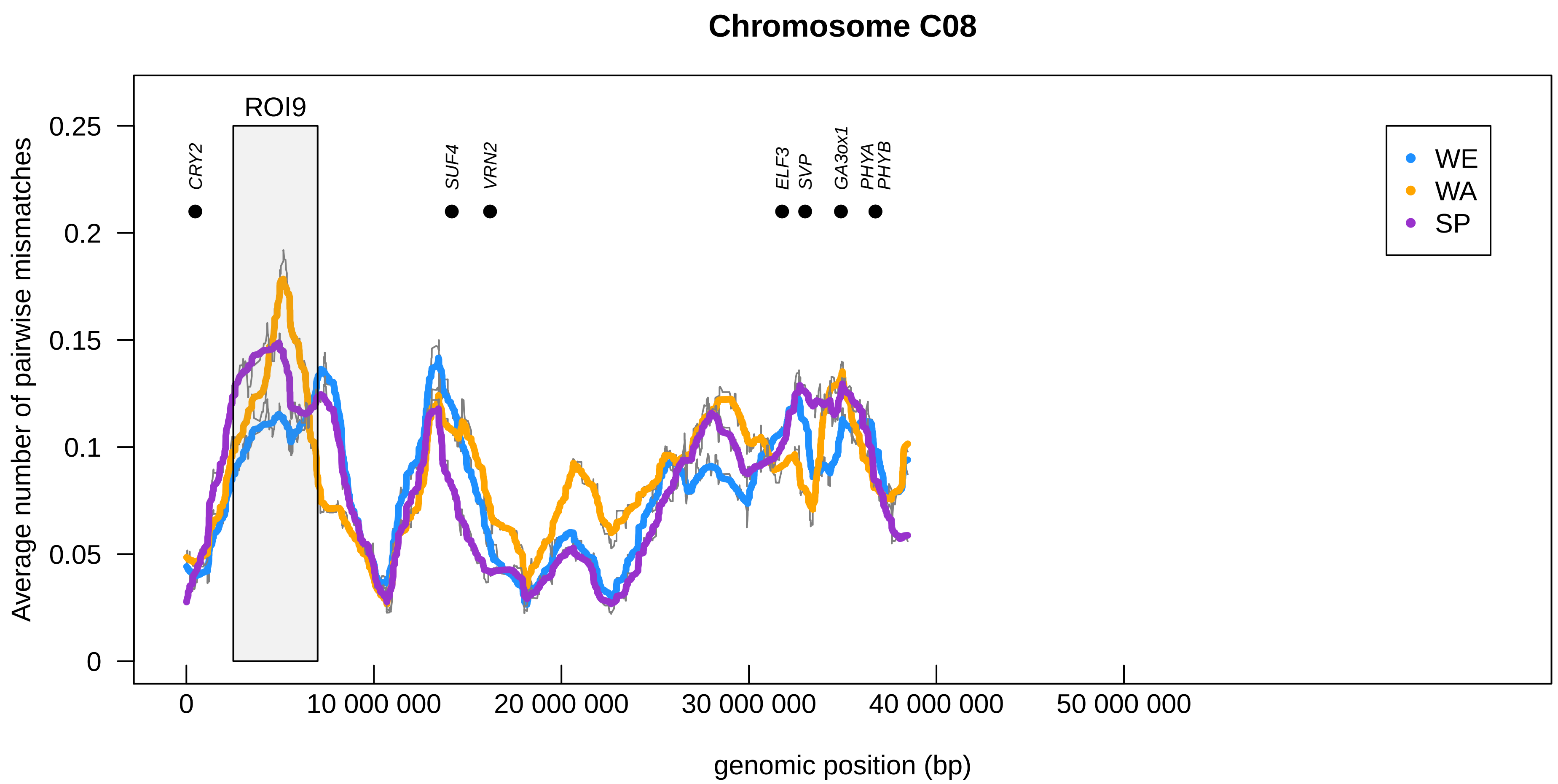
Chromosome C08
ROI9
CRY2
SUF4
VRN2
ELF3
SVP
GA3ox1
PHYA
PHYB
WE
WA
SP
Average number of pairwise mismatches
0
0.05
0.1
0.15
0.2
0.25
0
10 000 000
20 000 000
30 000 000
40 000 000
50 000 000
genomic position (bp)

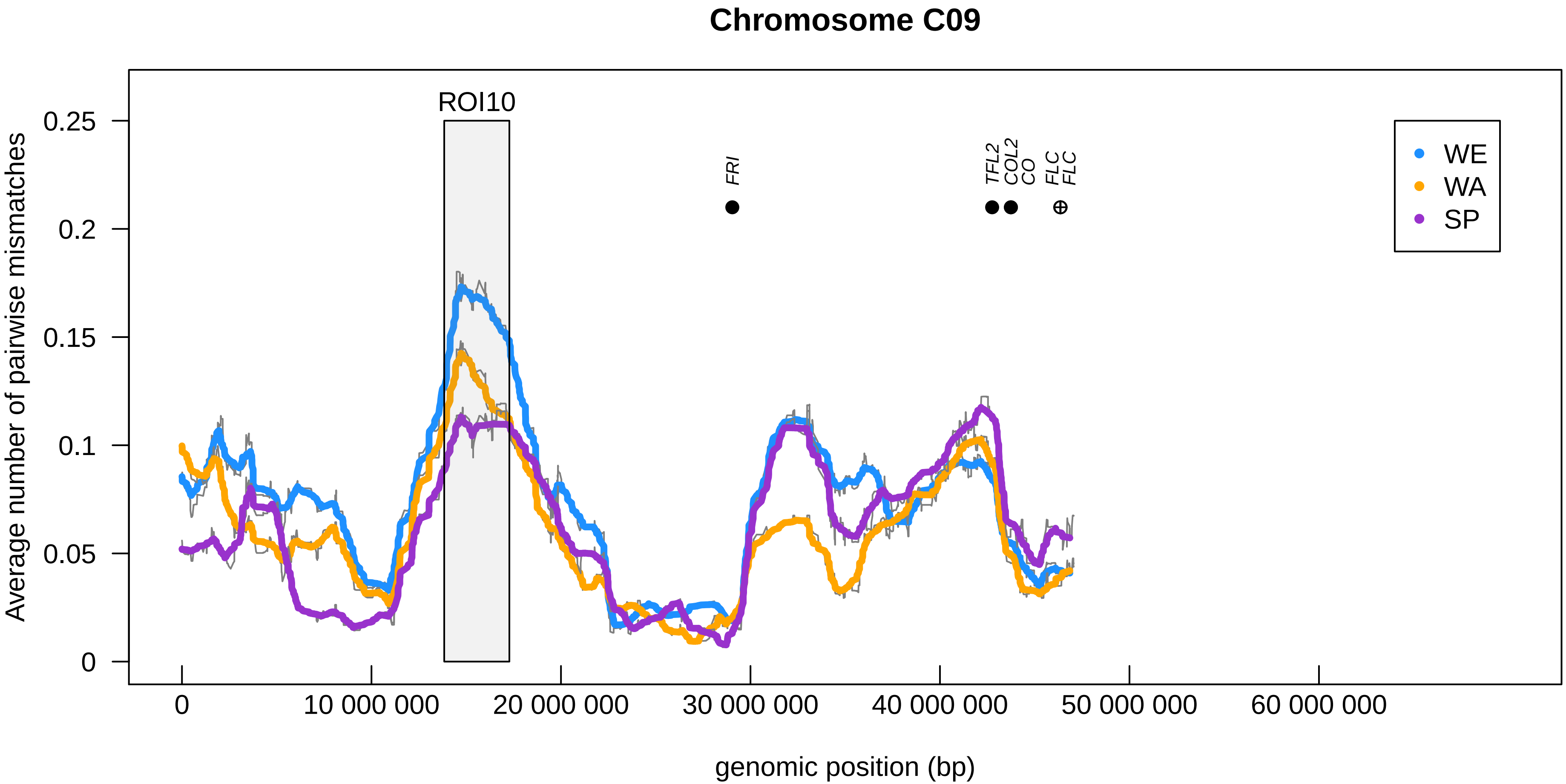

Chromosome C09
ROI10
FRI
TFL2
COL2
CO
FLC
FLC
WE
WA
SP
Average number of pairwise mismatches
0.25
0.2
0.15
0.1
0.05
0
0
10 000 000
20 000 000
30 000 000
40 000 000
50 000 000
60 000 000
genomic position (bp)

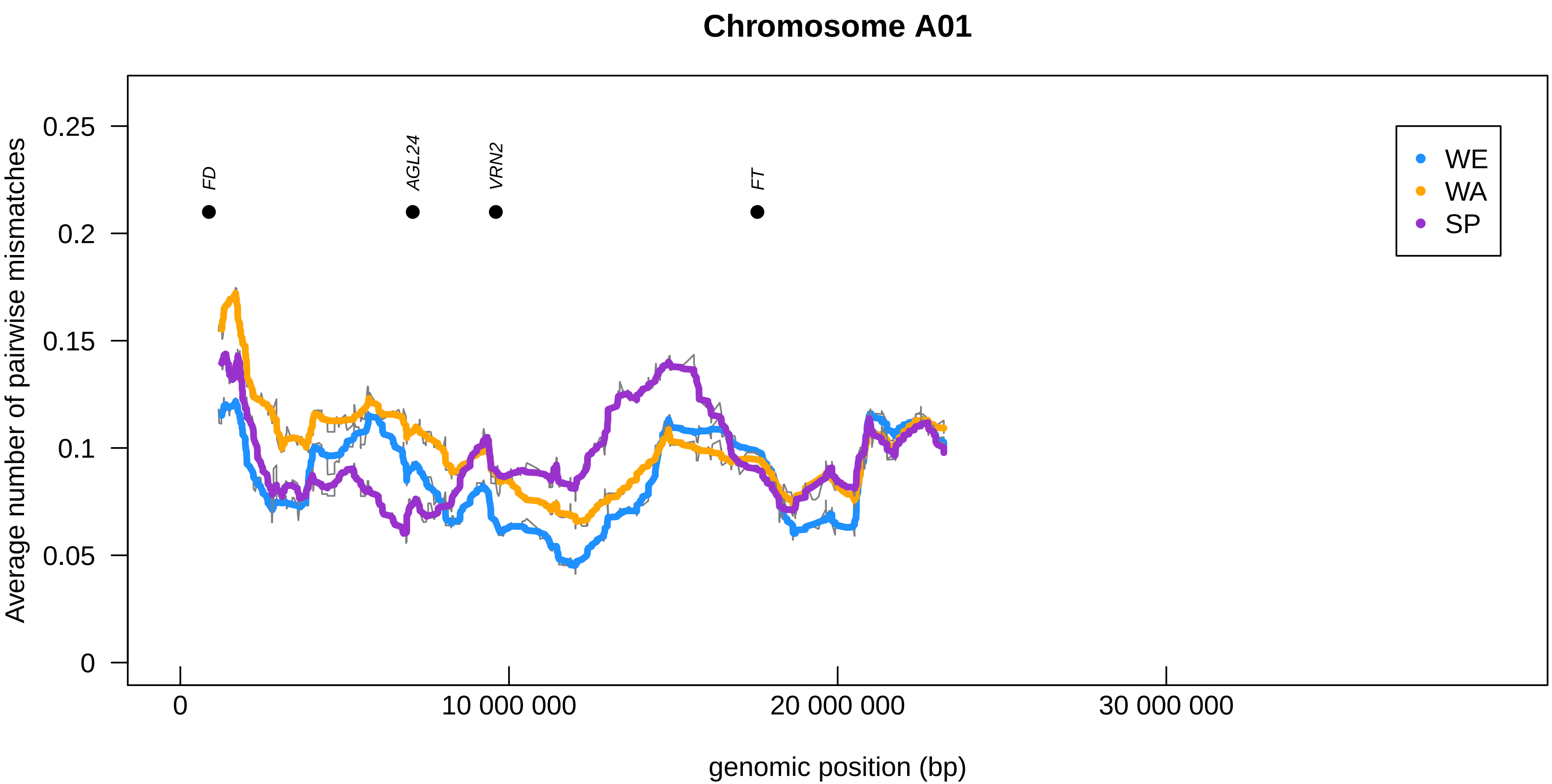

Chromosome A01
Average number of pairwise mismatches
0
0.05
0.1
0.15
0.2
0.25
genomic position (bp)
0
10 000 000
20 000 000
30 000 000
FD
AGL24
VRN2
FT
WE
WA
SP

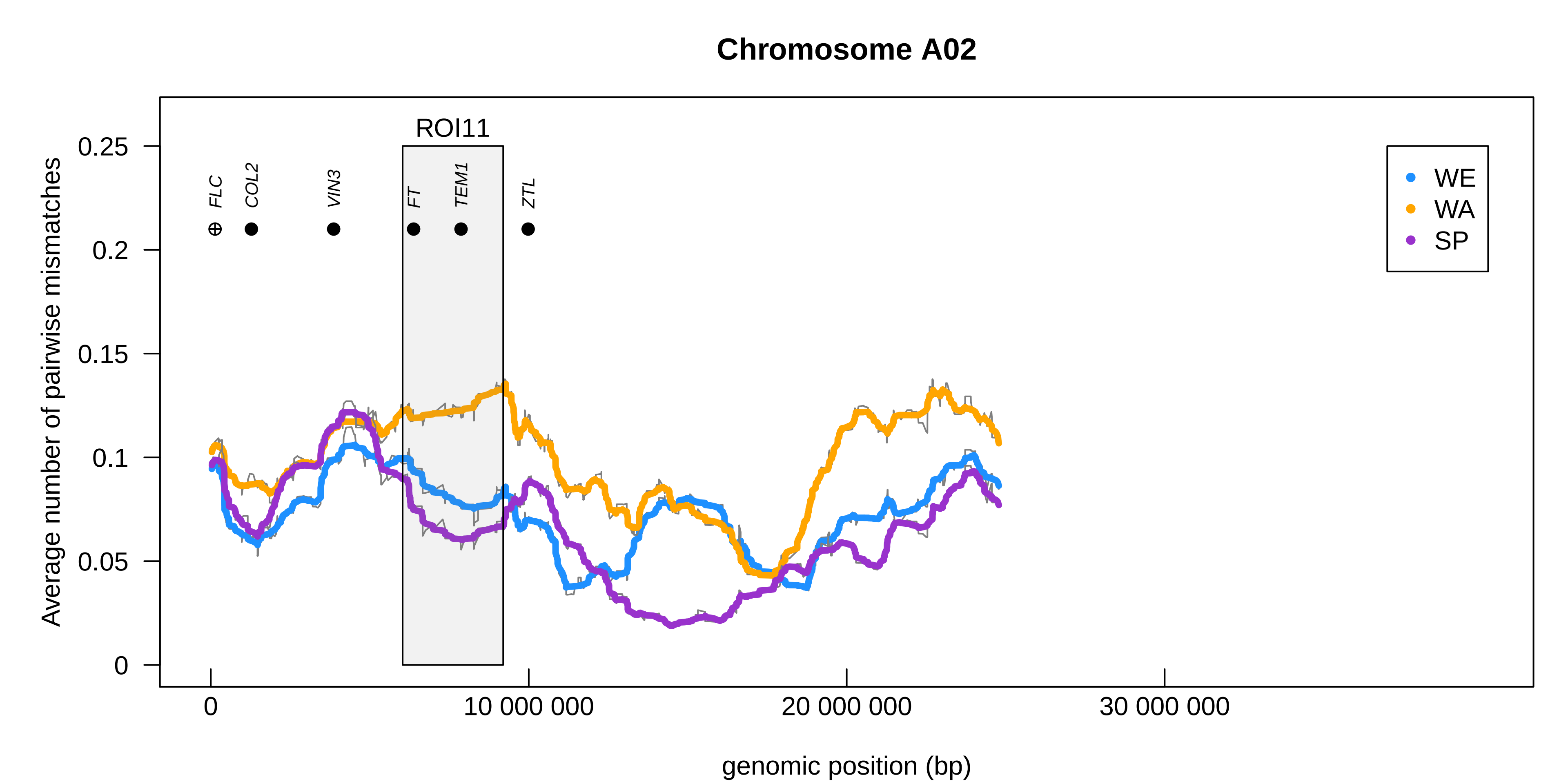

Chromosome A02
ROI11
FLC
COL2
VIN3
FT
TEM1
ZTL
WE
WA
SP
Average number of pairwise mismatches
0
0.05
0.1
0.15
0.2
0.25
0
10 000 000
20 000 000
30 000 000
genomic position (bp)

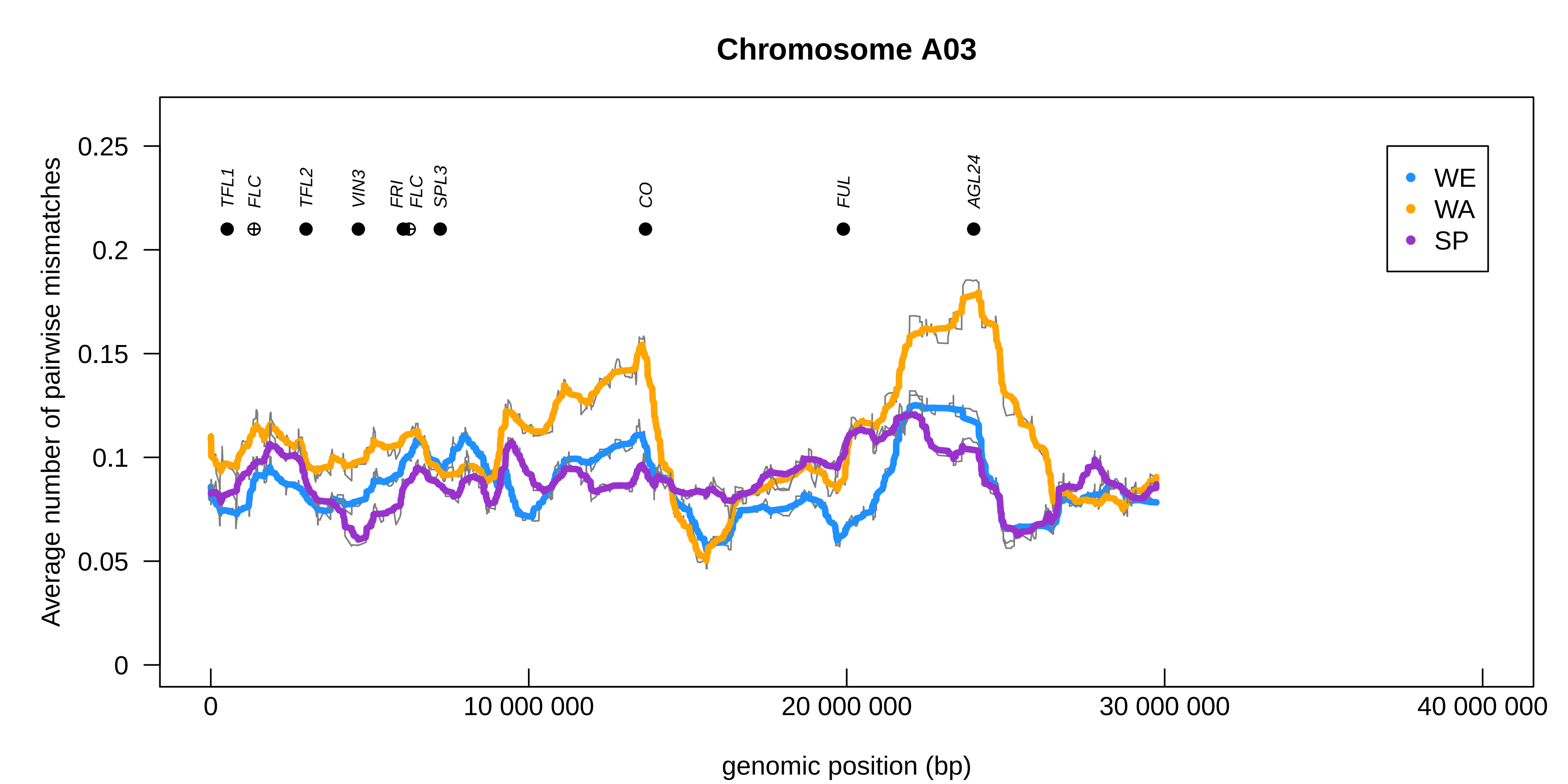

Chromosome A03
Average number of pairwise mismatches
genomic position (bp)
0.25
0.2
0.15
0.1
0.05
0
0
10 000 000
20 000 000
30 000 000
40 000 000
TFL1
FLC
TFL2
VIN3
FRI
FLC
SPL3
CO
FUL
AGL24
WE
WA
SP

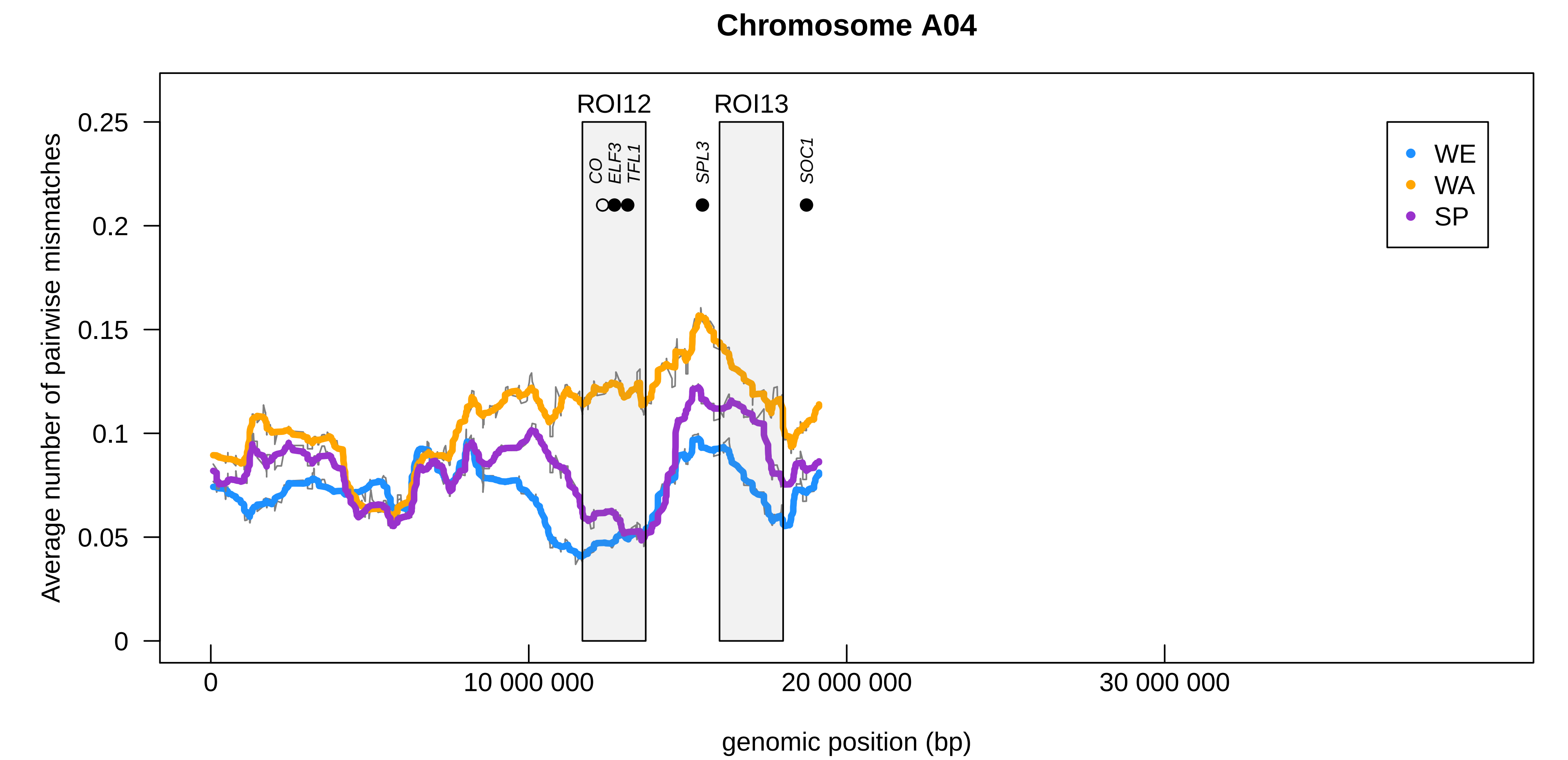
Chromosome A04
ROI12
ROI13
CO
ELF3
TFL1
SPL3
SOC1
WE
WA
SP
Average number of pairwise mismatches
0.25
0.2
0.15
0.1
0.05
0
0
10 000 000
20 000 000
30 000 000
genomic position (bp)

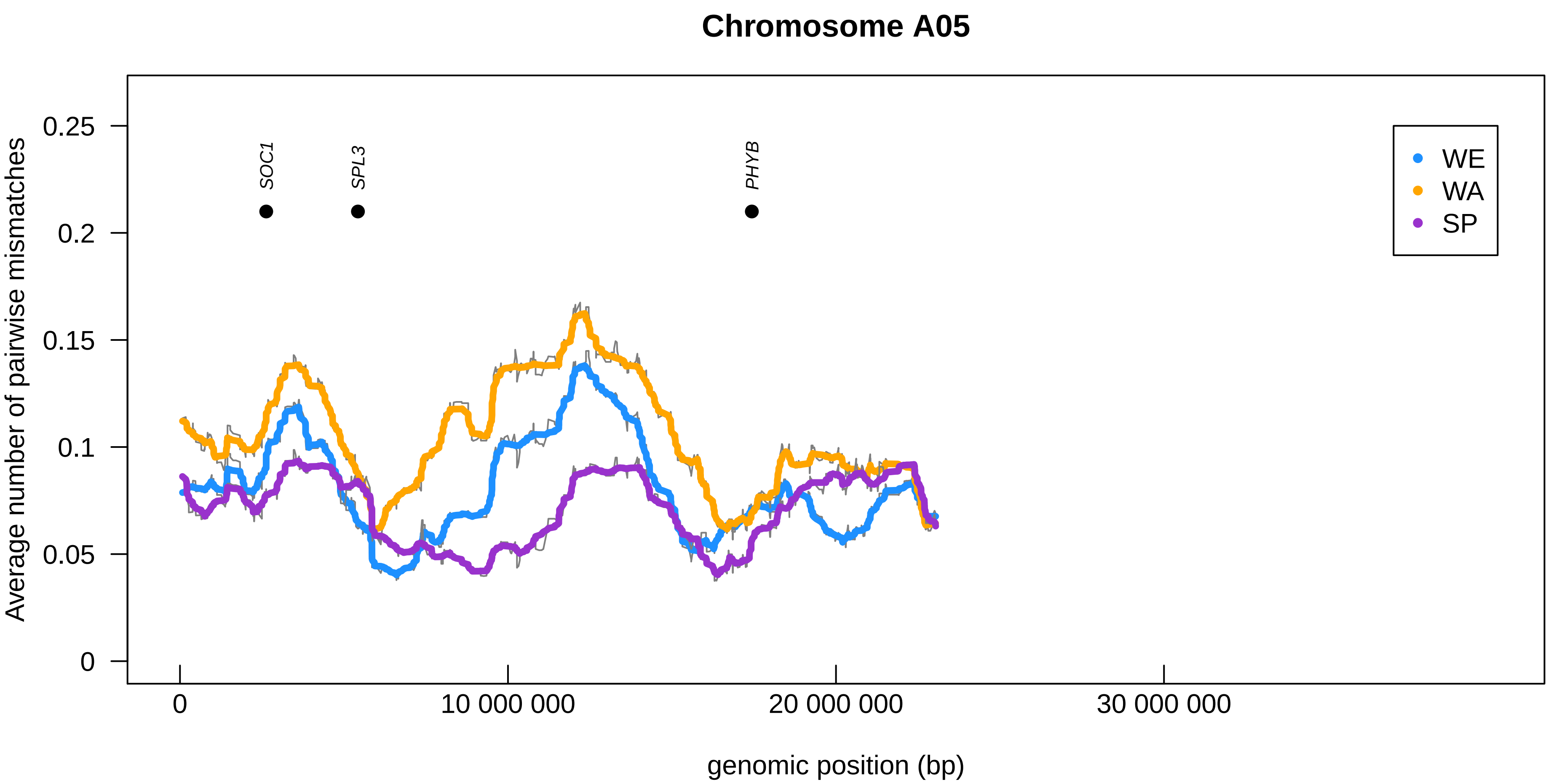
Chromosome A05
Average number of pairwise mismatches
genomic position (bp)
0
0.05
0.1
0.15
0.2
0.25
0
10 000 000
20 000 000
30 000 000
SOC1
SPL3
PHYB
WE
WA
SP

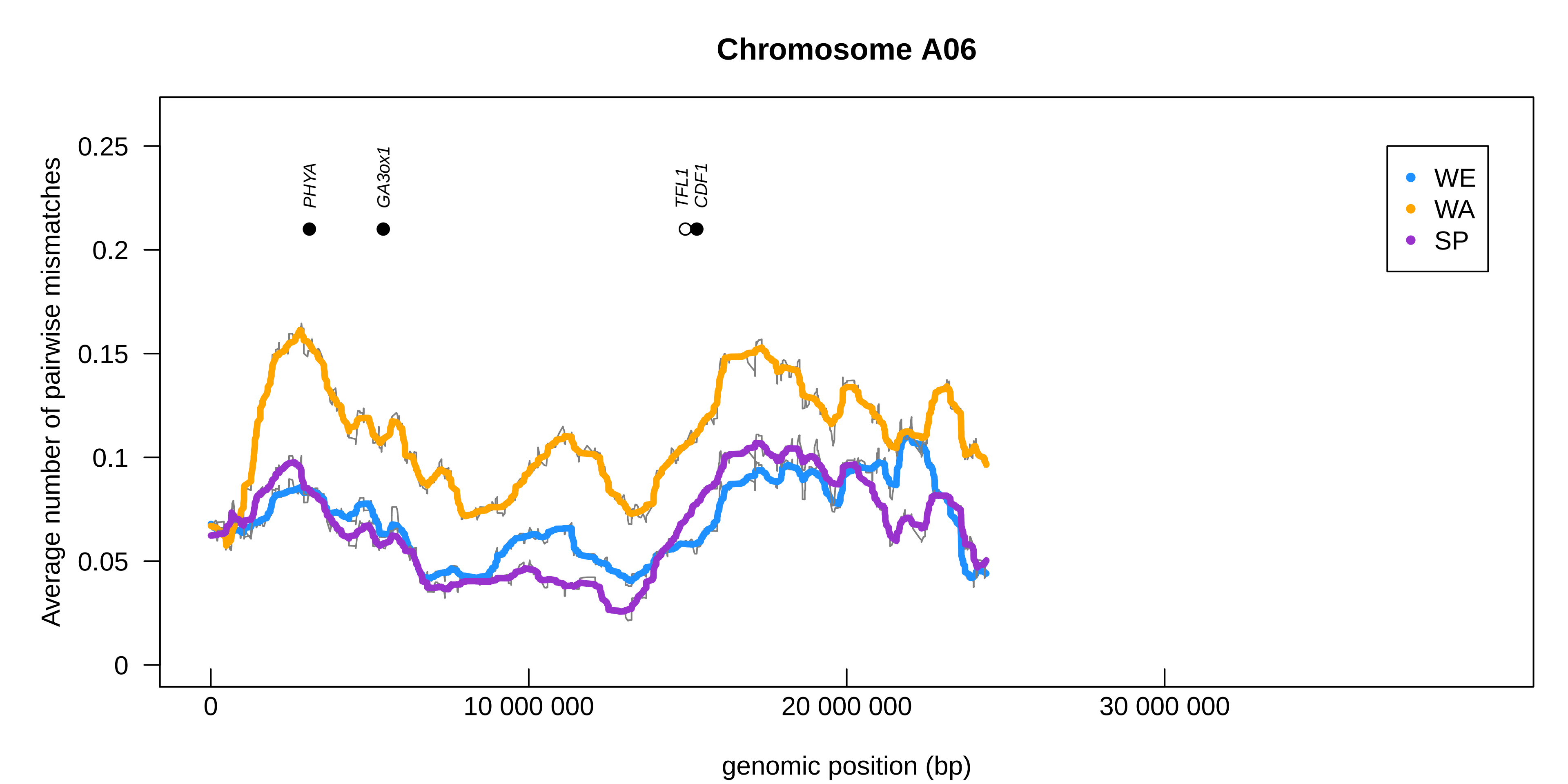
Chromosome A06
Average number of pairwise mismatches
0.25
0.2
0.15
0.1
0.05
0
PHYA
GA3ox1
TFL1
CDF1
WE
WA
SP
0
10 000 000
20 000 000
30 000 000
genomic position (bp)

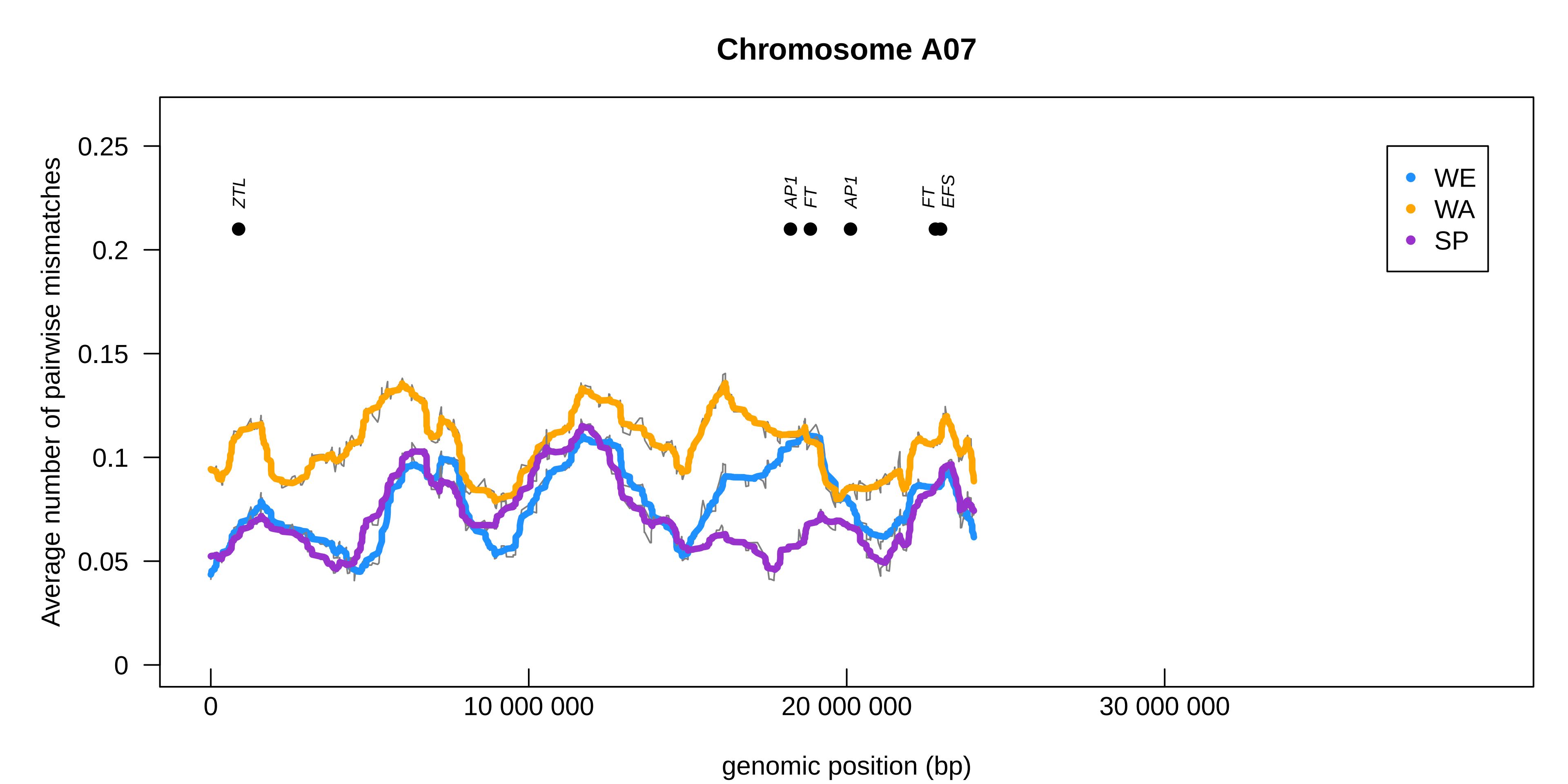
Chromosome A07
Average number of pairwise mismatches
0.25
0.2
0.15
0.1
0.05
0
0
10 000 000
20 000 000
30 000 000
genomic position (bp)
ZTL
AP1
FT
AP1
FT
EFS
WE
WA
SP

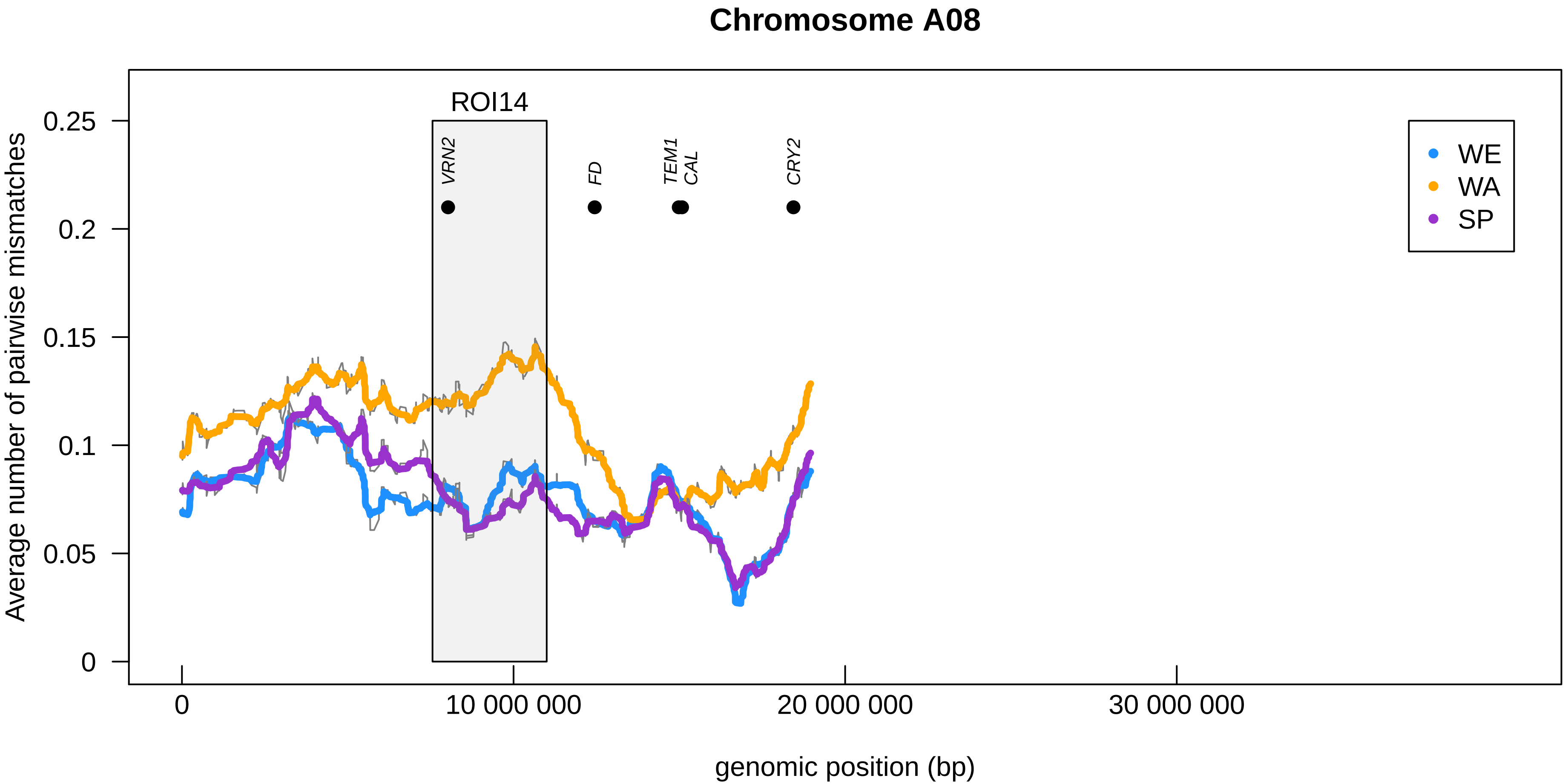
Chromosome A08
ROI14
VRN2
FD
TEM1
CAL
CRY2
WE
WA
SP
Average number of pairwise mismatches
0.25
0.2
0.15
0.1
0.05
0
0
10 000 000
20 000 000
30 000 000
genomic position (bp)

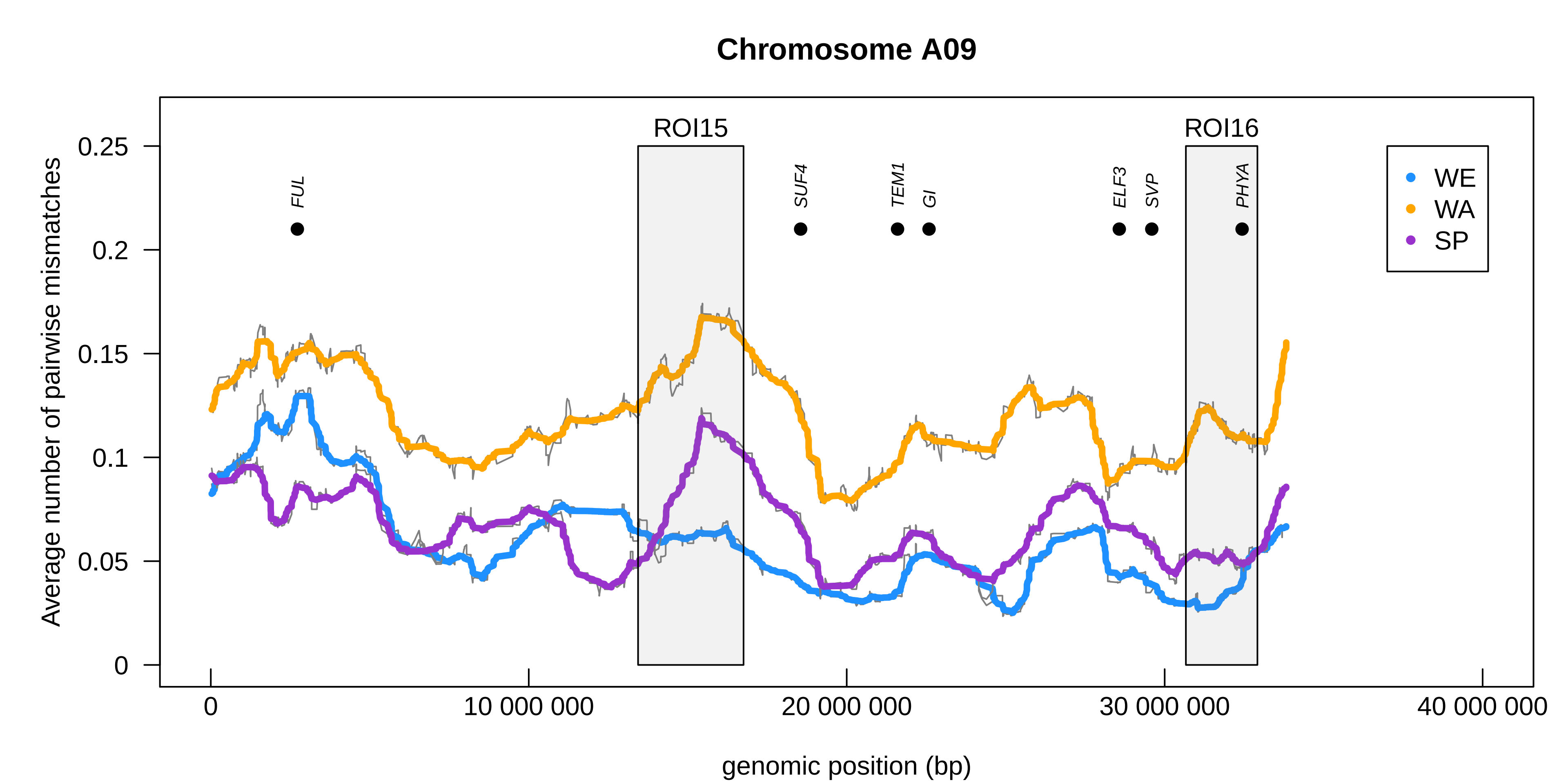
Chromosome A09
Average number of pairwise mismatches
0
0.05
0.1
0.15
0.2
0.25
ROI15
ROI16
FUL
SUF4
TEM1
GI
ELF3
SVP
PHYA
WE
WA
SP
0
10 000 000
20 000 000
30 000 000
40 000 000
genomic position (bp)

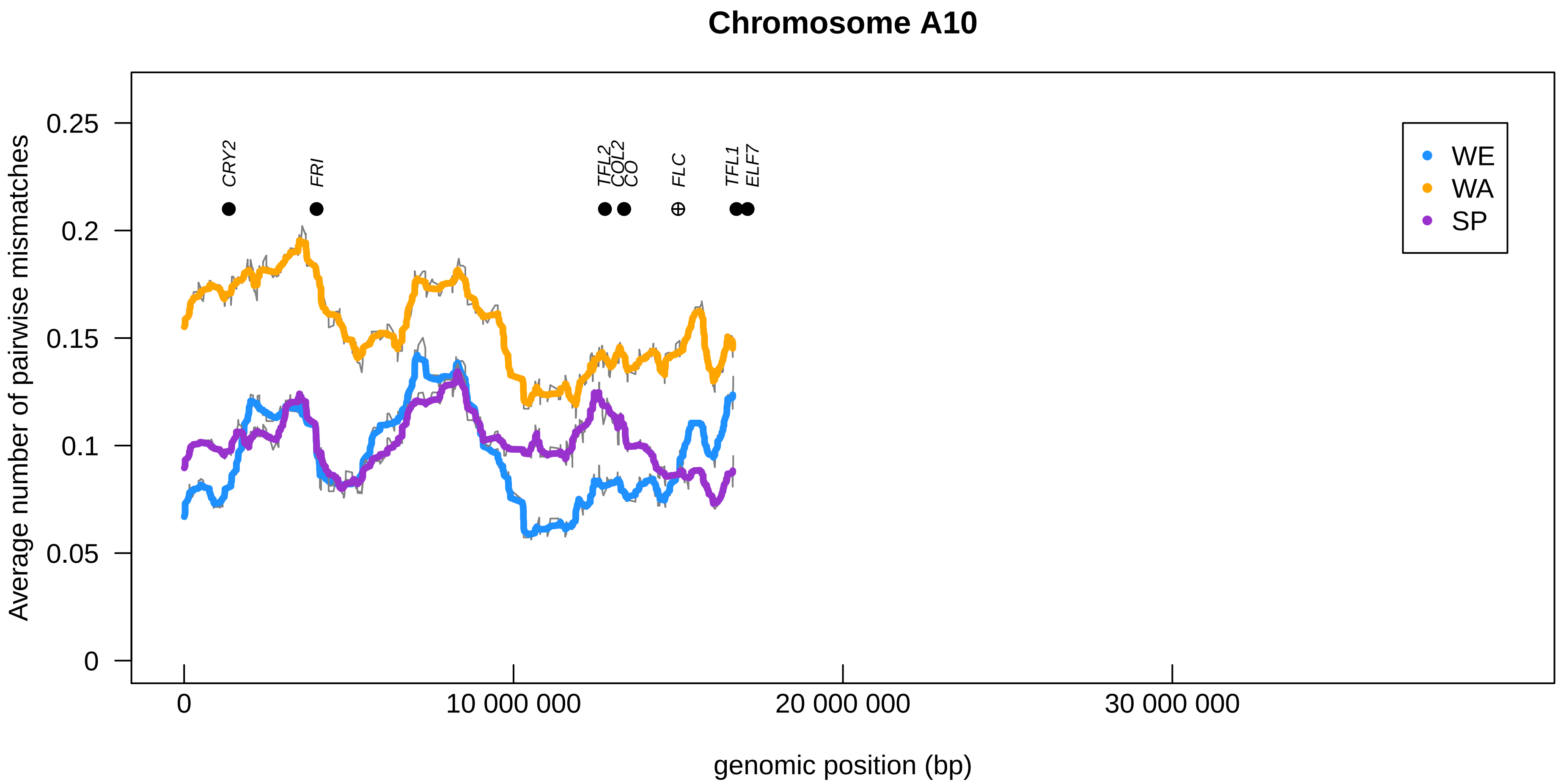

Chromosome A10
Average number of pairwise mismatches
0.25
0.2
0.15
0.1
0.05
0
CRY2
FRI
TFL2
COL2
CO
FLC
TFL1
ELF7
WE
WA
SP
0
10 000 000
20 000 000
30 000 000
genomic position (bp)
